# Specific Transcriptome Changes Associated with Blood Pressure Reduction in Hypertensive Patients After Relaxation Response Training

**DOI:** 10.1089/acm.2017.0053

**Published:** 2018-05-01

**Authors:** Manoj K. Bhasin, John W. Denninger, Jeff C. Huffman, Marie G. Joseph, Halsey Niles, Emma Chad-Friedman, Roberta Goldman, Beverly Buczynski-Kelley, Barbara A. Mahoney, Gregory L. Fricchione, Jeffery A. Dusek, Herbert Benson, Randall M. Zusman, Towia A. Libermann

**Affiliations:** ^1^ Benson-Henry Institute for Mind Body Medicine, Massachusetts General Hospital, Boston, MA.; ^2^ Division of Interdisciplinary Medicine and Biotechnology, Department of Medicine, Beth Israel Deaconess Medical Center, Harvard Medical School, Boston, MA.; ^3^ BIDMC Genomics, Proteomics, Bioinformatics and Systems Biology Center, Beth Israel Deaconess Medical Center, Boston, MA.; ^4^ Department of Psychiatry, Massachusetts General Hospital, Harvard Medical School, Boston, MA.; ^5^ Department of Medicine, Corrigan-Minehan Heart Center, Cardiology Division, Section on Hypertension, Massachusetts General Hospital, Harvard Medical School, Boston, MA.; ^6^ Integrative Health Research Center, Penny George Institute for Health and Healing, Allina Health, Minneapolis, MN.; ^7^ Department of Medicine, Massachusetts General Hospital, Harvard Medical School, Boston, MA.

**Keywords:** relaxation response, hypertension, gene expression, microarray, blood pressure, mindfulness

## Abstract

***Objective:*** Mind–body practices that elicit the relaxation response (RR) have been demonstrated to reduce blood pressure (BP) in essential hypertension (HTN) and may be an adjunct to antihypertensive drug therapy. However, the molecular mechanisms by which the RR reduces BP remain undefined.

***Design:*** Genomic determinants associated with responsiveness to an 8-week RR-based mind–body intervention for lowering HTN in 13 stage 1 hypertensive patients classified as BP responders and 11 as nonresponders were identified.

***Results:*** Transcriptome analysis in peripheral blood mononuclear cells identified 1771 genes regulated by the RR in responders. Biological process- and pathway-based analysis of transcriptome data demonstrated enrichment in the following gene categories: immune regulatory pathways and metabolism (among downregulated genes); glucose metabolism, cardiovascular system development, and circadian rhythm (among upregulated genes). Further *in silico* estimation of cell abundance from the microarray data showed enrichment of the anti-inflammatory M2 subtype of macrophages in BP responders. Nuclear factor-κB, vascular endothelial growth factor, and insulin were critical molecules emerging from interactive network analysis.

***Conclusions:*** These findings provide the first insights into the molecular mechanisms that are associated with the beneficial effects of the RR on HTN.

## Introduction

Hypertension (HTN) is a major risk factor for the development of coronary, cerebrovascular, and peripheral vascular disease, leading to myocardial infarction, stroke, and vascular death.^1,2^ As many as 100 million Americans and up to 1 billion people worldwide are estimated to have HTN.^1,2^ Among older Americans, prevalence rises to >50%.^1^

HTN is of particular importance for public health as the total annual cost attributed to HTN is projected to rise to $274 billion by 2030.^1^ A recent article published in the *Journal of the American Medical Association* reported that elevated blood pressure (BP) is associated with increased burden of death globally.^1^ Pharmacologic therapy is the principal means of BP control; however, antihypertensive drugs are associated with side effects, which can be burdensome and can cause noncompliance with the prescribed antihypertensive regimen.^3,4^ Importantly, lifestyle modification (weight loss, exercise, and sodium intake restriction) and mind–body interventions (MBIs) may be at least as effective as pharmacologic therapy^5–7^ and are generally free of side effects. Recently, the American Heart Association (AHA) issued a statement about the potential usefulness of mediation, including the relaxation response (RR), for heart-healthy lifestyle, and medical treatment.^2^ This statement from the AHA specifically mentioned that meditation may help lower BP and decrease the risk of heart attacks.

The stress response is a state of physiologic, behavioral, and psychological hyperarousal in an organism in response to an environmental challenge. The profound physiologic alterations observable in the stress response—involving the hypothalamic–pituitary–adrenal axis and the sympathetic nervous system—may lead to onset, development, or progression of pathophysiologic disease processes,^8^ especially when stress is chronic or overwhelming.^9^ This clear relationship between stress and HTN^3–5^ is further supported by data showing that anxiety and depression are recognized risk factors for HTN development. HTN can be envisioned in part as a stress disorder, in which increased BP is the body's attempt to adapt to stress.^6^

Increasing evidence and meta-analysis of MBI trials support their efficacy in treating chronic diseases.^7–10^ One of these MBIs, the RR, is the physiologic and psychological opposite of the fight-or-flight stress response^11–13^ and has demonstrated efficacy in stress-linked disorders.^7,8^ The RR is evoked by many techniques including meditation, progressive muscle relaxation, autogenic training, and yoga.^12^ The RR is characterized by a set of physiologic changes that include decreased oxygen consumption,^14^ decreased carbon dioxide elimination, decreased respiratory rate,^10,15–17^ increased brain cortical thickness,^18^ increased low-frequency heart rate oscillations,^19,20^ increased exhaled nitric oxide,^14^ and specific changes in gene expression.^21,22^

A growing body of evidence supports an association between MBI and BP reduction, in addition to decreasing levels of anxiety and depression in patients with HTN.^9,23–25^ Meta-analyses of randomized controlled trials indicate that MBI including relaxation and stress reduction therapies may reduce systolic blood pressure (SBP) in essential HTN, although no clear-cut conclusion could be drawn.^26^ In 2008, the author's group demonstrated the efficacy of the RR in controlling HTN without metabolic, hemodynamic, or central nervous system side effects,^27^ enabling the elimination of at least one antihypertensive medication in 32% of subjects receiving RR training.^27^

In the past, it has been assumed that regardless of how BP is controlled, the protective response will be similar.^28^ However, recent comparative clinical trials have concluded that the mechanism by which BP is controlled is an important variable in determining the clinical outcome of the patient cohort and may vary from patient to patient.^29^ One factor that may differentiate the clinical response in patients treated with different therapeutic modalities is the effect on inflammatory markers, whose expression levels are impacted by altered gene expression and, the authors contend, may be targeted by RR training.^30^ Transcriptional profiling of *qigong*^31^ and *t'ai chi*^32^ practitioners and the authors' two studies of RR-practicing healthy subjects^21,22^ revealed a distinct and consistent set of anti-inflammatory gene expression changes in mind–body practitioners.^33^ Altered immune functions and leukocyte blood gene expression changes have been linked to anxiety disorders and various other social stresses.^34,35^

To identify biological pathways and mechanisms of action underlying RR-mediated BP reduction, a study of RR training in patients with stage 1 essential HTN was conducted. The authors' effort focused on comparing the transcriptome changes in patients who exhibited reduced BP after RR training (BP responders) with nonresponders whose BP was not significantly influenced by the RR training (BP nonresponders).

## Materials and Methods

### Study design

This prospective single-arm trial involved enrolling 58 patients diagnosed with stage 1 essential HTN who were either not receiving antihypertensive drug therapy or who were receiving antihypertensives but willing to be tapered off. Patients were recruited from the Massachusetts General Hospital (MGH) Hypertension Clinics and from MGH primary care clinics. After enrollment and tapering (if necessary) under medical supervision for a minimum of 4 weeks, study eligibility was confirmed at three weekly run-in visits performed by the MGH Hypertension Clinic. Eligible patients received an RR-based intervention. The study protocol was approved by the Partners Human Research Committee, the Institutional Review Board of the MGH, and Partners HealthCare. Informed consent was obtained from all patients. The study was carried out in accordance with the approved guidelines and was registered on ClinicalTrials.gov (Identifier: NCT01263743).

### Run-in visits

Advancement to the intervention phase required patients to (1) meet criteria for stage 1 HTN, defined as SBP between 140 and 159 mm Hg and diastolic blood pressure (DBP) between 90 and 104 mm Hg on average at the three weekly run-in visits, (2) be off antihypertensive medications, and (3) have no prior RR-elicitation experience. During each run-in visit, after patients had been seated at rest for 5 min, three BP measurements were taken by a study nurse, with at least 2 min between measurements. If either SBP or DBP measurements varied by >8 mm Hg, additional measurements were taken until values were within this range. The average values of SBP and DBP from the three run-in visits were used as the baseline BP value. Patients whose baseline values met the stage 1 HTN criteria already listed were considered to have stable HTN and were advanced to the intervention phase.

### Preintervention biopsych testing

After the successful completion of the run-in visits, patients returned to the Clinical Research Center (CRC) at MGH for baseline biopsych testing. Premenopausal female patients attended baseline visits between days 1 and 11 or between days 22 and 28 of their menstrual cycle, and a urine pregnancy test was performed to ensure minimal hormonal confounds. Patients who had been taking antihypertensive medication before study enrollment had been completely off antihypertensive medications for at least 5 weeks. Fasting blood was drawn by nursing staff into RNA PAXgene tubes (Qiagen) for genomic analysis. After the blood draw, patients completed the Perceived Stress Scale (PSS), the Beck Depression Inventory-II (BDI-II), and the Beck Anxiety Inventory (BAI).

### RR training

The enrolled patients received training in techniques to elicit the RR, which included eight weekly individual training sessions from an experienced RR trainer. During the weekly sessions, patients were guided through an RR elicitation routine, including diaphragmatic breathing, body scan, mantra repetition, and mindfulness meditation, while passively ignoring intrusive thoughts. A 20-min audio CD that guided listeners through this same sequence was given to the patients to be listened to at home once a day.^21^ As per Galvin et al.,^36^ daily at-home RR practice was logged by participants.

### Postintervention biopsych testing and BP determination

Immediately after the final RR training session, patients returned to the CRC for endpoint biopsych testing, which involved the same blood draw and questionnaires as the baseline visit. At a separate visit to the MGH Hypertension Clinic, final endpoint BP was recorded by a study nurse, typically within 2 weeks after the final RR training. However, a few patients' BP was measured between 2 and 4 weeks after the final RR training.

### RNA sample collection, isolation, and profiling

Blood samples for transcriptome analysis were collected into PAXgene tubes (Qiagen) for stabilizing RNA. Total RNA was isolated from the peripheral blood mononuclear cells (PBMCs) as described previously.^21^

For transcriptional profiling, the Affymetrix human genome GeneChip HT HG-U133+ PM 24-Array Plate was used. The peripheral blood transcriptome profile was assessed on hypertensive patients with paired pre- and postintervention samples. Microarray analysis was conducted by the BIDMC Genomics, Proteomics, Bioinformatics and Systems Biology Center at the Beth Israel Deaconess Medical Center according to the standard Affymetrix protocol using the high-throughput Affymetrix GeneTitan system. Overview of study design is shown in Supplementary Figure S1 (Supplementary Data are available online at www.liebertpub.com/acm).

### Data analysis

An overview of the transcriptome data analysis plan is shown in Supplementary Figure S1.

#### Definition of BP responders

After completion of the 8-week RR intervention, patients who demonstrated (1) a significant reduction in both SBP (at least 10 mm Hg decrease) and DBP (at least 5 mm Hg decrease) and (2) BP below AHA stage 1 HTN clinical limits (SBP <140 mm Hg and DBP <90 mm Hg) were classified as “responders” (*n* = 13). Individuals who completed the study, but did not meet these thresholds, were classified as “nonresponders” (*n* = 11).

#### Baseline characteristics and self-report outcomes

Descriptive statistics were used to describe baseline subject characteristics. Regarding changes in outcome variables (SBP/DBP readings; PSS, BAI, and BDI-II scores), paired samples *t*-tests were used to compare pre-and postscores for responders and nonresponders. If post data were missing for a given scale, data were entered using last observation carried forward. Effect size [mean difference/standard deviation (SD) of difference] was also calculated for self-report measures. In all cases, statistical tests were two-tailed. Descriptive statistics were completed using Stata version 11.0 (Stata Corp, College Station, TX) and pre- to postanalyses were completed using SPSS Statistics 17.0; *p*-values of <0.05 were considered statistically significant.

#### Microarray quality control analysis

The technical quality of hybridized microarrays was assessed using Affymetrix standard quality control measures including perfect match (PM) mean, PM residual mean, 3′–5′ ratios for β-actin, and GAPDH (glyceraldehyde-3-phosphate dehydrogenase) as well as spike-in control transcripts. Reproducibility of the samples was checked by using chip-to-chip correlation and signal-to-noise ratio methods for replicate arrays using arrayQualityMetrics, a Bioconductor package in R.^37^ Based on these quality control analyses, microarrays for two pairs of pre- and post-RR patients (one responder and one nonresponder) were excluded from further analysis. All the 22 high-quality arrays (12 responders and 10 nonresponders) were included for unsupervised and supervised bioinformatics analysis.

#### Normalization, unsupervised, and supervised analysis of transcriptome data

The high-quality arrays were normalized using the robust multichip average (RMA) method in R using Bioconductor. RMA performs background correction, quantile normalization, and summarization on multiple oligonucleotides per transcript using the median polish algorithm.^38^ To identify outliers and batch effects, unsupervised analysis using principal component analysis (PCA) and hierarchical clustering analysis (HCA) was performed. The PCA projects multivariate data objects onto a lower dimensional space while retaining as much of the original variance as possible.^39,40^ The HCA was performed using Pearson correlation matrices with complete-linkage method. Batch effects, due to multiple transcriptome runs, were removed using ComBat, an empirical Bayes method.^41^ The differentially expressed genes among pre- versus postintervention samples were defined using a random-variance *t*-test. The random-variance *t*-test is an improvement over the standard separate *t*-test as it permits sharing information among genes about within-class variation without assuming that all genes have the same variance.^42^ Genes were considered statistically significant if their *p*-value was <0.05. *P*-values for significance were computed based on 1000 random permutations, at a significance level of each univariate test of 0.05 using BRB-ArrayTools.^43^

### Identification of BP response-associated genes

To determine whether the drop in BP after the RR intervention was linked to specific antihypertensive changes in gene expression and pathophysiologic pathways, transcriptome perturbations in BP responders and nonresponders were compared, by comparing RNA isolated from PBMCs at baseline (pre-RR) to RNA isolated after 8 weeks of RR practice (post-RR) and identifying the differences between responders and nonresponders. The transcriptome comparison was performed using both individual genes and gene sets/pathways to identify genes and pathways altered by the RR intervention.

#### Gene ontology analysis

To identify over-represented gene ontology (GO) categories in differentially expressed genes, the Biological Processes and Molecular Functions Enrichment Analysis available from the Database for Annotation, Visualization, and Integrated Discovery (DAVID) was used.^44^ DAVID is an online implementation of the EASE software that produces a list of over-represented categories. A *p*-value is calculated for each biological category on the basis of enrichments using Fisher's exact test. The GO categories with *p*-values <0.05 and for at least three genes were considered significant.

#### Pathway and interactive network analysis

To more precisely understand the complex interactions between the differentially expressed genes and the key differences between responders and nonresponders, and to identify the key focus hub genes (genes with the largest number of connections and anticipated to be those that ensure the stability and integrity of the network), interactive network analysis of the relaxation response hypertension (RR HTN) Responder Signature of 172 genes using the Ingenuity Pathway Analysis software package (IPA 8.0; Qiagen) was performed. This analysis generates an interaction network based on known functional interactions such as protein–protein interactions, protein activation, or gene regulation interactions, and demonstrates the interconnectivity between genes. IPA was used to identify the pathways and interaction networks significantly affected by genes that are altered after RR practice in responders only. A detailed description of IPA is available at the Ingenuity Systems' web site. IPA calculates a *p*-value for each pathway according to the fit of the user's data to the IPA database using a one-tailed Fisher exact test. The pathways with *p*-values <0.05 were considered significantly affected.

#### Regulatory module analysis

The regulatory module analysis was used to identify the cascade of upstream transcriptional regulators that can explain observed gene expression changes. The regulatory module analysis was performed using the upstream regulator analysis module available in IPA platform.^45^ Regulatory analysis helps in identifying significantly activated or inhibited transcriptional regulators on the basis of upregulation or downregulation of its target genes. The significance of transcriptional regulators activation/inhibition was determined using one-tailed Fisher's exact test.

#### Gene set enrichment analysis

In addition to individual gene analysis, gene set enrichment analysis (GSEA) was implemented to determine whether *a priori* defined sets of genes showed statistically significant, concordant differences between pre- and postintervention samples.^46^ GSEA can be more powerful than single-gene methods for studying the effects of interventions such as the RR in which many genes each make subtle contributions. The gene sets with nominal *p*-value <5% and a false discovery rate (FDR) <25% after 500 random permutations were considered significantly altered. GSEA was performed on the basis of 1007 canonical pathways obtained from the MSig2.0 database. Furthermore, GSEA on the basis of 345 blood transcript modules (BTMs) representing B and T cell, natural killer (NK) cell, neutrophil, and macrophage populations to determine their enrichment in RR HTN responders and nonresponders was performed.^47^

In addition to BTM analysis, CIBERSORT analysis on gene expression data from RR HTN responders and nonresponders was performed to estimate the abundance of different immune cells as components of overall PBMC gene expression.^48^ The CIBERSORT algorithm characterizes the cell-type composition of complex tissues/biological fluids from their gene expression profiles. The gene signatures of 22 distinct immune cell types (LM22) for this analysis were used.^48^ The signatures were developed from gene expression profiles of each immune cell type using Affymetrix and Illumina platforms.

#### Leading edge analysis

The significantly enriched gene sets (or pathways) identified from GSEA already described may have significant overlap in terms of core-enriched genes that are potentially linked to enrichment of multiple gene sets. Thus, the enriched gene sets were merged into functional modules on the basis of overlap of significantly enriched genes. The “leading edge” analysis considers genes shared across the gene sets as most strongly associated with the phenotype or key effects of intervention. Therefore, leading edge analysis to cluster significant gene sets and identify common genes that accounted for the core enrichment signal was used. The core set of genes identified from leading set analysis was considered for further validation.

#### Correlation of genes with changes in BP

To evaluate whether the relative changes of SBP and DBP in the patients before and after RR training correlate with gene expression changes in responders, correlation analysis using linear regression was conducted. The correlation analysis was performed by comparing change in BP and gene expression after 8 weeks of RR training and practice. For the purpose of this analysis, the pre- to postdifference in mean arterial pressure (MAP; equivalent to DBP+⅓[SBP-BDP]) was used. Correlation analysis was performed separately for BP responders and nonresponders to select the genes that have a significant correlation (*p* < 0.05) in BP responders but not in nonresponders. The correlation analysis was performed for 1821 and 1280 differentially expressed transcripts for BP responders and nonresponders, respectively, which were identified from the individual gene-based analysis.

## Results

### Baseline characteristics

**FIG. 1.**
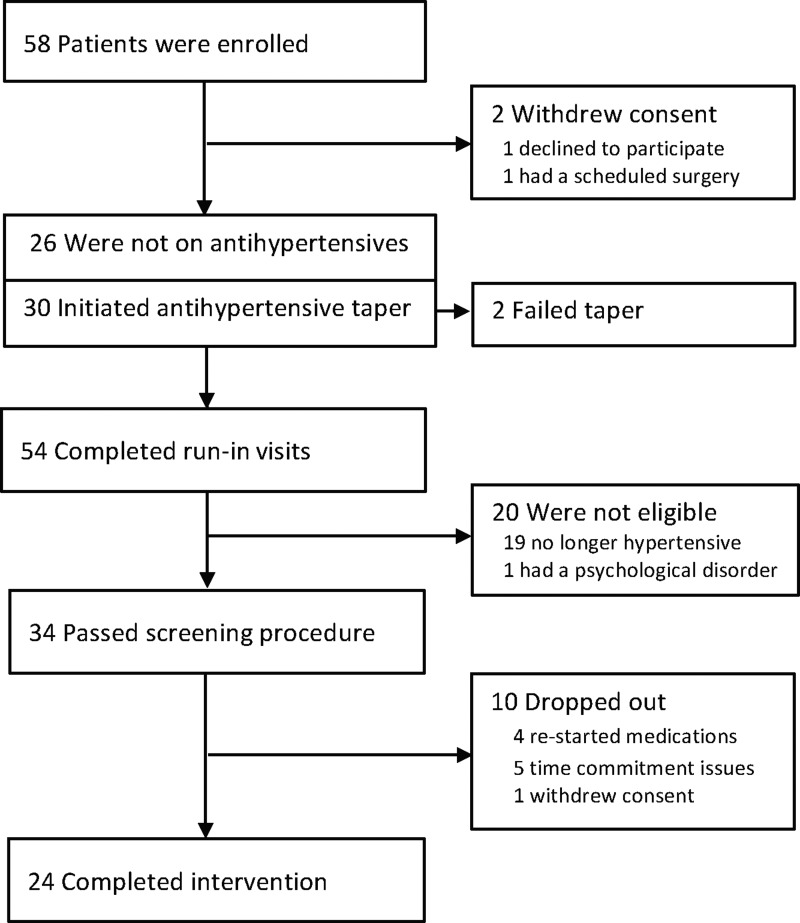
Flow of patients through the trial. CONSORT diagram of patient flow through the trial. A total of 58 patients signed consent and were enrolled, 34 passed all screening procedures, and 24 patients completed the intervention.

**FIG. 2.**
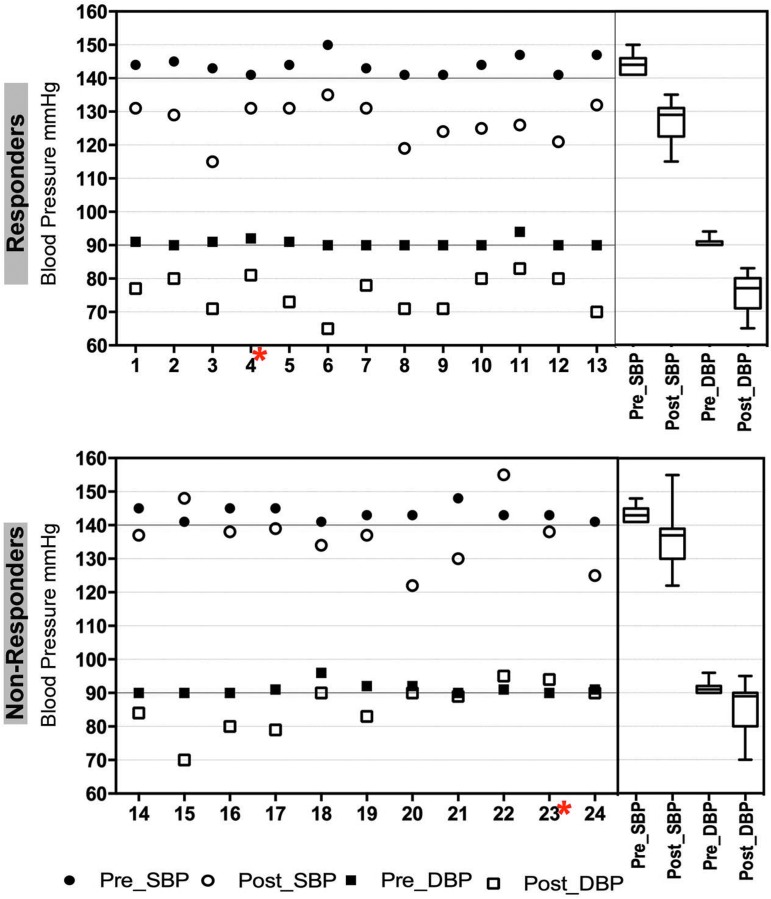
Significant SBP and DBP reduction in hypertensive patients is associated with short-term RR training (8 weeks). Specific pre- and post-RR BP values (mm Hg) for SBP and DBP for responders (*upper graph*) and nonresponders (*lower graph*) are shown for each patient. Box and whisker plots on the right summarize the median SBP and DBP levels in responders and nonresponders. Patients excluded from genomic analysis due to low data quality are marked with a *red asterisk*. BP, blood pressure; DBP, diastolic blood pressure; RR, relaxation response; SBP, systolic blood pressure.

Figure 1 shows the flow of patients through the trial. A total of 58 patients with stage 1 essential HTN who were either off antihypertensive medications or willing to be tapered were enrolled (i.e., signed informed consent); 2 of these withdrew consent before medication taper and run-in and 2 initiated tapering visits but withdrew (i.e., failed tapering). Out of the 54 patients who completed run-in visits, 34 passed all screening procedures, 19 were ineligible because they no longer met criteria for stage 1 HTN after the run-in period, and 1 was ineligible because of a psychological disorder. Ten enrolled patients dropped out during the intervention, leaving 24 completers. Table 1 describes the baseline characteristics of the 24 patients who completed the 8 weeks of RR training, demonstrating no significant differences between responders and nonresponders on any of these variables. At baseline, average SBP was 143.8 mm Hg, ranging from 141 to 150 mm Hg; the average DBP was 90.9 mm Hg, ranging from 90 to 96 mm Hg (Fig. 2; Table 1).

**Table 1.** Baseline Characteristics of Blood Pressure Responders and Nonresponders

	*Total (*n* = 24)*	*Responders (*n* = 13)*	*Nonresponders (*n* = 11)*	p
Demographics, mean (SD)				
Age (years)	55.6 (10.2)	57.7 (9.8)	53.2 (10.7)	0.29
HTN duration (years)	5.4 (5.4)	4.8 (5.7)	6.1 (5.1)	0.55
Demographics, *n* (%)				
Female sex	17 (71)	9 (69)	8 (73)	0.72
Married	12 (50)	5 (38)	7 (64)	0.23
White^a^	20 (83)	10 (77)	10 (91)	0.38
Hispanic	1 (4)	0 (0)	1 (9)	0.29
Employed	18 (75)	9 (69)	9 (82)	0.50
Completed college	15 (63)	7 (54)	8 (73)	0.36
Baseline measures, mean (SD)				
SBP (mm Hg)	143.7 (2.5)	143.9 (2.8)	143.5 (2.2)	0.65
DBP (mm Hg)	90.9 (1.5)	90.7 (1.2)	91.2 (1.8)	0.43
PSS	14.8 (7.8)	12.9 (8.3)	16.9 (7.1)	0.23
BDI-II	5.6 (5.6)	4.1 (4.8)	7.4 (6.1)	0.15
BAI	5.3 (4.9)	4.8 (3.8)	5.9 (6.1)	0.62

All values in *n* (%), unless stated otherwise.

^a^ For race, the four nonwhite patients included three African American and one Asian patient.

BAI, Beck Anxiety Inventory; BDI-II, Beck Depression Inventory-II; DBP, diastolic blood pressure; HTN, hypertension; PSS, Perceived Stress Scale; SBP, systolic blood pressure; SD, standard deviation.

### Blood pressure

Figure 2 and Table 2 show the baseline pre-RR data for the 24 patients who completed the RR training and the changes in SBP and DBP for each patient from baseline to the post-RR time point after 8 weeks of RR training. Overall, 8 weeks of RR training decreased SBP by 15.4 mm Hg (*p* < 0.001) and DBP by 10.6 mm Hg (*p* < 0.001). Although these decreases in SBP and DBP were statistically significant, not all patients achieved an improvement in BP that was *clinically* significant. Using stringent predefined parameters for BP reduction, patients were separated into BP responders and nonresponders. This differentiation was a key step for further downstream analysis of BP reduction associated with the RR intervention and the link to changes in the transcriptome, because it allowed a between-group comparison. Based on the stringent criteria defined in the Materials and Methods section, 13 out of 24 patients (54%) were classified as BP responders and 11 patients (46%) as BP nonresponders (Fig. 2; Table 2). Some of the BP nonresponders had SBP but not DBP reduction or vice versa but did not match the authors' stringent *clinical relevance* criteria. These patients, within the BP nonresponder class, may be considered “partial” responders. The authors' rigorous clinically meaningful definition of BP response *a priori* maximizes the chance of finding genomic changes associated with true BP response. Figure 2 shows the individual (left side) and group (right side) differences in BP changes between BP responders and BP nonresponders.

**Table 2.** Pre–Post Blood Pressure Values of Responders and Nonresponders

	Pre			Post		
	n	*M*	*SD*	n	*M*	*SD*
Responders						
SBP	13	143.9	2.8	13	126.9	5.9
DBP	13	90.7	1.2	13	75.4	5.5
Nonresponders						
SBP	11	143.5	2.2	11	136.6	9.4
DBP	11	91.2	1.8	11	85.8	7.5
Total sample						
SBP	24	143.7	2.5	24	131.4	9.0
DBP	24	90.9	1.5	24	80.2	8.3

DBP, diastolic blood pressure; M, mean; SBP, systolic blood pressure; SD, standard deviation.

### Self-report outcomes

After completion of the 8-week RR intervention, BP responders and BP nonresponders, taken together, reported improvements on all psychological self-report measures. There were statistically significant improvements on BAI scores (5.21 vs. 3.17; *t* = 2.39; *p* = 0.026) and PSS scores (pre: 14.29 vs. post: 11.50; *t* = 2.43; *p* = 0.02); mean BDI-II scores dropped from 5.58 to 4.13, but this difference was not statistically significant (*t* = 1.90; *p* = 0.069). Overall effect sizes of change on psychological measures as measured by Cohen's D were moderate (PSS: *d* = 0.50; BAI: *d* = 0.49; BDI-II: *d* = 0.39).

When BP responders and BP nonresponders were analyzed separately, a slightly different picture emerged. On the BAI, responders had statistically significant improvements (mean ± SD; pre: 4.83 ± 3.79; post: 2.83 ± 2.52, *t* = 2.23, *p* = 0.047) but nonresponders (pre: 5.9 ± 6.08; post: 3.4 ± 2.54, *t* = 1.401, *p* = 0.195) did not. On the BDI, responders showed marginally significant improvement (pre: 4.08 ± 4.79; post: 2.62 ± 3.78, *t* = 2.13, *p* = 0.054) compared with nonresponders (pre: 7.36 ± 6.1; post: 5.91 ± 5.82, *t* = 0.967, *p* = 0.356), who did not have significant improvement. On the PSS, neither responders (pre: 12.9 ±8.25; post: 10.5 ± 7.38, *t* = 1.75, *p* = 0.107) nor nonresponders (pre: 16.91 ± 7.15; post: 13.45 ± 7.13, *t* = 1.68, *p* = 0.124) had significantly decreased scores when considered separately.

### Transcriptional changes after 8 weeks of RR training in HTN patients: RR HTN signature

**FIG. 3.**
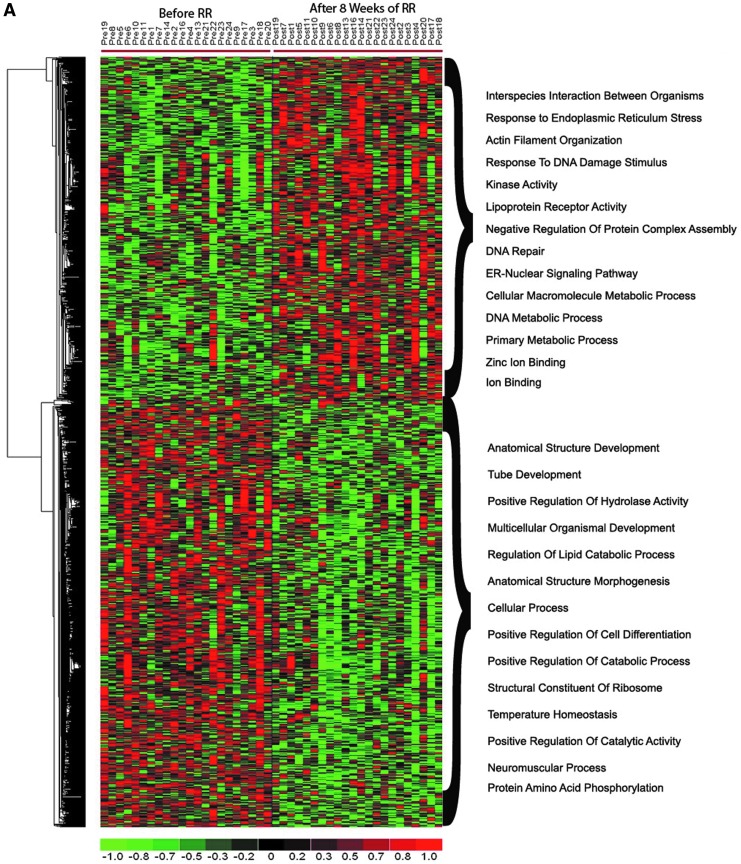

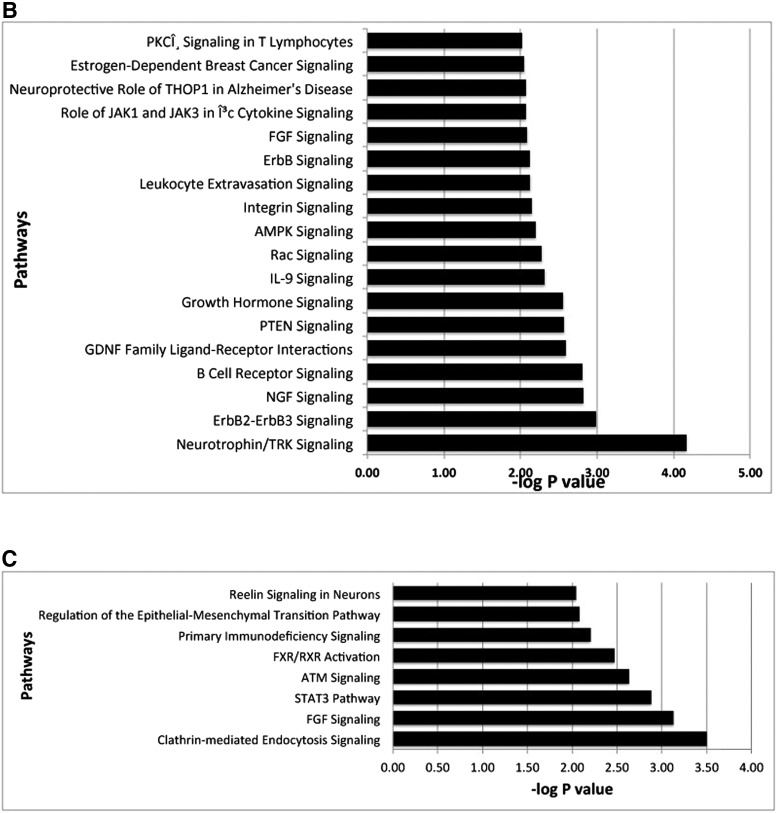
Transcriptome changes and significantly affected pathways in hypertension patients after 8 weeks of RR intervention. (**A**) A heatmap of significantly differentially expressed genes after 8 weeks of RR training. Relative gene expression differences pre- to post-RR intervention for significantly differentially expressed genes are shown with a pseudocolor scale (-1 to 1), with red denoting higher gene expression and green denoting lower expression after intervention. (**B**) Pathway enrichment analysis of significantly upregulated genes, and (**C**) pathway enrichment analysis of significantly downregulated genes. Selected top pathways of differentially upregulated (**B**) and downregulated (**C**) genes are shown. The y-axis represents pathways and the x-axis the - log transformed p-value (e.g., a value of 2 represents a p-value of 0.01). ER, endoplasmic reticulum; IL, interleukin; RR, relaxation response.

To identify the transcripts modulated after 8 weeks of RR training in HTN patients (i.e., the RR HTN gene expression signature), the transcriptional profile of all patients from baseline to 8 weeks of RR practice was compared. Only 22 of the 24 eligible patients were included in this and the following analyses: the microarrays for the remaining two patients (labeled in Fig. 2 with a red asterisk) did not pass the authors' quality control measures due to poor array quality caused by low quality of the RNA samples. This analysis identified 1387 transcripts as significantly differentially expressed (*p* < 0.05) between baseline and 8 weeks of RR practice (Fig. 3A; Supplementary Table S1). GO analysis (see Materials and Methods section) showed significant upregulation of genes linked to endoplasmic reticulum stress, DNA repair, and kinase activity after RR practice (Fig. 3A) and downregulation of genes linked to lipid metabolism, cellular differentiation, and neuromuscular processes (Fig. 3A). To understand the biological meaning of RR-associated changes, pathway enrichment analysis was also performed. Pathway enrichment analysis showed a significant over-representation of upregulated genes in pathways linked to cell proliferation and survival (e.g., integrin signaling, ErbB signaling, PTEN signaling, NGF signaling, FGF signaling, RAC signaling, and TRK signaling), T and B lymphocyte signaling, and energy production (e.g., AMPK signaling) (Fig. 3B). Significant enrichment of downregulated genes was linked to STAT3, FXR/RXR activation, ATM, and Reelin signaling pathways (Fig. 3C).

### Identification of BP response-associated genes in HTN patients using individual gene-based analysis

**FIG. 4.**
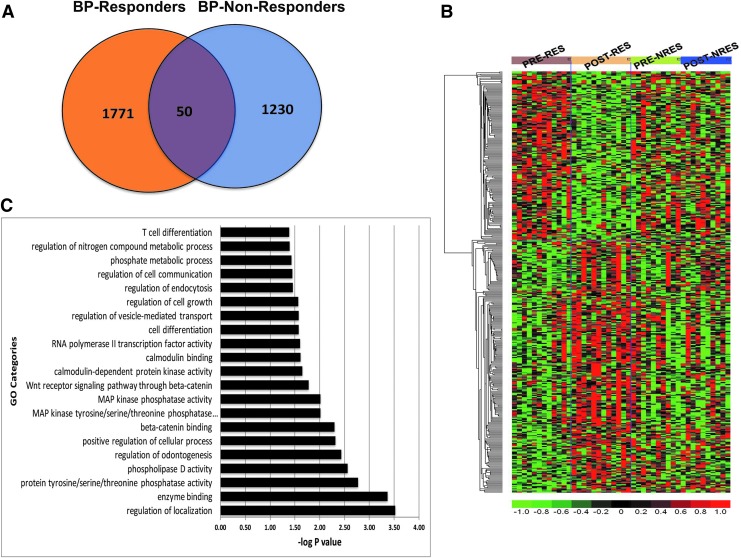
Transcriptome changes distinguishing BP responders from nonresponders after 8 weeks of RR intervention. **(A)** Venn diagram depicting common genes between BP responders and nonresponders and transcripts selectively changing in responders or nonresponders only. Fifty genes were commonly altered between BP responders and nonresponders. **(B)** Heatmap of the *top* 388 genes that are significantly altered in BP responders (RES) but not in nonresponders (NRES). **(C)** GO analysis of genes that are exclusively differentially expressed in BP responders. The *y*-axis represents GO categories and the *x*-axis the -log transformed *p*-value (e.g., a value of 2 represents a *p*-value of 0.01). BP, blood pressure; GO, gene ontology; MAP, mean arterial pressure; RR, relaxation response.

In both BP responders and nonresponders, differentially expressed genes were identified by comparing expression profiles of pre versus post-RR patients using a permuted paired univariate *t*-test. The differential expression analysis (post-RR vs. pre-RR) identified 1821 and 1280 differentially expressed transcripts for the 12 responders and 10 nonresponders, respectively (permuted *p* < 0.05). Venn diagram analysis identified 50 transcripts altered after RR training that overlapped between responders and nonresponders (Fig. 4A). Most importantly, 1771 transcripts were uniquely impacted after RR training in BP responders, indicating that these transcripts may be directly associated with the BP lowering ability of RR (Fig. 4A). Hierarchical clustering using the 388 top BP responder-specific genes based on more stringent selection (*p* < 0.05 and fold change >1.2; Supplementary Table S2) shows striking segregation between pre- and post-RR in BP responders but not in BP nonresponders, validating the hypothesis that 8 weeks of RR practice is associated with differential expression of a distinct set of genes in PBMCs specific to BP responders (Fig. 4B).

To gain deeper insight into the molecular mechanisms of RR-associated changes in BP responders only, GO enrichment analysis on the 388 responder-specific differentially expressed genes was first performed. This analysis identified a significant effect of RR on multiple biological processes, including “Phospholipase D activity,” “Beta-catenin binding,” “MAP kinase phosphatase activity,” “Cellular growth and proliferation,” and “Calmodulin-dependent protein kinase activity” (Fig. 4C).

### Identification of BP response-associated genes in HTN patients highly correlated with BP change

**FIG. 5.**
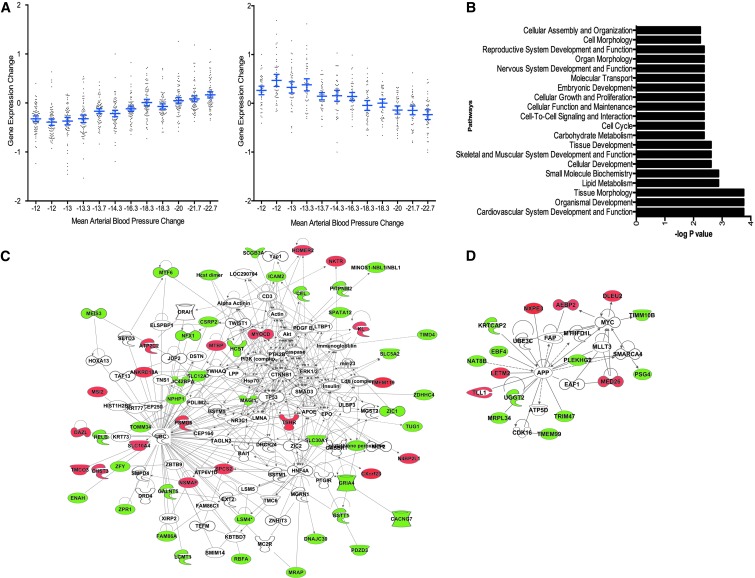
Identification of gene expression changes and pathways significantly correlating with BP changes after 8 weeks of RR intervention. **(A)** Correlation analysis for differentially expressed transcripts in BP responders and nonresponders for which changes in the pre-MAP to post-MAP (equivalent to DBP +1/3[SBP-DBP]) and changes in gene expression were significantly correlated (*p* < 0.05). Out of these 121 genes, 46 and 75 genes showed significant positive (*right graph*) and negative (*left graph*) correlation, respectively, between gene expression change (*x*-axis) and MAP change (*y*-axis). **(B)** Pathway analysis of genes depicting significant correlation with BP changes. The *y*-axis represents pathways and the *x*-axis the -log transformed *p*-value (e.g., a value of 2 represents a *p*-value of 0.01). **(C)** Interactive network of BP change correlated genes that is enriched with metabolism (lipid, nucleic acid, and drug metabolism) and connective tissue disorder-related genes. **(D)** Interactive network of BP change correlated genes that is enriched with cardiovascular disease-related genes. For both **(C, D)**, network nodes are colored on the basis of correlation between gene expression and BP (i.e., positively correlated = *red*, negatively correlated = *green*, colorless nodes = no correlation). BP, blood pressure; DBP, diastolic blood pressure; MAP, mean arterial pressure; RR, relaxation response; SBP, systolic blood pressure.

Correlation analysis (see the Materials and Methods section) identified 121 genes for which change in MAP and change in gene expression were significantly correlated (*p* < 0.05; Supplementary Table S3). Out of these 121 genes, 46 showed significant positive (decreasing gene expression correlates with decreasing BP) and 75 showed negative (increasing gene expression correlates with decreasing BP) correlation between gene expression and MAP (Fig. 5A). Insight into the biological functions of genes showing correlation with BP reduction was obtained by functional enrichment analysis. Functional categories with *p* < 0.05 were considered significant. Cardiovascular system development and function is the top affected functional category. Various other functional categories linked to metabolism (e.g., lipid and carbohydrate metabolism) and cell cycle (e.g., cellular growth and proliferation, cell cycle, and cell morphology) were also significantly enriched (Fig. 5B).

Scale-free interactive network analysis was performed on these 121 BP correlated genes to analyze the functional networks and to identify the key nodes that might be relaying reduction in BP. The nodes of networks were colored on the basis of correlation between gene expression and BP (i.e., positively correlated = red, negatively correlated = green). This analysis identified BP-correlated genes that are enriched in networks related to metabolism (lipid, nucleic acid, and drug metabolism) and connective tissue disorder (Fig. 5C) as well as cardiovascular disease (Fig. 5D).

### GSEA reveals that RR is associated with significantly upregulated glucose transport and suppressed inflammation pathways in BP responders

**FIG. 6.**
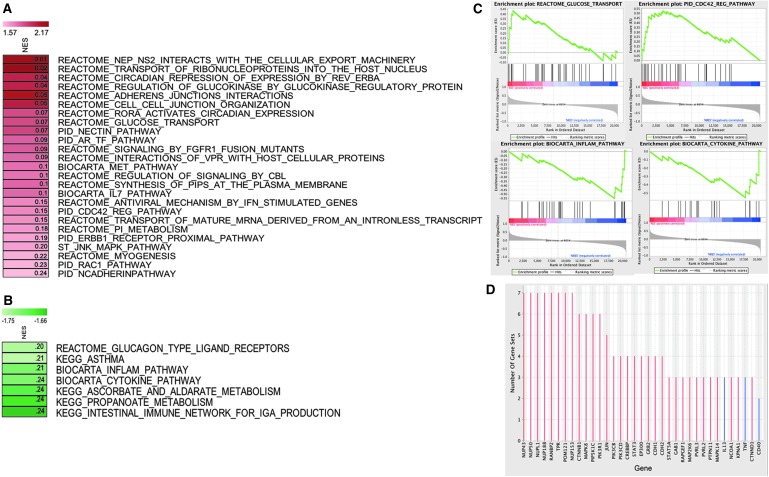
Gene set enrichment analysis identifies pathways specifically impacted in BP responders. **(A)** Pathways that are significantly upregulated in BP responders. **(B)** Pathways that are significantly downregulated in BP responders. The significantly affected pathways were identified on the basis of normalized *p*-value (<0.05) and false discovery rate (<0.25; values for each pathway in the NES *shaded boxes*). The upregulated and downregulated pathways are shown with *red* and *green shaded boxes*, respectively (*red* = positive NES, *green* = negative NES). **(C)** Enrichment maps of selected significantly upregulated (glucose transport and PID regulation of CDC42 pathway) and downregulated (biocarta inflammation pathway and biocarta cytokine pathway) pathways. **(D)** Leading edge analysis identification of potential key genes that are highly abundant in significantly affected pathways. Bar graph depicts the number of gene sets in which each gene is altered after the RR practice. *Red* and *blue* bars represent genes that are significantly upregulated and downregulated after the RR practice, respectively. BP, blood pressure; NES, normalized enrichment score; RR, relaxation response.

GSEA (see the Materials and Methods section) identified 32 pathways (25 upregulated, 7 downregulated) that were significantly (*p* < 0.05, FDR <0.25) modulated in BP responders compared with nonresponders (Fig. 6). Significantly upregulated pathways include, among others, glucose metabolism (glucose transport, regulation of glucokinase by glucokinase regulatory protein), cardiovascular system development (e.g., N-cadherin, myogenesis, RAC1, and NECTIN), cell–cell interactions (cell–cell junction organization and adherens junctions interactions), circadian rhythm (circadian repression of expression by REV-ERBA, RORA activates circadian expression), and the MET pathway (Fig. 6A). The significantly downregulated pathways include pathways related to immune function (e.g., cytokine pathway and inflammation pathway) and to metabolism (e.g., ascorbate and aldarate metabolism and propanoate metabolism) (Fig. 6B). Enrichment maps of a subset of these significantly upregulated and downregulated pathways are depicted in Figure 6C.

To identify the individual genes that are key targets in BP responders, leading edge analysis (Fig. 6D) was performed. Leading edge analysis identifies the set of genes that is consistently and significantly dysregulated in multiple pathways. The top genes from leading edge analysis are thought to govern the phenotype, and are thus the critical genes for formulating a hypothesis about the genetic mechanism underlying the potential impact of RR on BP. This analysis identified multiple nuclear transport, cell cycle, protein kinase cascade, and insulin receptor signaling pathway-related genes (*NUP43*, *NUP50*, *NUPL1*, *NUP188*, *RANBP2*, *TPR*, *POM121*, *NUP153*, *CTNNB1*, *PIP5K1C*, *PIK3R1*, *MAPK8*, *PIK3CB*, *PIK3CD*, and *JUN*) as top genes that may be critically involved in BP response after an RR intervention in HTN patients (Fig. 6D).

GSEA based on BTMs depicted significant enrichment (*p* < 0.05, FDR <0.25) of the “Inositol phosphate metabolism” module in BP responders compared with nonresponders (Supplementary Table S4). Further repeated analysis with relaxed significance cutoff (*p* < 0.09) showed that BP nonresponders have significant upregulation of multiple immune and inflammation-related cellular modules including “T cell surface signature,” “B cell signature,” “dendritic cell signature through NFKB activation,” and “lymphocyte homing and migration” as compared with responders (Supplementary Table S4). These results indicate that the nonresponder group exhibits a pattern biased toward activated inflammation and higher immune cell enrichment as than responders.

To further decipher the contribution of different cell types to the PBMC expression profile, an *in silico* analysis was performed to determine the abundance of different cell types from the PBMC gene expression data using the CIBERSORT algorithm.^48^ After estimating the abundance of cell types in each sample, comparative analysis showed that BP responders have significantly higher abundance of M2 macrophages than nonresponders (*p* = 0.02 and fold change >3.5). Other immune cells do not show any statistically significant difference between responders and nonresponders. It is interesting that BP responders appear to have a significantly higher number of anti-inflammatory M2 macrophages, suggesting an association between BP response and anti-inflammation. Combining the results from the CIBERSORT and BTM analysis, it was predicted that the BP response has a significant association with inflammation levels.

### Identification of RR HTN Responder Signature by integrating results of GSEA and individual gene-based analysis

To further focus on genes whose perturbation may lead to BP reduction in HTN patients after RR training, a BP responder signature was generated. To accomplish this task, the top differentially expressed genes that were significantly correlated with BP change in BP responders (Fig. 5) were combined with genes from pathways that are significantly differentially regulated in BP responders compared with nonresponders in multiple gene sets (Fig. 6). The final RR HTN Responder Signature consists of 172 genes, out of which 84 are downregulated and 88 are upregulated (Supplementary Table S5).

### Upstream regulators of genes differentially expressed in HTN responders to RR therapy

**FIG. 7.**
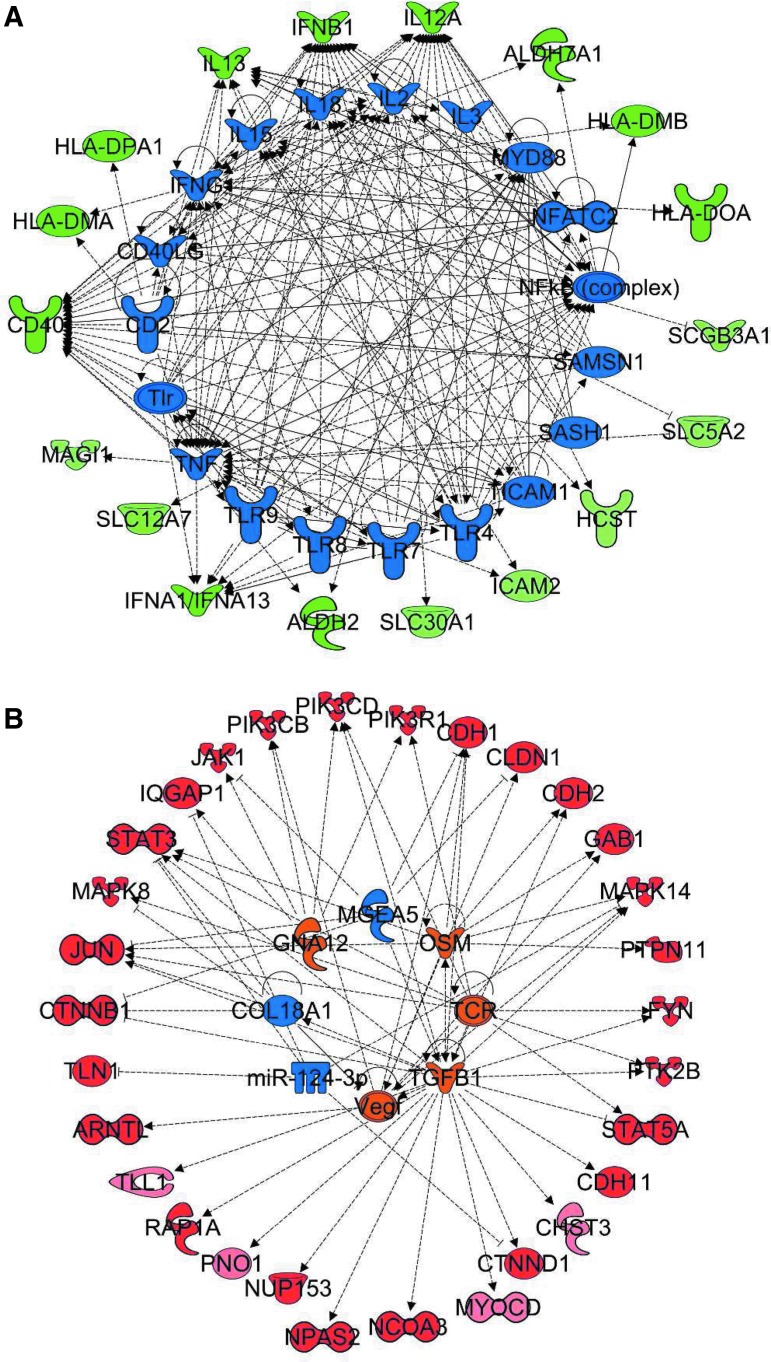
Upstream regulator analysis of genes significantly linked to BP response in HTN patients. Upstream regulator analysis of genes significantly linked to BP response in HTN patients. This analysis helps to identify upstream regulators that are predicted to be significantly activated or inhibited upstream of the gene expression alterations after the RR training. *Top* upstream regulators identified from analysis of downregulated **(A)** and upregulated **(B)** genes from the RR HTN Responder Signature. The upstream regulators that are predicted to be activated and inhibited are *orange* and *blue*, respectively. The downstream targets of each regulators are shown with *red* (genes upregulated by RR) and *green* (genes downregulated by RR). BP, blood pressure; HTN, hypertension; RR, relaxation response.

To assess whether a specific set of upstream regulators may explain how the RR is associated with the expression of genes within the RR HTN Responder Signature in association with BP reduction, upstream regulator enrichment analysis using the Upstream Regulator Tool of IPA was conducted. This tool enables the prediction of which upstream regulators are likely involved in altering expression of a set of genes and predicts whether these regulators are inhibited or activated. Analysis of the 84 downregulated genes of the RR HTN Responder Signature predicted inhibition of multiple immune system molecules [e.g., CD2, several members of the Toll-like receptor (TLR) family (*TLR4*, *TLR7*, *TLR8*, *TLR9*), NFATC2, nuclear factor-κB (*NF*-*κB*) (complex), *IFNG*, *CD40LG*, tumor necrosis factor (TNF), interleukin (IL)-15, IL-18, IL-2, IL-3] to be responsible for reduced expression of these downstream targets, suggesting that the RR blunts inflammatory and immune processes through reduced activity of this set of regulators, most interestingly NF-κB (Fig. 7A). Similarly, regulator analysis of the 88 upregulated genes from the RR HTN Responder Signature revealed that activation of several cell growth and immune system molecules (e.g., transforming growth factor-β1, T cell receptor, vascular endothelial growth factor, and oncostatin M) and inhibition of extracellular matrix-related molecules (e.g., *COL18A1* and *Mir124-3p*) (Fig. 7B) may be involved in increased expression of these genes.

The regulator network indicates that the RR may affect inflammation in a bimodal manner by upregulating anti-inflammatory pathways [i.e., glucocorticoid (GC) receptor signaling] and downregulating NF-κB and TNF pathways. Interactive network analysis of RR-affected genes in responders identified NF-κB and related downstream targets as top critical focus hubs. Most genes upstream and downstream of NF-κB were decreased in responders providing the first insight that NF-κB might be crucial in mediating RR effects on HTN. NF-κB might be playing a major role in achieving beneficial effects of RR. NF-κB was also identified as a key focus molecule in previous studies^49^ focused on identifying the genomic determinants of RR in healthy subjects as well as in the study of the effects of RR on irritable bowel syndrome and inflammatory bowel disease patients.^49^

## Discussion

This trial of RR training for unmedicated patients with stage 1 HTN demonstrates that BP is effectively lowered in over half of all patients after an 8-week RR training and is associated with improvements in psychological variables and specific changes in gene expression. Although these data are compelling and suggest a direct effect of the RR on BP, in this uncontrolled study, it cannot be ruled out that other factors may have caused the drop in BP. The 8-week RR intervention used here was, however, associated with substantial improvement of BP, with over half of patients meeting stringent response criteria requiring improvement in both SBP (>10 mm Hg) and DBP (>5 mm Hg), despite the presence of only mild (stage 1) HTN. The stringency of the response criteria makes it clinically relevant to patient care, and ensures that responder status is unlikely to be affected by regression to the mean.

Although guidelines for goals in BP reduction in the elderly remain controversial and the JNC7 goal for diabetics and chronic kidney disease patients is reduction to <130/80 mm Hg, most clinicians in the HTN community are using the traditional target of <140/90 mm Hg as the goal for patients with uncomplicated HTN, the patient population used in this study. The improvements in BP observed in this study are consistent with changes seen with antihypertensive medications^50–52^ and are consistent with related work showing the impact of MBIs on BP in a variety of populations.^27,53–55^ To the authors' knowledge, this is the first study to test such an intervention for a population of unmedicated adults with carefully documented persistent HTN, and the first study to identify genomic determinants associated with the impact of an MBI on HTN. Results from this study provide new insights into how integrative medicine, especially mind–body approaches, influences BP control at the molecular level.

On psychological self-report measures, BP responders and BP nonresponders, considered together, had significant improvement in anxiety symptoms (as measured by the BAI) and trended toward improvement in depression symptoms (as measured by the BDI-II). Considered separately, only BP responders had significant or near-significant improvements in anxiety and depression, respectively. This indicates that the positive effect on BP paralleled improvements in psychological symptoms, suggesting (but not proving, in the absence of a control group) that BP response was likely the result of RR training. Given that patients were recruited on the basis of BP and not on the basis of stress levels (with initial scores on the PSS below population means), it is interesting that there were small but significant improvements in perceived stress in the patients analyzed as a group. When BP responders and BP nonresponders were considered separately, however, the improvements were not significant—an unsurprising result given the likely floor effect and the small sample size.

Although HTN has been attributed primarily to regulation through the renin–angiotensin system in the kidney and perturbations of the vasculature and central nervous system, increasing evidence demonstrates a significant contribution of the immune system, especially the innate immune system—inflammatory cells and cytokines such as TNF-α, IL-17, and IL-6—in association with the sympathetic nervous system.^56,57^ Indeed, the vasoactive peptide angiotensin II has been known for some time to activate NF-κB, which then provides intracellular signaling for angiotensin II-induced target organ effects, including effects in vascular smooth muscle cells that contribute to HTN.^58^ Focusing on peripheral blood for studying the effect of the RR on HTN-linked gene expression may not be ideal, but it is an easily available biological source for determining the pathophysiologic pathways of BP control; in addition, angiotensin II is known to activate NF-κB not only in vascular smooth muscle cells but also in human monocytes,^59^ which of course contribute to gene expression in peripheral blood. The results clearly define pathways directly or indirectly impacted by the RR-associated drop in BP; still, whether these gene expression changes are causal or surrogates for BP regulation remains to be determined. As discussed in more detail hereunder, however, key gene expression changes and pathways identified in this study have previously been linked to HTN, which suggests that the findings here bear further testing.

Those findings show that specific changes in PBMC gene expression were significantly associated with BP changes after RR practice. A robust systems-level transcriptome RR HTN Responder Signature was generated using multiple bioinformatics approaches (see Supplementary Figure S1 for the overall scheme), including a traditional individual gene-based approach, as well as advanced systems biology and GSEA, resulting in the identification of a significant number of BP responder-specific genes and signaling pathways that have previously been linked to HTN, as well as those not yet associated with BP control. A total of 121 differentially expressed genes were identified that significantly correlate with BP changes. An Integrated RR HTN Responder Signature of 172 genes was subsequently generated by combining genes that are differentially expressed and significantly correlated with BP improvement among RR-responsive HTN patients (Fig. 5) with genes derived from the leading edge analysis (Fig. 6). Systems biology analysis of this Integrated RR HTN Responder Signature provided new insights into the biological pathways, upstream regulators, and key focus hub genes that are predicted to be the bottlenecks for relaying the upstream RR-elicited signals to downstream targets.

One of the most consistent pathways emerging from these analyses was inflammation/immune response, including downstream targets of NF-κB, selectively influenced in BP responders after RR training. Several lines of evidence from the data analysis support the apparent key function of NF-κB in mediating at least part of the beneficial effect of the RR on reduction of BP. First, leading edge analysis identified, among the most consistently responder-specific upregulated genes, primarily those involved in nuclear transport (*NUP43*, *NUP50*, *NUPL1*, *NUP188*, *RANBP2*, *TPR*, *POM121*, and *NUP153*). Altered nuclear transport plays a key role in oxidative stress-mediated effects and impacts nuclear transport and redistribution of NF-κB, a key regulatory mechanism in NF-κB activation.

Furthermore, upregulation of circadian rhythm pathways (circadian repression of expression by REV-ERBA, RORA activates circadian expression) in BP responders was identified by GSEA as significantly affected subsequent to the RR practice. Since circadian regulation of BP is well established, and dysregulation of circadian rhythm can cause HTN, one model emerging from the authors' studies is that the RR may help regulate circadian rhythm, which may at least partially contribute to BP control.^60–62^ This may be highly relevant with regard to the potential role of NF-κB as a mediator of RR effects, since REV-ERBA, a critical regulator of circadian rhythm, induces NF-κB activation in vascular smooth muscle cells, leading to expression of proinflammatory cytokines.^63^ In contrast, REV-ERBA blocks RORA-mediated repression of cytokine expression. The authors, thus, speculate that RR enhances repression of REV-ERBA expression, resulting in enhanced RORA activation, decreased NF-κB activity, and decreased cytokine production.

Cytokines have been demonstrated to play critical roles in endothelial and smooth muscle cell responses, and GSEA indicates significant downregulation of inflammatory and cytokine pathways associated with the RR in BP responders. Various studies support the notion that inflammation contributes to endothelial dysfunction and the development of HTN.^64^ Systemic low-grade inflammation, as reflected by increased C-reactive protein (CRP) and cytokine levels, is thought to precede HTN.^65^ Immune cell function, particularly involving T cells and macrophages, has been directly implicated in the development of HTN in animal models, possibly by causing endothelial dysfunction as a consequence of cytokine-mediated alterations in the rates of synthesis and degradation of nitric oxide and other vasoconstrictors or vasodilators.

The authors' integrated model of RR-elicited HTN responder-specific gene expression alterations provides further insights into the mechanisms of RR-associated BP reduction that are not obvious from the individual analyses viewed in isolation. Importantly, this Integrated RR HTN Responder Signature of 172 genes further supports the involvement of NF-κB in mediating RR-associated improvements in BP, as revealed by the most significant enrichment of pathways among the downregulated genes being linked to immune function and inflammation (e.g., NF-κB signaling, crosstalk between dendritic cells and NK cells, T helper cell differentiation, IL-12 signaling and production in macrophages, TREM1 signaling, cytokine communication between immune cells, antigen presentation pathway, TLR signaling, and communication between innate and adaptive immune cells). Counter-intuitively, when analyzing the upregulated genes, several immune function pathways (IL-2 signaling and B cell receptor signaling) were identified as enriched as well. This result suggests selective alteration of immune functions in BP responders after RR training, namely a potential switch in T helper cell differentiation from proinflammatory TH1 (reduced IL-12 production) to TH2 (enhanced IL-2 signaling), reduced innate immune response (TLR signaling and communication between innate and adaptive immune cells), reduced inflammation (NF-κB signaling), and enhanced B cell function (B cell receptor signaling).

The authors' observed association of reduced innate immune activity (TLR signaling) with reduced BP after the RR intervention is further strengthened by the upstream regulator analysis, which predicts reduced activity of TLRs (*TLR4*, *TLR7*, *TLR8*, and *TLR9*) based on the reduced expression of several downstream target genes (*ICAM2*, *SLC30A1*, *ALDH2*, *IFNA1*, *IFNA13*, *SLC12A7*, and *CD40*). Activation of TLRs in vascular or renal damage associated with HTN has been described in preeclampsia, renal disease, spontaneously hypertensive rats, and angiotensin II-induced HTN. That TLRs may play an important role in HTN is supported by the finding that inhibiting TLR with neutralizing antibodies resulted in BP reduction and downregulation of NF-κB, ICAM1, chemokines, and cytokines.^66,67^ It was previously reported that eliciting the RR through a variety of interventions downregulates NF-κB activity, and in this study, this effect has been repeated.

In addition to the downregulation of NF-κB, the upregulation of anti-inflammatory processes (e.g., GC receptor signaling) is observed.^21,22^ It is of interest that cognitive behavioral stress management has been reported to decrease the activity of the NF-κB family of transcription factors and increase activity of the GC receptor in early stage breast cancer patients.^68^ Furthermore, investigation of transcriptome changes in dementia family caregivers, randomized to simple yogic meditation or music listening, demonstrated that yoga, which elicits the RR, reverses the pattern of increased NF-κB-dependent transcription of proinflammatory cytokines and replicates the finding of reduced NF-κB activity after randomization to an RR-inducing meditation group.^69,70^ Transcriptome changes associated with *t'ai chi* and cognitive behavior therapy in insomnia patients also showed significant downregulation of inflammatory markers, including NF-κB and CRP.^71^ Chronically stressed family caregivers of brain cancer patients demonstrate deactivation of GC response elements and increased activation of NF-κB. Stress may lead to functional GC receptor resistance to GCs in monocytes, which then leads to proinflammatory transcription control pathway activation.^72^ In summary, different MBIs support the model that NF-κB-mediated reduction of inflammation is a potential mechanism associated with RR-mediated control of BP in HTN patients.

The following are some study limitations. These limitations include small sample size, a short-term intervention lasting only 8 weeks, absence of data on lifestyle behavior during the trial changes (e.g., low-salt diet, weight loss, and exercise), and an inability to control for the effects of socioeconomic status. Moreover, this study had no comparison group for the RR intervention. Since patients were not randomized to a placebo or no-treatment control, it is difficult to distinguish between regression to the mean and a bona fide BP clinical response to the RR intervention. Thus, it cannot be definitively concluded that the effects on BP and gene expression are specific to or caused by the RR. Whether BP responders to the RR differ with regard to the molecular mechanisms of BP reduction in responders to medications, health education, or other interventions cannot be determined in this study; however, what can be concluded from this study is that BP responders have distinct transcriptome alterations not seen in BP nonresponders that may or may not be specific for RR intervention, but clearly correlate with reduction in BP. Future studies are planned to address whether the RR induces unique transcriptome changes or impacts similar pathways as antihypertensive drugs or health education. The authors' previous work suggests, in fact, that different interventions may act by different mechanisms. For example, health education and the RR both appear to reduce BP in a significant percentage of patients, but only the RR reduces BP medication requirements as well, suggesting mechanistic differences between the RR and health education interventions.^27^

The limitations are balanced by several notable strengths. First, of particular note, is the rigorous definition of BP response to therapy. The authors' definition of BP response is the most rigorous of possible definitions and adds to the validity of this analysis. In addition, multiple complementary approaches for identification of genes and pathways that are affected by the RR in HTN subjects have been used, increasing the reliability of the conclusions. Finally, the comprehensive systems biology analysis links RR-associated BP reduction with genes known to be linked to BP regulation, providing a sound biological rationale for these findings.

## Conclusions

The treatment of HTN is based upon lifestyle modification as the foundation of improved BP control and reduced cardiovascular events. Traditionally, HTN patients are treated with pharmacologic therapy, but not all patients respond to drug therapy and many experience treatment-limiting adverse experiences. In these patients, alternative strategies are invaluable. In this study, it is demonstrated that the RR can successfully contribute to the reduction of the BP of untreated HTN patients. Importantly, the transcriptome changes associated with this fall in BP are consistent with the changes in hemodynamic parameters and inflammatory markers that one would anticipate, and hope to observe, in successfully treated patients.

In summary, the results suggest that the RR reduces BP, at least partially, by altering expression of genes in a select set of biological pathways, most prominently involving NF-κB as a key regulatory molecule. Supporting this finding are important changes in the genomic signature of individuals who, after the RR intervention, had clinically meaningful changes in BP as compared with those who did not. The study design was not able to prove a causal link between BP reduction and changes in gene expression, but these data are suggestive and provide new hypotheses that can be tested in future studies. Importantly, this unified model of RR function provides support for a scenario wherein selective alteration of inflammatory processes and immune functions, likely linked to oxidative stress and imbalance of circadian rhythm, may contribute to BP reduction.

## Supplementary Material

Supplemental data
