# Supplementary material for: Specific Transcriptome Changes Associated with Blood Pressure Reduction in Hypertensive Patients After Relaxation Response Training
Source: J Altern Complement Med. 2018 May 1;24(5):486–504. doi: 10.1089/acm.2017.0053 (PMC5961875; doi:10.1089/acm.2017.0053)
Supplement: Supplemental data [file Supp_Data.pdf]

**Supplementary Information**

# Specific transcriptome changes associated with blood pressure reduction in hypertensive patients following Relaxation Response training

Manoj K. Bhasin[1,4,5#], John W. Denninger[1,2#], Jeff C. Huffman[2], Marie G. Joseph[5], Halsey Niles[1], Emma Chad-Friedman[1,2], Roberta Goldman[1], Beverly Buczynski-Kelley[6], Barbara A. Mahoney[6], Gregory L. Fricchione[1,2], Jeffery A. Dusek[7], Herbert Benson[1,3†], Randall M. Zusman[6†], Towia A. Libermann[1,4, 5†*]

1- Benson-Henry Institute for Mind Body Medicine at Massachusetts General Hospital, Boston, MA 02114, USA

2- Department of Psychiatry, Massachusetts General Hospital, Harvard Medical School, Boston, MA 02114, USA

3- Department of Medicine, Massachusetts General Hospital, Harvard Medical School, Boston, MA 02114, USA

4- Department of Medicine, Division of Interdisciplinary Medicine and Biotechnology, Beth Israel Deaconess Medical Center, Harvard Medical School, Boston, MA 02115, USA

5- BIDMC Genomics, Proteomics, Bioinformatics and Systems Biology Center, Beth Israel Deaconess Medical Center, Boston, MA 02115, USA

6- Department of Medicine, Corrigan-Minehan Heart Center, Cardiology Division, Section on Hypertension, Massachusetts General Hospital and Harvard Medical School, Boston, MA

7- Penny George Institute for Health and Healing, Allina Health, Minneapolis, MN 55407, USA

[#]Contributed equally and should be considered first authors.

†Contributed equally and should be considered senior authors.

[+]Dr. Dusek is now at the Kripalu Center for Yoga and Health, Stockbridge MA 02162 USA

**Supplementary Figures and Tables:**

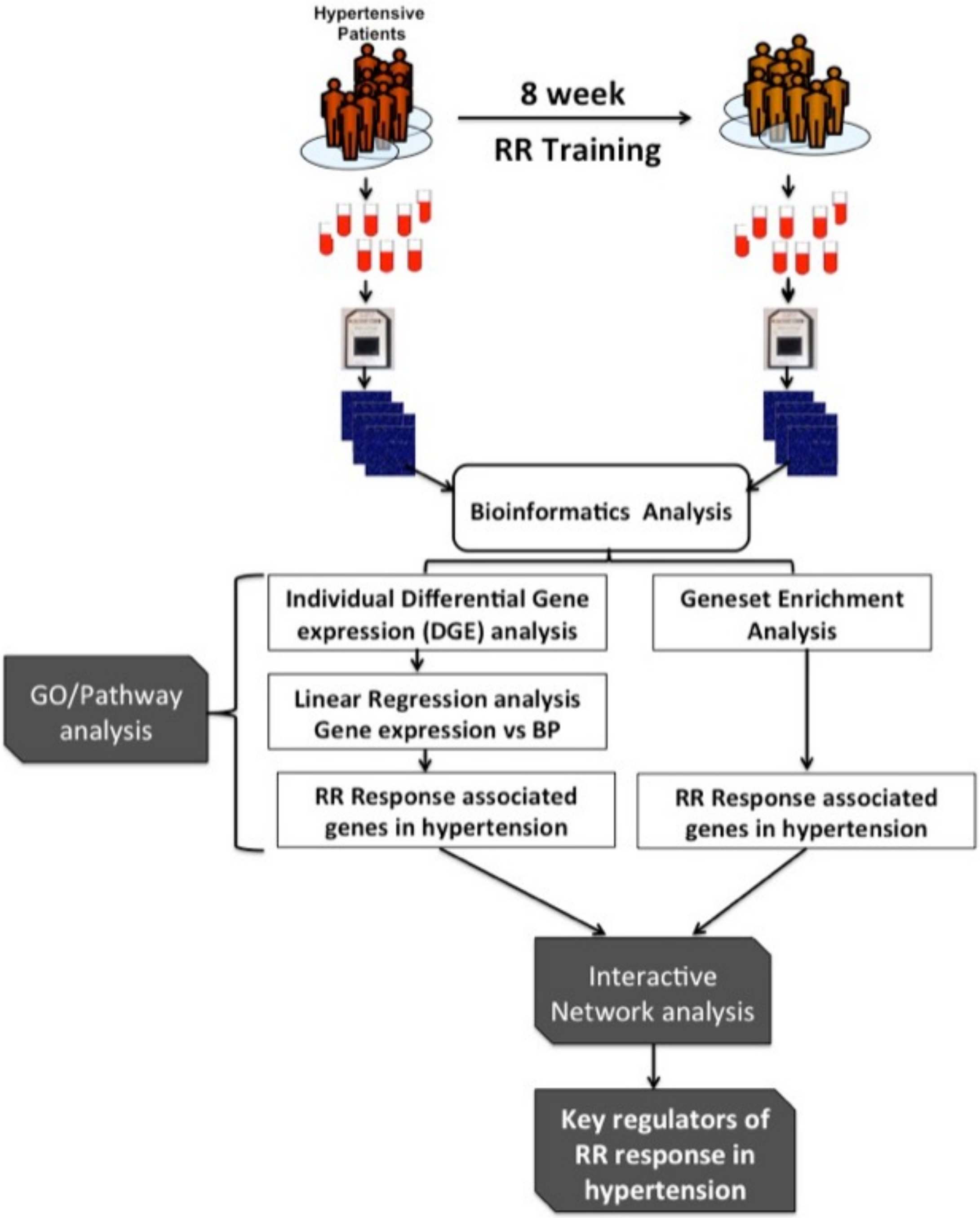


**Fig S1: Schematic view of study design to identify relaxation response induced clinical, psychological and transcriptome changes in hypertensive patients.** The transcriptome profiling was performed on peripheral blood mononuclear cells (PBMCs) collected

before first session of RR (Pre-RR) and after last session of RR (Post-RR). The transcriptome profiling was performed using HT_U133 Plus PM arrays plate containing >47,000 transcripts. The transcriptome data was analyzed using multiple bioinformatics approaches to identify genes and pathways associated with blood pressure changes after RR training. Further systems biology analysis was performed on gene expression changes in responders to identify key molecules that might be responsible for delivering beneficial effects of RR in hypertensive patients.

**Table S1:** Transcriptional changes induced after 8 weeks of RR in Hypertensive Patients.

**Table S1: Transcriptional changes induced after 8 weeks of RR in Hypertensive Patients.**

| ProbeSet | Symbol | Name | EntrezID | Fold Change | Parametric p-value | Permutation p-value |
|---|---|---|---|---|---|---|
| 221491_x_at | | | | 1.52 | 0.0268286 | 0.0438 |
| 215242_at | | | | 1.41 | 0.0006741 | 0.0033 |
| 206676_at | CEACAM8 | carcinoembryonic antigen-related cell adhesion molecule 8 | 1088 | 1.4 | 0.0173096 | 0.0289 |
| 233371_at | ABCC13 | ATP-binding cassette, sub-family C (CFTR/MRP), member 13, pseudogene | 150000 | 1.31 | 0.0268515 | 0.05 |
| 232465_at | | | | 1.3 | 0.006167 | 0.0141 |
| 209649_at | STAM2 | signal transducing adaptor molecule (SH3 domain and ITAM motif) 2 | 10254 | 1.28 | 0.0156863 | 0.0272 |
| 210367_s_at | PTGES | prostaglandin E synthase | 9536 | 1.26 | 0.0161487 | 0.0306 |
| 225388_at | TSPAN5 | tetraspanin 5 | 10098 | 1.26 | 0.0273207 | 0.0458 |
| 1552737_s_a | WWP2 | WW domain containing E3 ubiquitin protein ligase 2 | 11060 | 1.26 | 0.0299361 | 0.0496 |
| 201292_at | TOP2A | topoisomerase (DNA) II alpha 170kDa | 7153 | 1.25 | 0.0004153 | 6.00E-04 |
| 224225_s_at | ETV7 | ets variant 7 | 51513 | 1.25 | 0.0161417 | 0.0256 |
| 203757_s_at | CEACAM6 | carcinoembryonic antigen-related cell adhesion molecule 6 (non-specific cross reacting antigen) | 4680 | 1.25 | 0.0175259 | 0.0309 |
| 236821_at | | | | 1.24 | 0.0041928 | 0.0078 |
| 229249_at | | | | 1.24 | 0.0081791 | 0.0149 |
| 231236_at | ZFP57 | ZFP57 zinc finger protein | 346171 | 1.24 | 0.0278299 | 0.0243 |
| 225366_at | PGM2 | phosphoglucomutase 2 | 55276 | 1.23 | 0.029226 | 0.0442 |
| 235250_at | FLCN | folliculin | 201163 | 1.23 | 0.0310079 | 0.0494 |
| 232156_at | | | | 1.22 | 0.0060491 | 0.0075 |
| 1555749_at | SF1 | splicing factor 1 | 7536 | 1.22 | 0.0169293 | 0.0262 |
| 207329_at | MMP8 | matrix metallopeptidase 8 (neutrophil collagenase) | 4317 | 1.22 | 0.0226949 | 0.0321 |
| 204501_at | NOV | nephroblastoma overexpressed | 4856 | 1.22 | 0.0195355 | 0.0324 |
| 233099_at | | | | 1.22 | 0.0185285 | 0.0337 |
| 1569206_at | TCP11L2 | t-complex 11, testis-specific-like 2 | 255394 | 1.22 | 0.0257561 | 0.0404 |
| 224428_s_at | CDCA7 | cell division cycle associated 7 | 83879 | 1.22 | 0.0312925 | 0.0479 |
| 236531_at | | | | 1.21 | 0.0035367 | 0.004 |
| 227777_at | | | | 1.21 | 0.0054612 | 0.0092 |
| 229889_at | FAM211A | family with sequence similarity 211, member A | 388341 | 1.21 | 0.0073747 | 0.0117 |
| 232421_at | SCARB1 | scavenger receptor class B, member 1 | 949 | 1.21 | 0.0085687 | 0.0158 |
| 224760_at | SP1 | Sp1 transcription factor | 6667 | 1.21 | 0.0147237 | 0.0247 |
| 202198_s_at | MTMR3 | myotubularin related protein 3 | 8897 | 1.21 | 0.0230759 | 0.0359 |
| 239944_at | | | | 1.2 | 0.0022804 | 0.0034 |
| 204686_at | IRS1 | insulin receptor substrate 1 | 3667 | 1.2 | 0.0055347 | 0.0077 |
| 229756_at | LOC1009965 | uncharacterized LOC100996537 | 100996537 | 1.2 | 0.0114279 | 0.0196 |
| 205594_at | ZNF652 | zinc finger protein 652 | 22834 | 1.2 | 0.0194163 | 0.0294 |
| 202094_at | BIRC5 | baculoviral IAP repeat containing 5 | 332 | 1.2 | 0.0270407 | 0.0395 |
| 220992_s_at | TRMT1L | tRNA methyltransferase 1 homolog (S. cerevisiae)-like | 81627 | 1.2 | 0.0304631 | 0.0458 |
| 240793_at | TTN | titin | 7273 | 1.2 | 0.0348587 | 0.049 |
| 1566037_at | DGCR7 | DiGeorge syndrome critical region gene 7 | 266621 | 1.19 | 0.0003517 | 4.00E-04 |
| 241262_at | | | | 1.19 | 0.0004576 | 7.00E-04 |
| 231519_at | | | | 1.19 | 0.0005711 | 0.0014 |
| 208474_at | CLDN6 | claudin 6 | 9074 | 1.19 | 0.0030132 | 0.005 |
| 209498_at | CEACAM1 | carcinoembryonic antigen-related cell adhesion molecule 1 (biliary glycoprotein) | 634 | 1.19 | 0.0103303 | 0.0157 |
| 242905_at | PNO1 | partner of NOB1 homolog (S. cerevisiae) | 56902 | 1.19 | 0.0111833 | 0.017 |
| 212686_at | PPM1H | protein phosphatase, Mg2+/Mn2+ dependent, 1H | 57460 | 1.19 | 0.0196399 | 0.0289 |
| 235400_at | FCRLA | Fc receptor-like A | 84824 | 1.19 | 0.0225534 | 0.029 |
| 232812_at | LOC401052 | uncharacterized LOC401052 | 401052 | 1.19 | 0.0233162 | 0.0317 |
| 228935_at | SLC4A8 | solute carrier family 4, sodium bicarbonate cotransporter, member 8 | 9498 | 1.19 | 0.0233587 | 0.0332 |
| 236297_at | | | | 1.19 | 0.0278186 | 0.0429 |
| 236566_at | | | | 1.19 | 0.0289814 | 0.0433 |
| 235515_at | SYNE4 | spectrin repeat containing, nuclear envelope family member 4 | 163183 | 1.19 | 0.0310583 | 0.0454 |
| 235456_at | | | | 1.19 | 0.0340476 | 0.0482 |
| 227055_at | METTL7B | methyltransferase like 7B | 196410 | 1.18 | 0.0002633 | 2.00E-04 |
| 233995_at | | | | 1.18 | 0.0031695 | 0.006 |
| 201549_x_at | KDM5B | lysine (K)-specific demethylase 5B | 10765 | 1.18 | 0.0068539 | 0.0121 |
| 238452_at | FCRLB | Fc receptor-like B | 127943 | 1.18 | 0.0137827 | 0.0176 |
| 220636_at | DNAI2 | dynein, axonemal, intermediate chain 2 | 64446 | 1.18 | 0.0101905 | 0.0177 |
| 1554242_a_a | COCH | coagulation factor C homolog, cochlin (Limulus polyphemus) | 1690 | 1.18 | 0.0141839 | 0.0214 |
| 240038_at | | | | 1.18 | 0.0156418 | 0.0241 |
| 204956_at | MTAP | methylthioadenosine phosphorylase | 4507 | 1.18 | 0.0225171 | 0.0308 |
| 1554710_at | KCNMB1 | potassium large conductance calcium-activated channel, subfamily M, beta member 1 | 3779 | 1.18 | 0.0229196 | 0.036 |
| 228425_at | LOC654433 | uncharacterized LOC654433 | 654433 | 1.18 | 0.0318407 | 0.037 |
| 231600_at | CLEC12B | C-type lectin domain family 12, member B | 387837 | 1.18 | 0.0281451 | 0.0393 |
| 240036_at | SEC14L1 | SEC14-like 1 (S. cerevisiae) | 6397 | 1.18 | 0.0249651 | 0.0396 |
| 242582_at | | | | 1.18 | 0.0255592 | 0.0402 |
| 1558592_at | | | | 1.18 | 0.0307833 | 0.0441 |
| 233236_at | TSPAN16 | tetraspanin 16 | 26526 | 1.18 | 0.0327819 | 0.0451 |
| 221669_s_at | ACAD8 | acyl-CoA dehydrogenase family, member 8 | 27034 | 1.17 | 0.0020514 | 0.0027 |
| 230516_at | MALSU1 | mitochondrial assembly of ribosomal large subunit 1 | 115416 | 1.17 | 0.0041063 | 0.006 |
| 235033_at | NPEPL1 | aminopeptidase-like 1 | 79716 | 1.17 | 0.0068911 | 0.0101 |
| 1554486_a_a | GFOD1 | glucose-fructose oxidoreductase domain containing 1 | 54438 | 1.17 | 0.0070697 | 0.011 |
| 243409_at | FOXL1 | forkhead box L1 | 2300 | 1.17 | 0.0131187 | 0.022 |
| 212719_at | PHLPP1 | PH domain and leucine rich repeat protein phosphatase 1 | 23239 | 1.17 | 0.0156137 | 0.0232 |
| 226825_s_at | TMEM165 | transmembrane protein 165 | 55858 | 1.17 | 0.0145161 | 0.0244 |
| 233824_at | | | | 1.17 | 0.0180381 | 0.0248 |
| 233238_s_at | | | | 1.17 | 0.0218034 | 0.0301 |
| 1554010_at | NDST1 | N-deacetylase/N-sulfotransferase (heparan glucosaminyl) 1 | 3340 | 1.17 | 0.0217551 | 0.0334 |
| 230175_s_at | DCBLD2 | discoidin, CUB and LCCL domain containing 2 | 131566 | 1.17 | 0.0244629 | 0.0354 |

| ProbeSet | Symbol | Name | EntrezID | Fold Change | Parametric p-value | Permutation p-value |
|---|---|---|---|---|---|---|
| 216411_s_at | | | | 1.17 | 0.0252502 | 0.0354 |
| 223393_s_at | TSHZ3 | teashirt zinc finger homeobox 3 | 57616 | 1.17 | 0.0287845 | 0.0369 |
| 242729_at | TTN-AS1 | TTN antisense RNA 1 | 100506866 | 1.17 | 0.0247469 | 0.0378 |
| 236036_at | LOC1005063 | uncharacterized LOC100506343 | 100506343 | 1.17 | 0.0268441 | 0.0397 |
| 205451_at | FOXO4 | forkhead box O4 | 4303 | 1.17 | 0.0307618 | 0.0439 |
| 1564936_at | | | | 1.17 | 0.0344131 | 0.0488 |
| 226974_at | NEDD4L | neural precursor cell expressed, developmentally down-regulated 4-like, E3 ubiquitin protein ligase | 23327 | 1.16 | 0.0008338 | 8.00E-04 |
| 222261_at | KIAA1609 | KIAA1609 | 57707 | 1.16 | 0.0051594 | 0.0066 |
| 1559053_at | | | | 1.16 | 0.0049684 | 0.0068 |
| 219928_s_at | CABYR | calcium binding tyrosine-(Y)-phosphorylation regulated | 26256 | 1.16 | 0.006324 | 0.0075 |
| 231557_at | | | | 1.16 | 0.0087127 | 0.0086 |
| 217381_s_at | TARP | TCR gamma alternate reading frame protein | 445347 | 1.16 | 0.00656 | 0.0087 |
| 1552334_at | TRIOBP | TRIO and F-actin binding protein | 11078 | 1.16 | 0.0067186 | 0.0091 |
| 1554126_at | MSRB3 | methionine sulfoxide reductase B3 | 253827 | 1.16 | 0.0109535 | 0.0123 |
| 214674_at | USP19 | ubiquitin specific peptidase 19 | 10869 | 1.16 | 0.0096673 | 0.0124 |
| 236211_at | RAB40B | RAB40B, member RAS oncogene family | 10966 | 1.16 | 0.0101986 | 0.0134 |
| 210571_s_at | CMAHP | cytidine monophospho-N-acetylneuraminic acid hydroxylase, pseudogene | 8418 | 1.16 | 0.0105132 | 0.0148 |
| 204604_at | CDK14 | cyclin-dependent kinase 14 | 5218 | 1.16 | 0.0121163 | 0.017 |
| 213357_at | GTF2H5 | general transcription factor IIH, polypeptide 5 | 404672 | 1.16 | 0.0156005 | 0.0184 |
| 231962_at | AP4B1 | adaptor-related protein complex 4, beta 1 subunit | 10717 | 1.16 | 0.0132749 | 0.0195 |
| 1558371_a_at | | | | 1.16 | 0.021939 | 0.0207 |
| 243185_at | | | | 1.16 | 0.0149502 | 0.0215 |
| 217044_s_at | PLEKHG3 | pleckstrin homology domain containing, family G (with RhoGef domain) member 3 | 26030 | 1.16 | 0.0173469 | 0.0241 |
| 224043_s_at | UPB1 | ureidopropionase, beta | 51733 | 1.16 | 0.0173958 | 0.0247 |
| 226325_at | ADSSL1 | adenylosuccinate synthase like 1 | 122622 | 1.16 | 0.0178914 | 0.026 |
| 213038_at | RNF19B | ring finger protein 19B | 127544 | 1.16 | 0.0192429 | 0.0266 |
| 202254_at | SIPA1L1 | signal-induced proliferation-associated 1 like 1 | 26037 | 1.16 | 0.0209788 | 0.0344 |
| 228062_at | NAP1L5 | nucleosome assembly protein 1-like 5 | 266812 | 1.16 | 0.0262263 | 0.0349 |
| 242399_at | | | | 1.16 | 0.0272119 | 0.0351 |
| 203170_at | RRP8 | ribosomal RNA processing 8, methyltransferase, homolog (yeast) | 23378 | 1.16 | 0.0285173 | 0.0423 |
| 202345_s_at | FABP5 | fatty acid binding protein 5 (psoriasis-associated) | 2171 | 1.16 | 0.0302867 | 0.0428 |
| 228766_at | CD36 | CD36 molecule (thrombospondin receptor) | 948 | 1.16 | 0.0301358 | 0.0446 |
| 230832_at | RTF1 | Rtf1, Paf1/RNA polymerase II complex component, homolog (S. cerevisiae) | 23168 | 1.15 | 0.003821 | 0.0026 |
| 225687_at | FAM83D | family with sequence similarity 83, member D | 81610 | 1.15 | 0.0033355 | 0.0046 |
| 219497_s_at | BCL11A | B-cell CLL/lymphoma 11A (zinc finger protein) | 53335 | 1.15 | 0.003576 | 0.0046 |
| 232753_at | ZNF346 | zinc finger protein 346 | 23567 | 1.15 | 0.0087696 | 0.0072 |
| 237606_at | CD53 | CD53 molecule | 963 | 1.15 | 0.0099268 | 0.012 |
| 210187_at | FKBP1A | FK506 binding protein 1A, 12kDa | 2280 | 1.15 | 0.0099987 | 0.0122 |
| 215330_at | | | | 1.15 | 0.0108763 | 0.0152 |
| 214541_s_at | QKI | QKI, KH domain containing, RNA binding | 9444 | 1.15 | 0.0108941 | 0.0157 |
| 224773_at | NAV1 | neuron navigator 1 | 89796 | 1.15 | 0.0159959 | 0.0218 |
| 206167_s_at | ARHGAP6 | Rho GTPase activating protein 6 | 395 | 1.15 | 0.0179251 | 0.0242 |
| 226223_at | PAWR | PRKC, apoptosis, WT1, regulator | 5074 | 1.15 | 0.022528 | 0.0294 |
| 216976_s_at | RYK | receptor-like tyrosine kinase | 6259 | 1.15 | 0.0206219 | 0.0298 |
| 237130_at | | | | 1.15 | 0.021309 | 0.0298 |
| 1552632_a_a | ARSG | arylsulfatase G | 22901 | 1.15 | 0.0223586 | 0.0298 |
| 238789_at | KANK1 | KN motif and ankyrin repeat domains 1 | 23189 | 1.15 | 0.0205264 | 0.0304 |
| 211132_at | INTS3 | integrator complex subunit 3 | 65123 | 1.15 | 0.0215561 | 0.032 |
| 227915_at | ASB2 | ankyrin repeat and SOCS box containing 2 | 51676 | 1.15 | 0.0229253 | 0.0326 |
| 209191_at | TUBB6 | tubulin, beta 6 class V | 84617 | 1.15 | 0.0275112 | 0.0336 |
| 244549_at | | | | 1.15 | 0.0272078 | 0.0352 |
| 228497_at | SLC22A15 | solute carrier family 22, member 15 | 55356 | 1.15 | 0.0270125 | 0.0356 |
| 214639_s_at | HOXA1 | homeobox A1 | 3198 | 1.15 | 0.027742 | 0.0374 |
| 222309_at | | | | 1.15 | 0.0302428 | 0.0408 |
| 206369_s_at | PIK3CG | phosphatidylinositol-4,5-bisphosphate 3-kinase, catalytic subunit gamma | 5294 | 1.15 | 0.0329473 | 0.0434 |
| 220000_at | SIGLEC5 | sialic acid binding Ig-like lectin 5 | 8778 | 1.15 | 0.0326128 | 0.0436 |
| 209791_at | PADI2 | peptidyl arginine deiminase, type II | 11240 | 1.15 | 0.0327838 | 0.0448 |
| 231997_at | TBCEL | tubulin folding cofactor E-like | 219899 | 1.15 | 0.0330826 | 0.0477 |
| 224897_at | WDR26 | WD repeat domain 26 | 80232 | 1.15 | 0.0342719 | 0.0493 |
| 215657_at | SLC26A3 | solute carrier family 26, member 3 | 1811 | 1.14 | 0.0041926 | 0.0029 |
| 1555420_a_a | KLF7 | Kruppel-like factor 7 (ubiquitous) | 8609 | 1.14 | 0.0032693 | 0.0033 |
| 1556425_a_a | LOC284219 | uncharacterized LOC284219 | 284219 | 1.14 | 0.0036337 | 0.0037 |
| 238217_at | | | | 1.14 | 0.0045206 | 0.0045 |
| 236564_at | | | | 1.14 | 0.0046655 | 0.0048 |
| 1557155_a_at | | | | 1.14 | 0.0066074 | 0.0064 |
| 207249_s_at | SLC28A2 | solute carrier family 28 (sodium-coupled nucleoside transporter), member 2 | 9153 | 1.14 | 0.005722 | 0.0068 |
| 207341_at | PRTN3 | proteinase 3 | 5657 | 1.14 | 0.0070023 | 0.0076 |
| 231451_s_at | | | | 1.14 | 0.0090016 | 0.0082 |
| 204681_s_at | RAPGEF5 | Rap guanine nucleotide exchange factor (GEF) 5 | 9771 | 1.14 | 0.0073649 | 0.0086 |
| 1563565_at | INPP5B | inositol polyphosphate-5-phosphatase, 75kDa | 3633 | 1.14 | 0.0078759 | 0.0087 |
| 206436_at | MPPED1 | metallophosphoesterase domain containing 1 | 758 | 1.14 | 0.009485 | 0.0095 |
| 215980_s_at | IGHMBP2 | immunoglobulin mu binding protein 2 | 3508 | 1.14 | 0.0097467 | 0.0102 |
| 204423_at | MKLN1 | muskelin 1, intracellular mediator containing kelch motifs | 4289 | 1.14 | 0.0095925 | 0.0105 |
| 229082_at | CCDC125 | coiled-coil domain containing 125 | 202243 | 1.14 | 0.0099707 | 0.0113 |
| 209965_s_at | RAD51D | RAD51 homolog D (S. cerevisiae) | 5892 | 1.14 | 0.0111175 | 0.014 |
| 213005_s_at | KANK1 | KN motif and ankyrin repeat domains 1 | 23189 | 1.14 | 0.0131094 | 0.0162 |
| 217269_s_at | TMPRSS15 | transmembrane protease, serine 15 | 5651 | 1.14 | 0.0153233 | 0.0164 |
| 203328_x_at | IDE | insulin-degrading enzyme | 3416 | 1.14 | 0.0141502 | 0.0166 |

| ProbeSet | Symbol | Name | EntrezID | Fold Change | Parametric p-value | Permutation p-value |
|---|---|---|---|---|---|---|
| 225630_at | EEPD1 | endonuclease/exonuclease/phosphatase family domain containing 1 | 80820 | 1.14 | 0.0138346 | 0.018 |
| 228560_at | | | | 1.14 | 0.0153756 | 0.019 |
| 233003_at | | | | 1.14 | 0.0131618 | 0.0191 |
| 213358_at | SOGA2 | SOGA family member 2 | 23255 | 1.14 | 0.0148798 | 0.0196 |
| 235494_at | LSAMP | limbic system-associated membrane protein | 4045 | 1.14 | 0.0164445 | 0.0209 |
| 244087_at | | | | 1.14 | 0.0168653 | 0.0214 |
| 241409_at | | | | 1.14 | 0.0184119 | 0.0222 |
| 205632_s_at | PIP5K1B | phosphatidylinositol-4-phosphate 5-kinase, type I, beta | 8395 | 1.14 | 0.0172286 | 0.0231 |
| 227555_s_at | KHSRP | KH-type splicing regulatory protein | 8570 | 1.14 | 0.0215023 | 0.0241 |
| 214716_at | BMP2K | BMP2 inducible kinase | 55589 | 1.14 | 0.0216681 | 0.0271 |
| 211089_s_at | NEK3 | NIMA-related kinase 3 | 4752 | 1.14 | 0.0191252 | 0.0278 |
| 1563983_at | TPT1-AS1 | TPT1 antisense RNA 1 | 100190939 | 1.14 | 0.0227472 | 0.0279 |
| 221543_s_at | ERLIN2 | ER lipid raft associated 2 | 11160 | 1.14 | 0.0212678 | 0.0285 |
| 227300_at | TMEM119 | transmembrane protein 119 | 338773 | 1.14 | 0.0233494 | 0.0306 |
| 207042_at | E2F2 | E2F transcription factor 2 | 1870 | 1.14 | 0.0243776 | 0.0312 |
| 205444_at | ATP2A1 | ATPase, Ca++ transporting, cardiac muscle, fast twitch 1 | 487 | 1.14 | 0.0254943 | 0.0314 |
| 226081_at | LZIC | leucine zipper and CTNNBIP1 domain containing | 84328 | 1.14 | 0.0249933 | 0.0339 |
| 233880_at | RNF213 | ring finger protein 213 | 57674 | 1.14 | 0.0290004 | 0.0344 |
| 214184_at | NPFF | neuropeptide FF-amide peptide precursor | 8620 | 1.14 | 0.026507 | 0.0362 |
| 219583_s_at | SPATA7 | spermatogenesis associated 7 | 55812 | 1.14 | 0.0292139 | 0.0363 |
| 1569538_at | | | | 1.14 | 0.0339471 | 0.0394 |
| 242612_at | | | | 1.14 | 0.0298247 | 0.0398 |
| 222159_at | | | | 1.14 | 0.0296741 | 0.0403 |
| 205069_s_at | ARHGAP26 | Rho GTPase activating protein 26 | 23092 | 1.14 | 0.0313131 | 0.0425 |
| 213462_at | NPAS2 | neuronal PAS domain protein 2 | 4862 | 1.14 | 0.033917 | 0.045 |
| 228965_s_at | PANK2 | pantothenate kinase 2 | 80025 | 1.14 | 0.0411066 | 0.0456 |
| 235520_at | ZNF280C | zinc finger protein 280C | 55609 | 1.14 | 0.0380848 | 0.0482 |
| 227557_at | SCARF2 | scavenger receptor class F, member 2 | 91179 | 1.13 | 0.0011202 | 5.00E-04 |
| 243843_at | N4BP2L1 | NEDD4 binding protein 2-like 1 | 90634 | 1.13 | 0.002115 | 0.0016 |
| 235530_at | | | | 1.13 | 0.0039659 | 0.0027 |
| 232651_at | LOC1002878 | uncharacterized LOC100287813 | 100287813 | 1.13 | 0.0035448 | 0.0037 |
| 208068_x_at | | | | 1.13 | 0.0081475 | 0.0076 |
| 205234_at | SLC16A4 | solute carrier family 16, member 4 (monocarboxylic acid transporter 5) | 9122 | 1.13 | 0.0097733 | 0.011 |
| 217427_s_at | HIRA | HIR histone cell cycle regulation defective homolog A (S. cerevisiae) | 7290 | 1.13 | 0.011634 | 0.0122 |
| 240297_at | | | | 1.13 | 0.0120286 | 0.0138 |
| 226893_at | ABL2 | v-abl Abelson murine leukemia viral oncogene homolog 2 | 27 | 1.13 | 0.0140752 | 0.0141 |
| 228815_s_at | REPIN1 | replication initiator 1 | 29803 | 1.13 | 0.0120603 | 0.0143 |
| 204827_s_at | CCNF | cyclin F | 899 | 1.13 | 0.0148464 | 0.0161 |
| 210653_s_at | BCKDHB | branched chain keto acid dehydrogenase E1, beta polypeptide | 594 | 1.13 | 0.015016 | 0.0198 |
| 236063_at | | | | 1.13 | 0.0176328 | 0.0225 |
| 1555488_at | | | | 1.13 | 0.0199495 | 0.0226 |
| 223747_x_at | WWOX | WW domain containing oxidoreductase | 51741 | 1.13 | 0.0187558 | 0.0228 |
| 1554052_at | CNOT1 | CCR4-NOT transcription complex, subunit 1 | 23019 | 1.13 | 0.019167 | 0.0247 |
| 243573_at | | | | 1.13 | 0.022223 | 0.0248 |
| 243134_at | | | | 1.13 | 0.0225009 | 0.0251 |
| 212008_at | UBXN4 | UBX domain protein 4 | 23190 | 1.13 | 0.024269 | 0.0273 |
| 201749_at | ECE1 | endothelin converting enzyme 1 | 1889 | 1.13 | 0.0218649 | 0.0274 |
| 217879_at | CDC27 | cell division cycle 27 | 996 | 1.13 | 0.0234899 | 0.0279 |
| 208164_s_at | IL9R | interleukin 9 receptor | 3581 | 1.13 | 0.0230865 | 0.028 |
| 232365_at | SIAH1 | siah E3 ubiquitin protein ligase 1 | 6477 | 1.13 | 0.0252796 | 0.0308 |
| 214885_at | KAT8 | K(lysine) acetyltransferase 8 | 84148 | 1.13 | 0.0266319 | 0.0324 |
| 227429_at | EFCAB4A | EF-hand calcium binding domain 4A | 283229 | 1.13 | 0.030073 | 0.0379 |
| 230736_at | PTCHD3P1 | patched domain containing 3 pseudogene 1 | 387647 | 1.13 | 0.0369251 | 0.042 |
| 223165_s_at | IP6K2 | inositol hexakisphosphate kinase 2 | 51447 | 1.13 | 0.0352794 | 0.0427 |
| 216813_at | | | | 1.13 | 0.0306863 | 0.0436 |
| 228461_at | SH3RF3 | SH3 domain containing ring finger 3 | 344558 | 1.13 | 0.0353473 | 0.0446 |
| 219657_s_at | KLF3 | Kruppel-like factor 3 (basic) | 51274 | 1.13 | 0.0414605 | 0.0486 |
| 234126_at | | | | 1.13 | 0.0004811 | < 1e-07 |
| 1561437_at | LOC728012 | uncharacterized LOC728012 | 728012 | 1.12 | 0.002165 | 4.00E-04 |
| 211109_at | JAK3 | Janus kinase 3 | 3718 | 1.12 | 0.0022037 | 0.0016 |
| 241360_at | CCDC15 | coiled-coil domain containing 15 | 80071 | 1.12 | 0.0044756 | 0.002 |
| 205303_at | KCNJ8 | potassium inwardly-rectifying channel, subfamily J, member 8 | 3764 | 1.12 | 0.0037461 | 0.0026 |
| 203592_s_at | FSTL3 | follistatin-like 3 (secreted glycoprotein) | 10272 | 1.12 | 0.0038067 | 0.0026 |
| 59644_at | BMP2K | BMP2 inducible kinase | 55589 | 1.12 | 0.0049808 | 0.003 |
| 227238_at | MUC15 | mucin 15, cell surface associated | 143662 | 1.12 | 0.0076249 | 0.0061 |
| 222137_at | CC2D1A | coiled-coil and C2 domain containing 1A | 54862 | 1.12 | 0.0074949 | 0.0062 |
| 1557128_at | FAM111B | family with sequence similarity 111, member B | 374393 | 1.12 | 0.0117705 | 0.0094 |
| 206905_s_at | MATN1 | matrilin 1, cartilage matrix protein | 4146 | 1.12 | 0.0083362 | 0.01 |
| 243392_at | USP49 | ubiquitin specific peptidase 49 | 25862 | 1.12 | 0.0119704 | 0.01 |
| 234174_at | | | | 1.12 | 0.0114238 | 0.0116 |
| 1561488_at | | | | 1.12 | 0.0129833 | 0.012 |
| 212478_at | RMND5A | required for meiotic nuclear division 5 homolog A (S. cerevisiae) | 64795 | 1.12 | 0.0107863 | 0.0122 |
| 244640_at | ZNF850 | zinc finger protein 850 | 342892 | 1.12 | 0.0148598 | 0.0127 |
| 1561014_at | | | | 1.12 | 0.0158368 | 0.0148 |
| 1570592_a_at | | | | 1.12 | 0.0139517 | 0.016 |
| 225790_at | MSRB3 | methionine sulfoxide reductase B3 | 253827 | 1.12 | 0.0199401 | 0.0181 |
| 214969_at | MAP3K9 | mitogen-activated protein kinase kinase kinase 9 | 4293 | 1.12 | 0.0165277 | 0.0182 |
| 214513_s_at | CREB1 | cAMP responsive element binding protein 1 | 1385 | 1.12 | 0.0165704 | 0.0199 |

| ProbeSet | Symbol | Name | EntrezID | Fold Change | Parametric p-value | Permutation p-value |
|---|---|---|---|---|---|---|
| 228487_s_at | RREB1 | ras responsive element binding protein 1 | 6239 | 1.12 | 0.0227143 | 0.0199 |
| 222778_s_at | WHSC1 | Wolf-Hirschhorn syndrome candidate 1 | 7468 | 1.12 | 0.0205687 | 0.02 |
| 215811_at | | | | 1.12 | 0.0192224 | 0.0215 |
| 228836_at | SLC25A35 | solute carrier family 25, member 35 | 399512 | 1.12 | 0.0207014 | 0.0217 |
| 210232_at | CDC42 | cell division cycle 42 | 998 | 1.12 | 0.0197677 | 0.0227 |
| 235255_at | ATP6V0A2 | ATPase, H+ transporting, lysosomal V0 subunit a2 | 23545 | 1.12 | 0.0198259 | 0.024 |
| 212479_s_at | RMND5A | required for meiotic nuclear division 5 homolog A (S. cerevisiae) | 64795 | 1.12 | 0.0260456 | 0.0279 |
| 204811_s_at | CACNA2D2 | calcium channel, voltage-dependent, alpha 2/delta subunit 2 | 9254 | 1.12 | 0.0249846 | 0.0289 |
| 1559062_at | | | | 1.12 | 0.0270372 | 0.029 |
| 219410_at | TMEM45A | transmembrane protein 45A | 55076 | 1.12 | 0.0270552 | 0.0299 |
| 1559563_at | | | | 1.12 | 0.0267183 | 0.0308 |
| 223733_s_at | PPP4R1L | protein phosphatase 4, regulatory subunit 1-like | 55370 | 1.12 | 0.0256768 | 0.0312 |
| 238477_at | KIF1C | kinesin family member 1C | 10749 | 1.12 | 0.0221688 | 0.0313 |
| 219376_at | | | | 1.12 | 0.0270058 | 0.0325 |
| 207068_at | ZFP37 | ZFP37 zinc finger protein | 7539 | 1.12 | 0.0296924 | 0.0327 |
| 225567_at | LOC1005055 | uncharacterized LOC100505573 | 100505573 | 1.12 | 0.0300916 | 0.0339 |
| 236773_at | | | | 1.12 | 0.0280695 | 0.0352 |
| 219980_at | C4orf29 | chromosome 4 open reading frame 29 | 80167 | 1.12 | 0.0283469 | 0.0358 |
| 236223_s_at | RIT1 | Ras-like without CAAX 1 | 6016 | 1.12 | 0.0297708 | 0.0358 |
| 1552651_a_at | | | | 1.12 | 0.0304943 | 0.0365 |
| 242651_at | | | | 1.12 | 0.0341725 | 0.0387 |
| 1560754_at | CMTM7 | CKLF-like MARVEL transmembrane domain containing 7 | 112616 | 1.12 | 0.0318244 | 0.0392 |
| 241405_at | TOB1-AS1 | TOB1 antisense RNA 1 | 400604 | 1.12 | 0.0353723 | 0.0393 |
| 1552639_at | KLHDC7B | kelch domain containing 7B | 113730 | 1.12 | 0.0381659 | 0.0398 |
| 244461_at | SPECC1 | sperm antigen with calponin homology and coiled-coil domains 1 | 92521 | 1.12 | 0.0360243 | 0.0406 |
| 212271_at | MAPK1 | mitogen-activated protein kinase 1 | 5594 | 1.12 | 0.0350192 | 0.0411 |
| 222887_s_at | TMEM127 | transmembrane protein 127 | 55654 | 1.12 | 0.0352418 | 0.0436 |
| 1559434_at | | | | 1.12 | 0.0365384 | 0.0445 |
| 225468_at | PATL1 | protein associated with topoisomerase II homolog 1 (yeast) | 219988 | 1.12 | 0.0391558 | 0.0454 |
| 238515_at | NUDT16 | nudix (nucleoside diphosphate linked moiety X)-type motif 16 | 131870 | 1.12 | 0.0378202 | 0.0462 |
| 205420_at | PEX7 | peroxisomal biogenesis factor 7 | 5191 | 1.12 | 0.0372356 | 0.0466 |
| 230848_s_at | MGA | MGA, MAX dimerization protein | 23269 | 1.12 | 0.0365124 | 0.0477 |
| 210630_s_at | RAD52 | RAD52 homolog (S. cerevisiae) | 5893 | 1.12 | 0.0396878 | 0.0497 |
| 206089_at | NELL1 | NEL-like 1 (chicken) | 4745 | 1.12 | 0.039801 | 0.0497 |
| 207445_s_at | CCR9 | chemokine (C-C motif) receptor 9 | 10803 | 1.12 | 0.0414307 | 0.0498 |
| 213349_at | TMCC1 | transmembrane and coiled-coil domain family 1 | 23023 | 1.12 | 0.0463399 | 0.0498 |
| 1563596_at | | | | 1.11 | 0.0034966 | 0.0011 |
| 229557_at | MEG3 | maternally expressed 3 (non-protein coding) | 55384 | 1.11 | 0.0042471 | 0.0021 |
| 226739_at | RNF169 | ring finger protein 169 | 254225 | 1.11 | 0.0055932 | 0.0028 |
| 229068_at | CCT5 | chaperonin containing TCP1, subunit 5 (epsilon) | 22948 | 1.11 | 0.0059214 | 0.0035 |
| 208648_at | VCP | valosin containing protein | 7415 | 1.11 | 0.007469 | 0.0048 |
| 227775_at | CELF6 | CUGBP, Elav-like family member 6 | 60677 | 1.11 | 0.0078424 | 0.006 |
| 243383_at | | | | 1.11 | 0.0128655 | 0.0064 |
| 229729_at | TMEM8B | transmembrane protein 8B | 51754 | 1.11 | 0.0103901 | 0.0067 |
| 214842_s_at | | | | 1.11 | 0.0099811 | 0.0084 |
| 239913_at | SLC10A4 | solute carrier family 10 (sodium/bile acid cotransporter family), member 4 | 201780 | 1.11 | 0.0113103 | 0.0084 |
| 216719_s_at | RAB11FIP4 | RAB11 family interacting protein 4 (class II) | 84440 | 1.11 | 0.0118239 | 0.0084 |
| 1570251_at | HECTD1 | HECT domain containing E3 ubiquitin protein ligase 1 | 25831 | 1.11 | 0.0104499 | 0.0085 |
| 1553879_a_a | GOT1L1 | glutamic-oxaloacetic transaminase 1-like 1 | 137362 | 1.11 | 0.0119364 | 0.009 |
| 1555279_at | ARMC8 | armadillo repeat containing 8 | 25852 | 1.11 | 0.0130392 | 0.0119 |
| 241201_at | UVSSA | UV-stimulated scaffold protein A | 57654 | 1.11 | 0.015083 | 0.0124 |
| 225192_at | CACUL1 | CDK2-associated, cullin domain 1 | 143384 | 1.11 | 0.0158882 | 0.0126 |
| 216896_at | COL4A3 | collagen, type IV, alpha 3 (Goodpasture antigen) | 1285 | 1.11 | 0.0153445 | 0.013 |
| 1569450_at | CAPZA2 | capping protein (actin filament) muscle Z-line, alpha 2 | 830 | 1.11 | 0.017209 | 0.0131 |
| 229627_at | | | | 1.11 | 0.0146477 | 0.0139 |
| 221963_x_at | ZNF587B | zinc finger protein 587B | 100293516 | 1.11 | 0.0144289 | 0.0145 |
| 221954_at | C20orf111 | chromosome 20 open reading frame 111 | 51526 | 1.11 | 0.0175056 | 0.0148 |
| 1555281_x_a | ARMC8 | armadillo repeat containing 8 | 25852 | 1.11 | 0.0186155 | 0.0149 |
| 1557126_a_a | PLD1 | phospholipase D1, phosphatidylcholine-specific | 5337 | 1.11 | 0.0150984 | 0.0158 |
| 202565_s_at | SVIL | supervillin | 6840 | 1.11 | 0.018311 | 0.0176 |
| 233174_at | LOC1002870 | uncharacterized LOC100287015 | 100287015 | 1.11 | 0.0182978 | 0.0181 |
| 238444_at | ZNF618 | zinc finger protein 618 | 114991 | 1.11 | 0.0193483 | 0.0185 |
| 242295_at | FLJ32955 | uncharacterized protein FLJ32955 | 150596 | 1.11 | 0.0187205 | 0.0186 |
| 1558693_s_a | C1orf85 | chromosome 1 open reading frame 85 | 112770 | 1.11 | 0.0219324 | 0.0186 |
| 244250_at | ANXA6 | annexin A6 | 309 | 1.11 | 0.0209984 | 0.0192 |
| 210790_s_at | SAR1A | SAR1 homolog A (S. cerevisiae) | 56681 | 1.11 | 0.0227337 | 0.0201 |
| 239803_at | | | | 1.11 | 0.0216138 | 0.021 |
| 204039_at | CEBPA | CCAAT/enhancer binding protein (C/EBP), alpha | 1050 | 1.11 | 0.0228912 | 0.021 |
| 1555752_at | STH | saitohin | 246744 | 1.11 | 0.0221365 | 0.0213 |
| 206315_at | CRLF1 | cytokine receptor-like factor 1 | 9244 | 1.11 | 0.0218972 | 0.0236 |
| 1563863_x_a | TCEANC | transcription elongation factor A (SII) N-terminal and central domain containing | 170082 | 1.11 | 0.0239142 | 0.024 |
| 1557811_a_at | | | | 1.11 | 0.0242416 | 0.0244 |
| 1560848_at | | | | 1.11 | 0.0259105 | 0.0245 |
| 206007_at | PRG4 | proteoglycan 4 | 10216 | 1.11 | 0.0271131 | 0.0246 |
| 234031_at | C20orf112 | chromosome 20 open reading frame 112 | 140688 | 1.11 | 0.0269008 | 0.0254 |
| 226931_at | TMTC1 | transmembrane and tetratricopeptide repeat containing 1 | 83857 | 1.11 | 0.0308659 | 0.0266 |
| 1562745_at | | | | 1.11 | 0.0239195 | 0.0268 |
| 230107_at | | | | 1.11 | 0.0267349 | 0.0268 |

| ProbeSet | Symbol | Name | EntrezID | Fold Change | Parametric p-value | Permutation p-value |
|---|---|---|---|---|---|---|
| 203074_at | | | | 1.11 | 0.0258227 | 0.028 |
| 228069_at | MTFR2 | mitochondrial fission regulator 2 | 113115 | 1.11 | 0.0262315 | 0.0288 |
| 1561511_at | | | | 1.11 | 0.0279994 | 0.0299 |
| 232593_at | NEURL3 | neuralized homolog 3 (Drosophila) pseudogene | 93082 | 1.11 | 0.0298496 | 0.0308 |
| 221210_s_at | NPL | N-acetylneuraminate pyruvate lyase (dihydrodipicolinate synthase) | 80896 | 1.11 | 0.0300599 | 0.0312 |
| 1552455_at | PRUNE2 | prune homolog 2 (Drosophila) | 158471 | 1.11 | 0.0287425 | 0.0316 |
| 213609_s_at | SEZ6L | seizure related 6 homolog (mouse)-like | 23544 | 1.11 | 0.0301122 | 0.0317 |
| 215622_x_at | PHF7 | PHD finger protein 7 | 51533 | 1.11 | 0.0309927 | 0.0333 |
| 212211_at | ANKRD17 | ankyrin repeat domain 17 | 26057 | 1.11 | 0.0325272 | 0.0337 |
| 1556945_a_at | | | | 1.11 | 0.0325571 | 0.0347 |
| 230132_at | LOC100505495 | uncharacterized LOC100505495 | 100505495 | 1.11 | 0.0347678 | 0.039 |
| 205060_at | PARG | poly (ADP-ribose) glycohydrolase | 8505 | 1.11 | 0.0363072 | 0.0394 |
| 235089_at | FBXL20 | F-box and leucine-rich repeat protein 20 | 84961 | 1.11 | 0.0363586 | 0.0404 |
| 221550_at | COX15 | cytochrome c oxidase assembly homolog 15 (yeast) | 1355 | 1.11 | 0.0382391 | 0.0404 |
| 215484_at | | | | 1.11 | 0.0356727 | 0.0413 |
| 205810_s_at | WASL | Wiskott-Aldrich syndrome-like | 8976 | 1.11 | 0.0403415 | 0.0418 |
| 206588_at | DAZL | deleted in azoospermia-like | 1618 | 1.11 | 0.0357758 | 0.0419 |
| 1555853_at | LOC100507463 | uncharacterized LOC100507463 | 100507463 | 1.11 | 0.0388597 | 0.0419 |
| 219297_at | WDR44 | WD repeat domain 44 | 54521 | 1.11 | 0.0373969 | 0.0433 |
| 237061_at | ZNF347 | zinc finger protein 347 | 84671 | 1.11 | 0.0380932 | 0.0439 |
| 233306_at | | | | 1.11 | 0.0468753 | 0.0447 |
| 240401_at | | | | 1.11 | 0.0425986 | 0.0476 |
| 226183_at | GSK3B | glycogen synthase kinase 3 beta | 2932 | 1.11 | 0.0402697 | 0.0478 |
| 243316_x_at | VPS26A | vacuolar protein sorting 26 homolog A (S. pombe) | 9559 | 1.11 | 0.0402853 | 0.048 |
| 1566951_at | | | | 1.11 | 0.043161 | 0.0481 |
| 1554334_a_at | DNAJA4 | DnaJ (Hsp40) homolog, subfamily A, member 4 | 55466 | 1.11 | 0.0416131 | 0.0488 |
| 1559144_x_at | LOC100130581 | uncharacterized LOC100130581 | 100130581 | 1.11 | 0.0414085 | 0.0495 |
| 222335_at | | | | 1.11 | 0.0455861 | 0.0499 |
| 241022_at | | | | 1.1 | 0.0022208 | 1.00E-04 |
| 217896_s_at | FAM192A | family with sequence similarity 192, member A | 80011 | 1.1 | 0.0035956 | 6.00E-04 |
| 228739_at | CYS1 | cystin 1 | 192668 | 1.1 | 0.0027642 | 0.0012 |
| 244036_at | | | | 1.1 | 0.0042356 | 0.0018 |
| 209834_at | CHST3 | carbohydrate (chondroitin 6) sulfotransferase 3 | 9469 | 1.1 | 0.0083957 | 0.0039 |
| 239220_at | | | | 1.1 | 0.009945 | 0.0061 |
| 244629_s_at | PDPK1 | 3-phosphoinositide dependent protein kinase-1 | 5170 | 1.1 | 0.0119897 | 0.0087 |
| 218455_at | NFS1 | NFS1 nitrogen fixation 1 homolog (S. cerevisiae) | 9054 | 1.1 | 0.0131774 | 0.0089 |
| 212275_s_at | SRCAP | Snf2-related CREBBP activator protein | 10847 | 1.1 | 0.0133286 | 0.0089 |
| 203564_at | FANCG | Fanconi anemia, complementation group G | 2189 | 1.1 | 0.0187825 | 0.01 |
| 243074_at | | | | 1.1 | 0.0146843 | 0.0103 |
| 1566637_at | | | | 1.1 | 0.0155933 | 0.0103 |
| 235263_at | | | | 1.1 | 0.0183212 | 0.0112 |
| 47773_at | FBXO42 | F-box protein 42 | 54455 | 1.1 | 0.014233 | 0.0115 |
| 1562426_a_at | FARP1 | FERM, RhoGEF (ARHGEF) and pleckstrin domain protein 1 (chondrocyte-derived) | 10160 | 1.1 | 0.0147542 | 0.0127 |
| 1555151_s_at | TDH | L-threonine dehydrogenase | 157739 | 1.1 | 0.0182503 | 0.0133 |
| 231915_at | ZSWIM4 | zinc finger, SWIM-type containing 4 | 65249 | 1.1 | 0.0174984 | 0.0141 |
| 228783_at | BVES | blood vessel epicardial substance | 11149 | 1.1 | 0.0189155 | 0.0144 |
| 53912_at | SNX11 | sorting nexin 11 | 29916 | 1.1 | 0.0189213 | 0.0169 |
| 236083_at | BCL2L15 | BCL2-like 15 | 440603 | 1.1 | 0.0197162 | 0.0169 |
| 1566265_at | | | | 1.1 | 0.022004 | 0.0174 |
| 1552836_at | ZNF619 | zinc finger protein 619 | 285267 | 1.1 | 0.0179272 | 0.0175 |
| 1556620_at | | | | 1.1 | 0.0213369 | 0.0178 |
| 231182_at | WIPF1 | WAS/WASL interacting protein family, member 1 | 7456 | 1.1 | 0.0220059 | 0.018 |
| 240620_at | | | | 1.1 | 0.0205599 | 0.0182 |
| 216119_s_at | SPEF1 | sperm flagellar 1 | 25876 | 1.1 | 0.0180741 | 0.0187 |
| 215185_at | | | | 1.1 | 0.0233088 | 0.0189 |
| 206620_at | GRAP | GRB2-related adaptor protein | 10750 | 1.1 | 0.0197486 | 0.0192 |
| 236121_at | OR51E2 | olfactory receptor, family 51, subfamily E, member 2 | 81285 | 1.1 | 0.0233978 | 0.0196 |
| 205987_at | CD1C | CD1c molecule | 911 | 1.1 | 0.0334789 | 0.02 |
| 239752_at | | | | 1.1 | 0.0216746 | 0.0208 |
| 237049_at | | | | 1.1 | 0.0229844 | 0.0221 |
| 205633_s_at | ALAS1 | aminolevulinate, delta-, synthase 1 | 211 | 1.1 | 0.023984 | 0.0226 |
| 37986_at | EPOR | erythropoietin receptor | 2057 | 1.1 | 0.0270128 | 0.0228 |
| 205704_s_at | ATP6V0A2 | ATPase, H+ transporting, lysosomal V0 subunit a2 | 23545 | 1.1 | 0.0266569 | 0.0234 |
| 1557796_at | FAR2 | fatty acyl CoA reductase 2 | 55711 | 1.1 | 0.0251156 | 0.0237 |
| 230427_s_at | BAG5 | BCL2-associated athanogene 5 | 9529 | 1.1 | 0.0246715 | 0.0238 |
| 236977_at | LOC646588 | uncharacterized LOC646588 | 646588 | 1.1 | 0.0278926 | 0.0241 |
| 209778_at | TRIP11 | thyroid hormone receptor interactor 11 | 9321 | 1.1 | 0.0274963 | 0.0248 |
| 233954_at | HIATL1 | hippocampus abundant transcript-like 1 | 84641 | 1.1 | 0.0286667 | 0.0256 |
| 243504_at | | | | 1.1 | 0.0240898 | 0.0263 |
| 212174_at | AK2 | adenylate kinase 2 | 204 | 1.1 | 0.0276843 | 0.0263 |
| 1561738_at | | | | 1.1 | 0.0308351 | 0.0291 |
| 238772_at | ZNF207 | zinc finger protein 207 | 7756 | 1.1 | 0.0302767 | 0.0296 |
| 210257_x_at | CUL4B | cullin 4B | 8450 | 1.1 | 0.0314995 | 0.0302 |
| 227476_at | LPGAT1 | lysophosphatidylglycerol acyltransferase 1 | 9926 | 1.1 | 0.0328584 | 0.0303 |
| 214520_at | FOXC2 | forkhead box C2 (MFH-1, mesenchyme forkhead 1) | 2303 | 1.1 | 0.0333626 | 0.0303 |
| 201048_x_at | RAB6A | RAB6A, member RAS oncogene family | 5870 | 1.1 | 0.0325999 | 0.0309 |
| 225138_at | ZRANB1 | zinc finger, RAN-binding domain containing 1 | 54764 | 1.1 | 0.0335994 | 0.0319 |
| 1565662_at | MUC6 | mucin 6, oligomeric mucus/gel-forming | 4588 | 1.1 | 0.0317826 | 0.0325 |

| ProbeSet | Symbol | Name | EntrezID | Fold Change | Parametric p-value | Permutation p-value |
|---|---|---|---|---|---|---|
| 220250_at | ZNF286A | zinc finger protein 286A | 57335 | 1.1 | 0.0358779 | 0.0329 |
| 203704_s_at | RREB1 | ras responsive element binding protein 1 | 6239 | 1.1 | 0.0378853 | 0.033 |
| 1562790_at | NCALD | neurocalcin delta | 83988 | 1.1 | 0.0323466 | 0.0344 |
| 223592_s_at | RNF135 | ring finger protein 135 | 84282 | 1.1 | 0.0341654 | 0.0344 |
| 231133_at | CCDC164 | coiled-coil domain containing 164 | 92749 | 1.1 | 0.0349115 | 0.0351 |
| 1556059_s_a | SPEN | spen homolog, transcriptional regulator (Drosophila) | 23013 | 1.1 | 0.0403555 | 0.0352 |
| 1554554_at | CCDC57 | coiled-coil domain containing 57 | 284001 | 1.1 | 0.0392383 | 0.0353 |
| 228849_at | NTRK3 | neurotrophic tyrosine kinase, receptor, type 3 | 4916 | 1.1 | 0.0361695 | 0.0377 |
| 243570_at | SPCS2 | signal peptidase complex subunit 2 homolog (S. cerevisiae) | 9789 | 1.1 | 0.0380444 | 0.0377 |
| 236605_at | EIF3K | eukaryotic translation initiation factor 3, subunit K | 27335 | 1.1 | 0.0431954 | 0.04 |
| 243477_at | ATG10 | autophagy related 10 | 83734 | 1.1 | 0.0398358 | 0.0403 |
| 218942_at | PIP4K2C | phosphatidylinositol-5-phosphate 4-kinase, type II, gamma | 79837 | 1.1 | 0.0403839 | 0.0404 |
| 216501_at | VAC14 | Vac14 homolog (S. cerevisiae) | 55697 | 1.1 | 0.0412865 | 0.0407 |
| 240057_at | | | | 1.1 | 0.0403155 | 0.0409 |
| 235462_at | CPEB2 | cytoplasmic polyadenylation element binding protein 2 | 132864 | 1.1 | 0.0401722 | 0.0415 |
| 237661_at | LOC1005069 | uncharacterized LOC100506997 | 100506997 | 1.1 | 0.0407118 | 0.0416 |
| 215146_s_at | TTC28 | tetratricopeptide repeat domain 28 | 23331 | 1.1 | 0.0387806 | 0.0419 |
| 201799_s_at | OSBP | oxysterol binding protein | 5007 | 1.1 | 0.0447041 | 0.0419 |
| 244236_at | | | | 1.1 | 0.0413862 | 0.0434 |
| 222015_at | CSNK1E | casein kinase 1, epsilon | 1454 | 1.1 | 0.0405401 | 0.0437 |
| 236213_at | | | | 1.1 | 0.0427549 | 0.0444 |
| 242056_at | TRIM45 | tripartite motif containing 45 | 80263 | 1.1 | 0.0403957 | 0.0454 |
| 203843_at | RPS6KA3 | ribosomal protein S6 kinase, 90kDa, polypeptide 3 | 6197 | 1.1 | 0.0475385 | 0.0455 |
| 239851_at | | | | 1.1 | 0.0440708 | 0.046 |
| 240311_at | NANOS3 | nanos homolog 3 (Drosophila) | 342977 | 1.1 | 0.0474375 | 0.0469 |
| 203195_s_at | NUP98 | nucleoporin 98kDa | 4928 | 1.1 | 0.0449955 | 0.0471 |
| 215767_at | ZNF804A | zinc finger protein 804A | 91752 | 1.1 | 0.0459466 | 0.0476 |
| 235415_at | RPRD2 | regulation of nuclear pre-mRNA domain containing 2 | 23248 | 1.1 | 0.0433104 | 0.0484 |
| 232202_at | FAM83B | family with sequence similarity 83, member B | 222584 | 1.09 | 0.0080402 | 0.0017 |
| 213557_at | | | | 1.09 | 0.0064963 | 0.0023 |
| 1556084_at | HNRNPM | heterogeneous nuclear ribonucleoprotein M | 4670 | 1.09 | 0.0086017 | 0.0029 |
| 1558578_a_at | | | | 1.09 | 0.0082207 | 0.0043 |
| 238486_at | FRS2 | fibroblast growth factor receptor substrate 2 | 10818 | 1.09 | 0.011409 | 0.005 |
| 221089_at | NOX3 | NADPH oxidase 3 | 50508 | 1.09 | 0.0137804 | 0.0057 |
| 232303_at | ZNF608 | zinc finger protein 608 | 57507 | 1.09 | 0.0127305 | 0.0066 |
| 205535_s_at | PCDH7 | protocadherin 7 | 5099 | 1.09 | 0.0186741 | 0.0067 |
| 229800_at | DCLK1 | doublecortin-like kinase 1 | 9201 | 1.09 | 0.0139859 | 0.0078 |
| 232039_at | MAP10 | microtubule-associated protein 10 | 54627 | 1.09 | 0.0133477 | 0.0081 |
| 236031_x_at | FREM1 | FRAS1 related extracellular matrix 1 | 158326 | 1.09 | 0.0194067 | 0.0082 |
| 220254_at | LRP12 | low density lipoprotein receptor-related protein 12 | 29967 | 1.09 | 0.0130583 | 0.0087 |
| 242504_at | | | | 1.09 | 0.0191046 | 0.0088 |
| 221238_at | HMGN5 | high mobility group nucleosome binding domain 5 | 79366 | 1.09 | 0.0182352 | 0.0096 |
| 242496_at | ART4 | ADP-ribosyltransferase 4 (Dombrock blood group) | 420 | 1.09 | 0.0182353 | 0.0097 |
| 1557373_at | LOC339505 | uncharacterized LOC339505 | 339505 | 1.09 | 0.0185399 | 0.01 |
| 1557873_at | | | | 1.09 | 0.0188123 | 0.0103 |
| 236581_at | | | | 1.09 | 0.0190439 | 0.0107 |
| 212252_at | CAMKK2 | calcium/calmodulin-dependent protein kinase kinase 2, beta | 10645 | 1.09 | 0.0197581 | 0.0119 |
| 229861_at | RFFL | ring finger and FYVE-like domain containing E3 ubiquitin protein ligase | 117584 | 1.09 | 0.020232 | 0.0123 |
| 238450_at | PFKFB2 | 6-phosphofructo-2-kinase/fructose-2,6-biphosphatase 2 | 5208 | 1.09 | 0.0221601 | 0.0136 |
| 220781_at | 1-Dec | deleted in esophageal cancer 1 | 50514 | 1.09 | 0.0185677 | 0.0141 |
| 224409_s_at | TSSK6 | testis-specific serine kinase 6 | 83983 | 1.09 | 0.0227748 | 0.0145 |
| 229844_at | FOXP1 | forkhead box P1 | 27086 | 1.09 | 0.0239455 | 0.0149 |
| 224423_x_at | PMCHL2 | pro-melanin-concentrating hormone-like 2, pseudogene | 5370 | 1.09 | 0.028888 | 0.0169 |
| 1566471_at | LOC1009965 | all-trans-retinol 13,14-reductase-like | 100996506 | 1.09 | 0.0279286 | 0.017 |
| 1556081_at | | | | 1.09 | 0.0250695 | 0.0175 |
| 1556097_at | HOMER2 | homer homolog 2 (Drosophila) | 9455 | 1.09 | 0.0219364 | 0.0192 |
| 234591_at | | | | 1.09 | 0.0251932 | 0.0194 |
| 229650_s_at | SMIM7 | small integral membrane protein 7 | 79086 | 1.09 | 0.0287884 | 0.021 |
| 221812_at | FBXO42 | F-box protein 42 | 54455 | 1.09 | 0.0288583 | 0.0219 |
| 203205_at | KDM4A | lysine (K)-specific demethylase 4A | 9682 | 1.09 | 0.0277564 | 0.0224 |
| 236642_at | C5orf63 | chromosome 5 open reading frame 63 | 401207 | 1.09 | 0.0318793 | 0.023 |
| 214706_at | ZNF200 | zinc finger protein 200 | 7752 | 1.09 | 0.0318992 | 0.0232 |
| 242583_at | STON2 | stonin 2 | 85439 | 1.09 | 0.0292399 | 0.0237 |
| 206044_s_at | BRAF | v-raf murine sarcoma viral oncogene homolog B1 | 673 | 1.09 | 0.0306096 | 0.0239 |
| 205536_at | VAV2 | vav 2 guanine nucleotide exchange factor | 7410 | 1.09 | 0.0335681 | 0.0244 |
| 204982_at | GIT2 | G protein-coupled receptor kinase interacting ArfGAP 2 | 9815 | 1.09 | 0.0313888 | 0.025 |
| 235533_at | COX19 | cytochrome c oxidase assembly homolog 19 (S. cerevisiae) | 90639 | 1.09 | 0.0342969 | 0.027 |
| 216106_at | LOC145678 | uncharacterized LOC145678 | 145678 | 1.09 | 0.0302615 | 0.0272 |
| 1552365_at | SCIN | scinderin | 85477 | 1.09 | 0.0314819 | 0.0277 |
| 209963_s_at | EPOR | erythropoietin receptor | 2057 | 1.09 | 0.0319192 | 0.0294 |
| 220283_at | HHIPL2 | HHIP-like 2 | 79802 | 1.09 | 0.035871 | 0.031 |
| 234208_at | | | | 1.09 | 0.0383766 | 0.0314 |
| 223146_at | | | | 1.09 | 0.0376934 | 0.0316 |
| 224380_s_at | TAF7L | TAF7-like RNA polymerase II, TATA box binding protein (TBP)-associated factor, 50kDa | 54457 | 1.09 | 0.0393043 | 0.032 |
| 205675_at | MTTP | microsomal triglyceride transfer protein | 4547 | 1.09 | 0.0346767 | 0.0324 |
| 226954_at | UBE2R2 | ubiquitin-conjugating enzyme E2R 2 | 54926 | 1.09 | 0.0419365 | 0.0329 |
| 243728_at | | | | 1.09 | 0.0365316 | 0.0332 |
| 242353_at | | | | 1.09 | 0.0373511 | 0.0332 |

| ProbeSet | Symbol | Name | EntrezID | Fold Change | Parametric p-value | Permutation p-value |
|---|---|---|---|---|---|---|
| 1556398_a_at | | | | 1.09 | 0.0404789 | 0.0337 |
| 1570320_at | | | | 1.09 | 0.0412991 | 0.0342 |
| 1558601_at | LSAMP-AS3 | LSAMP antisense RNA 3 | 285194 | 1.09 | 0.036309 | 0.0347 |
| 237085_x_at | | | | 1.09 | 0.0358714 | 0.0348 |
| 210221_at | CHRNA3 | cholinergic receptor, nicotinic, alpha 3 (neuronal) | 1136 | 1.09 | 0.0393448 | 0.035 |
| 230396_at | SAMM50 | sorting and assembly machinery component 50 homolog (S. cerevisiae) | 25813 | 1.09 | 0.0405393 | 0.0353 |
| 224639_at | SPPL3 | signal peptide peptidase like 3 | 121665 | 1.09 | 0.040832 | 0.0374 |
| 227618_at | | | | 1.09 | 0.0492415 | 0.0376 |
| 215065_at | PHF8 | PHD finger protein 8 | 23133 | 1.09 | 0.0453131 | 0.0385 |
| 200776_s_at | BZW1 | basic leucine zipper and W2 domains 1 | 9689 | 1.09 | 0.0442373 | 0.04 |
| 230872_s_at | | | | 1.09 | 0.0410695 | 0.0405 |
| 1568845_at | | | | 1.09 | 0.0457401 | 0.0408 |
| 237370_at | | | | 1.09 | 0.0453142 | 0.0415 |
| 206134_at | ADAMDEC1 | ADAM-like, decysin 1 | 27299 | 1.09 | 0.0394015 | 0.0417 |
| 218137_s_at | SMAP1 | small ArfGAP 1 | 60682 | 1.09 | 0.0440218 | 0.0417 |
| 1557818_x_at | | | | 1.09 | 0.0448846 | 0.0417 |
| 214329_x_at | TNFSF10 | tumor necrosis factor (ligand) superfamily, member 10 | 8743 | 1.09 | 0.0455947 | 0.0421 |
| 1554916_a_a | JRK | jerky homolog (mouse) | 8629 | 1.09 | 0.0458065 | 0.0435 |
| 204532_x_at | | | | 1.09 | 0.0478838 | 0.0436 |
| 227554_at | MAGI2-AS3 | MAGI2 antisense RNA 3 | 100505881 | 1.09 | 0.0432376 | 0.0437 |
| 243669_s_at | PRAP1 | proline-rich acidic protein 1 | 118471 | 1.09 | 0.0441845 | 0.044 |
| 207519_at | SLC6A4 | solute carrier family 6 (neurotransmitter transporter, serotonin), member 4 | 6532 | 1.09 | 0.0468288 | 0.0442 |
| 241935_at | SHROOM1 | shroom family member 1 | 134549 | 1.09 | 0.0455799 | 0.0446 |
| 233196_at | | | | 1.09 | 0.0461294 | 0.0446 |
| 231652_at | | | | 1.09 | 0.0482398 | 0.0446 |
| 1565768_at | ZNF268 | zinc finger protein 268 | 10795 | 1.09 | 0.0481778 | 0.0455 |
| 221116_at | | | | 1.09 | 0.0473568 | 0.0463 |
| 238877_at | EYA4 | eyes absent homolog 4 (Drosophila) | 2070 | 1.09 | 0.0453967 | 0.047 |
| 212209_at | MED13L | mediator complex subunit 13-like | 23389 | 1.09 | 0.0472593 | 0.048 |
| 37028_at | PPP1R15A | protein phosphatase 1, regulatory subunit 15A | 23645 | 1.09 | 0.049866 | 0.0497 |
| 240910_at | | | | 1.08 | 0.0118106 | 0.0023 |
| 204128_s_at | RFC3 | replication factor C (activator 1) 3, 38kDa | 5983 | 1.08 | 0.0117874 | 0.0032 |
| 1569634_at | SEPSECS | Sep (O-phosphoserine) tRNA:Sec (selenocysteine) tRNA synthase | 51091 | 1.08 | 0.0134642 | 0.0032 |
| 229984_at | DTWD1 | DTW domain containing 1 | 56986 | 1.08 | 0.0120449 | 0.0035 |
| 210918_at | | | | 1.08 | 0.0167389 | 0.004 |
| 222112_at | EPS15L1 | epidermal growth factor receptor pathway substrate 15-like 1 | 58513 | 1.08 | 0.0179429 | 0.0042 |
| 217103_at | LDLR | low density lipoprotein receptor | 3949 | 1.08 | 0.0163388 | 0.005 |
| 242601_at | HEPACAM2 | HEPACAM family member 2 | 253012 | 1.08 | 0.0169274 | 0.0054 |
| 228086_at | STK33 | serine/threonine kinase 33 | 65975 | 1.08 | 0.0200143 | 0.0066 |
| 244775_at | | | | 1.08 | 0.0204532 | 0.0066 |
| 242000_at | CASD1 | CAS1 domain containing 1 | 64921 | 1.08 | 0.0204006 | 0.0086 |
| 1556216_s_at | | | | 1.08 | 0.0214695 | 0.0093 |
| 244325_at | LINC00690 | long intergenic non-protein coding RNA 690 | 100996597 | 1.08 | 0.0227818 | 0.0094 |
| 239820_at | | | | 1.08 | 0.0197079 | 0.0097 |
| 225459_at | AMOTL1 | angiomotin like 1 | 154810 | 1.08 | 0.0192028 | 0.0099 |
| 208349_at | TRPA1 | transient receptor potential cation channel, subfamily A, member 1 | 8989 | 1.08 | 0.0223547 | 0.01 |
| 205325_at | PHYHIP | phytanoyl-CoA 2-hydroxylase interacting protein | 9796 | 1.08 | 0.0262467 | 0.0105 |
| 1561316_at | GABRB3 | gamma-aminobutyric acid (GABA) A receptor, beta 3 | 2562 | 1.08 | 0.0218582 | 0.0116 |
| 1570235_at | MGC27382 | uncharacterized MGC27382 | 149047 | 1.08 | 0.0228929 | 0.0117 |
| 226690_at | ADCYAP1R1 | adenylate cyclase activating polypeptide 1 (pituitary) receptor type I | 117 | 1.08 | 0.0217982 | 0.0118 |
| 201088_at | KPNA2 | karyopherin alpha 2 (RAG cohort 1, importin alpha 1) | 3838 | 1.08 | 0.0275978 | 0.0124 |
| 219789_at | NPR3 | natriuretic peptide receptor C/guanylate cyclase C (atrionatriuretic peptide receptor C) | 4883 | 1.08 | 0.0277878 | 0.0137 |
| 220422_at | UBQLN3 | ubiquilin 3 | 50613 | 1.08 | 0.0267245 | 0.0141 |
| 233494_at | ERBB4 | v-erb-a erythroblastic leukemia viral oncogene homolog 4 (avian) | 2066 | 1.08 | 0.026562 | 0.0146 |
| 1558383_at | KPNA4 | karyopherin alpha 4 (importin alpha 3) | 3840 | 1.08 | 0.0288072 | 0.015 |
| 221395_at | TAS2R13 | taste receptor, type 2, member 13 | 50838 | 1.08 | 0.0289169 | 0.0153 |
| 227094_at | DHTKD1 | dehydrogenase E1 and transketolase domain containing 1 | 55526 | 1.08 | 0.0298812 | 0.0155 |
| 237936_at | | | | 1.08 | 0.0247774 | 0.0167 |
| 232640_at | COMMD5 | COMM domain containing 5 | 28991 | 1.08 | 0.0289034 | 0.0169 |
| 240711_at | | | | 1.08 | 0.0322887 | 0.0173 |
| 1559928_at | PAPPA | pregnancy-associated plasma protein A, pappalysin 1 | 5069 | 1.08 | 0.0314781 | 0.0176 |
| 1561135_at | | | | 1.08 | 0.0312288 | 0.0187 |
| 211457_at | GABARAPL3 | GABA(A) receptors associated protein like 3, pseudogene | 23766 | 1.08 | 0.032611 | 0.0205 |
| 207728_at | ATF7IP | activating transcription factor 7 interacting protein | 55729 | 1.08 | 0.0350428 | 0.0209 |
| 1555517_at | GABRG3 | gamma-aminobutyric acid (GABA) A receptor, gamma 3 | 2567 | 1.08 | 0.0360998 | 0.0211 |
| 1557225_at | ASPG | asparaginase homolog (S. cerevisiae) | 374569 | 1.08 | 0.0354023 | 0.0216 |
| 221729_at | COL5A2 | collagen, type V, alpha 2 | 1290 | 1.08 | 0.0371498 | 0.022 |
| 213066_at | RUSC2 | RUN and SH3 domain containing 2 | 9853 | 1.08 | 0.033351 | 0.0224 |
| 218964_at | ARID3B | AT rich interactive domain 3B (BRIGHT-like) | 10620 | 1.08 | 0.0344914 | 0.0231 |
| 224067_at | | | | 1.08 | 0.0347114 | 0.0231 |
| 239177_at | IRGQ | immunity-related GTPase family, Q | 126298 | 1.08 | 0.0327428 | 0.0234 |
| 235842_at | | | | 1.08 | 0.0375005 | 0.0237 |
| 218443_s_at | DAZAP1 | DAZ associated protein 1 | 26528 | 1.08 | 0.0386202 | 0.0243 |
| 203600_s_at | FAM193A | family with sequence similarity 193, member A | 8603 | 1.08 | 0.0410877 | 0.0245 |
| 1552502_s_a | RHBDL2 | rhomboid, veinlet-like 2 (Drosophila) | 54933 | 1.08 | 0.033924 | 0.0271 |
| 201997_s_at | SPEN | spen homolog, transcriptional regulator (Drosophila) | 23013 | 1.08 | 0.0390336 | 0.0276 |
| 215515_at | | | | 1.08 | 0.0404328 | 0.0285 |
| 220513_at | | | | 1.08 | 0.0409233 | 0.0288 |

| ProbeSet | Symbol | Name | EntrezID | Fold Change | Parametric p-value | Permutation p-value |
|---|---|---|---|---|---|---|
| 207981_s_at | ESRRG | estrogen-related receptor gamma | 2104 | 1.08 | 0.0447707 | 0.029 |
| 239470_at | C15orf56 | chromosome 15 open reading frame 56 | 644809 | 1.08 | 0.0421735 | 0.0293 |
| 211843_x_at | CYP3A7-CYP3 | CYP3A7-CYP3AP1 readthrough | 100861540 | 1.08 | 0.045853 | 0.0314 |
| 231815_at | PHF12 | PHD finger protein 12 | 57649 | 1.08 | 0.0383843 | 0.0322 |
| 211870_s_at | PCDHA3 | protocadherin alpha 3 | 56145 | 1.08 | 0.0417556 | 0.0326 |
| 1565329_at | POLE4 | polymerase (DNA-directed), epsilon 4, accessory subunit | 56655 | 1.08 | 0.0448716 | 0.0331 |
| 210198_s_at | PLP1 | proteolipid protein 1 | 5354 | 1.08 | 0.0429951 | 0.0332 |
| 221498_at | SNX27 | sorting nexin family member 27 | 81609 | 1.08 | 0.0479042 | 0.0332 |
| 234642_at | | | | 1.08 | 0.0444672 | 0.0336 |
| 216800_at | | | | 1.08 | 0.0408427 | 0.0337 |
| 1555216_a_at | | | | 1.08 | 0.0393977 | 0.0339 |
| 237721_s_at | ASB4 | ankyrin repeat and SOCS box containing 4 | 51666 | 1.08 | 0.0432553 | 0.0345 |
| 203837_at | MAP3K5 | mitogen-activated protein kinase kinase kinase 5 | 4217 | 1.08 | 0.0438992 | 0.0367 |
| 1563519_at | | | | 1.08 | 0.0494525 | 0.0387 |
| 208214_at | ADRB1 | adrenoceptor beta 1 | 153 | 1.08 | 0.0486657 | 0.0388 |
| 210171_s_at | CREM | cAMP responsive element modulator | 1390 | 1.08 | 0.0484076 | 0.0407 |
| 230593_at | GRIK3 | glutamate receptor, ionotropic, kainate 3 | 2899 | 1.07 | 0.0168225 | 0.0017 |
| 1554296_at | CYP19A1 | cytochrome P450, family 19, subfamily A, polypeptide 1 | 1588 | 1.07 | 0.0191453 | 0.002 |
| 231791_at | ASAH2B | N-acylsphingosine amidohydrolase (non-lysosomal ceramidase) 2B | 653308 | 1.07 | 0.0146373 | 0.0024 |
| 231614_at | NOVA1 | neuro-oncological ventral antigen 1 | 4857 | 1.07 | 0.0220758 | 0.0034 |
| 1561478_at | LINC00560 | long intergenic non-protein coding RNA 560 | 100861553 | 1.07 | 0.0250053 | 0.0034 |
| 242137_at | RBMS3 | RNA binding motif, single stranded interacting protein 3 | 27303 | 1.07 | 0.0177498 | 0.0041 |
| 244251_at | LCP2 | lymphocyte cytosolic protein 2 (SH2 domain containing leukocyte protein of 76kDa) | 3937 | 1.07 | 0.0217154 | 0.0055 |
| 217392_at | | | | 1.07 | 0.0206172 | 0.0062 |
| 1556877_at | | | | 1.07 | 0.0234444 | 0.0081 |
| 225118_at | SETD8 | SET domain containing (lysine methyltransferase) 8 | 387893 | 1.07 | 0.0232884 | 0.0085 |
| 1562766_at | | | | 1.07 | 0.0364759 | 0.0118 |
| 1560980_a_at | | | | 1.07 | 0.0321555 | 0.0119 |
| 243903_at | | | | 1.07 | 0.0263039 | 0.0121 |
| 1560491_at | | | | 1.07 | 0.0327252 | 0.0121 |
| 1561612_at | | | | 1.07 | 0.0299669 | 0.0131 |
| 223872_at | | | | 1.07 | 0.0303548 | 0.0132 |
| 233603_at | | | | 1.07 | 0.0308419 | 0.0133 |
| 213100_at | UNC5B | unc-5 homolog B (C. elegans) | 219699 | 1.07 | 0.0287005 | 0.0135 |
| 213656_s_at | KLC1 | kinesin light chain 1 | 3831 | 1.07 | 0.0424229 | 0.0136 |
| 228640_at | PCDH7 | protocadherin 7 | 5099 | 1.07 | 0.0375087 | 0.0167 |
| 213125_at | OLFML2B | olfactomedin-like 2B | 25903 | 1.07 | 0.0325502 | 0.0182 |
| 211976_at | | | | 1.07 | 0.0425967 | 0.0186 |
| 231803_at | FGF11 | fibroblast growth factor 11 | 2256 | 1.07 | 0.0440511 | 0.0191 |
| 201057_s_at | GOLGB1 | golgin B1 | 2804 | 1.07 | 0.0372778 | 0.0192 |
| 221382_at | | | | 1.07 | 0.0377894 | 0.0197 |
| 241684_at | | | | 1.07 | 0.0438896 | 0.0203 |
| 222063_s_at | CDS1 | CDP-diacylglycerol synthase (phosphatidate cytidylyltransferase) 1 | 1040 | 1.07 | 0.0460429 | 0.0203 |
| 217335_at | FLJ11292 | uncharacterized protein FLJ11292 | 55338 | 1.07 | 0.0482007 | 0.0204 |
| 235016_at | REEP3 | receptor accessory protein 3 | 221035 | 1.07 | 0.0409657 | 0.0205 |
| 221697_at | MAP1LC3C | microtubule-associated protein 1 light chain 3 gamma | 440738 | 1.07 | 0.037481 | 0.0209 |
| 235570_at | RBMS3 | RNA binding motif, single stranded interacting protein 3 | 27303 | 1.07 | 0.0407238 | 0.0218 |
| 228443_s_at | SETD8 | SET domain containing (lysine methyltransferase) 8 | 387893 | 1.07 | 0.0378841 | 0.0231 |
| 237889_s_at | LOC1004227 | uncharacterized LOC100422737 | 100422737 | 1.07 | 0.0409469 | 0.0236 |
| 205765_at | CYP3A5 | cytochrome P450, family 3, subfamily A, polypeptide 5 | 1577 | 1.07 | 0.0391898 | 0.0242 |
| 1552506_at | CRB2 | crumbs homolog 2 (Drosophila) | 286204 | 1.07 | 0.045489 | 0.0246 |
| 1569417_at | | | | 1.07 | 0.0452213 | 0.0248 |
| 1561076_at | | | | 1.07 | 0.0423642 | 0.0263 |
| 215446_s_at | LOX | lysyl oxidase | 4015 | 1.07 | 0.0465266 | 0.0265 |
| 1562094_at | | | | 1.07 | 0.0489056 | 0.0286 |
| 243184_at | | | | 1.07 | 0.0493433 | 0.029 |
| 201315_x_at | IFITM2 | interferon induced transmembrane protein 2 | 10581 | 1.07 | 0.0458953 | 0.0296 |
| 205185_at | SPINK5 | serine peptidase inhibitor, Kazal type 5 | 11005 | 1.07 | 0.0494537 | 0.0306 |
| 241075_at | RIMKLA | ribosomal modification protein rimK-like family member A | 284716 | 1.07 | 0.0472932 | 0.0319 |
| 1552791_a_a | TRDN | triadin | 10345 | 1.07 | 0.0457714 | 0.0325 |
| 222271_at | | | | 1.07 | 0.0491826 | 0.033 |
| 220200_s_at | SETD8 | SET domain containing (lysine methyltransferase) 8 | 387893 | 1.07 | 0.0495137 | 0.0352 |
| 1567288_at | OR5K1 | olfactory receptor, family 5, subfamily K, member 1 | 26339 | 1.06 | 0.0398428 | 0.0022 |
| 1561030_at | TMC7 | transmembrane channel-like 7 | 79905 | 1.06 | 0.035948 | 0.0056 |
| 200625_s_at | CAP1 | CAP, adenylate cyclase-associated protein 1 (yeast) | 10487 | 1.06 | 0.0361926 | 0.0077 |
| 229159_at | THSD7A | thrombospondin, type I, domain containing 7A | 221981 | 1.06 | 0.0428733 | 0.0078 |
| 206731_at | CNKSR2 | connector enhancer of kinase suppressor of Ras 2 | 22866 | 1.06 | 0.0434417 | 0.0084 |
| 243311_at | DEFB132 | defensin, beta 132 | 400830 | 1.06 | 0.0388388 | 0.0089 |
| 236563_at | RD3 | retinal degeneration 3 | 343035 | 1.06 | 0.03868 | 0.0109 |
| 215014_at | KCND3 | potassium voltage-gated channel, Shal-related subfamily, member 3 | 3752 | 1.06 | 0.04208 | 0.0117 |
| 243114_at | | | | 1.06 | 0.0400108 | 0.0134 |
| 1560153_at | FRAS1 | Fraser syndrome 1 | 80144 | 1.06 | 0.0428841 | 0.016 |
| 239539_at | | | | 1.06 | 0.0425079 | 0.0163 |
| 1557179_s_a | CARS2 | cysteinyl-tRNA synthetase 2, mitochondrial (putative) | 79587 | 1.06 | 0.04908 | 0.0184 |
| 213932_x_at | HLA-A | major histocompatibility complex, class I, A | 3105 | 1.05 | 0.0431689 | 0.0017 |
| 210920_x_at | | | | 0.94 | 0.02879 | 0.0017 |
| 238817_at | RIMBP2 | RIMS binding protein 2 | 23504 | 0.94 | 0.0431308 | 0.0078 |
| 209335_at | DCN | decorin | 1634 | 0.94 | 0.0330205 | 0.009 |

| ProbeSet | Symbol | Name | EntrezID | Fold Change | Parametric p-value | Permutation p-value |
|---|---|---|---|---|---|---|
| 232136_s_at | CTTNBP2 | cortactin binding protein 2 | 83992 | 0.94 | 0.0393377 | 0.0096 |
| 215958_at | | | | 0.94 | 0.0397152 | 0.0155 |
| 221910_at | ETV1 | ets variant 1 | 2115 | 0.94 | 0.0414769 | 0.0157 |
| 1552538_a_ | KIF6 | kinesin family member 6 | 221458 | 0.94 | 0.0362879 | 0.0164 |
| 1569693_at | BTBD8 | BTB (POZ) domain containing 8 | 284697 | 0.94 | 0.0374619 | 0.017 |
| 221632_s_at | WDR4 | WD repeat domain 4 | 10785 | 0.94 | 0.0419096 | 0.0173 |
| 237786_at | MAGI2 | membrane associated guanylate kinase, WW and PDZ domain containing 2 | 9863 | 0.94 | 0.0431187 | 0.0177 |
| 1565936_a_ | LMO3 | LIM domain only 3 (rhombotin-like 2) | 55885 | 0.94 | 0.0437591 | 0.0178 |
| 1556500_a_at | | | | 0.94 | 0.0398321 | 0.0179 |
| 1559546_s_at | | | | 0.94 | 0.043241 | 0.0194 |
| 221369_at | MTNR1A | melatonin receptor 1A | 4543 | 0.94 | 0.0495593 | 0.0204 |
| 237237_at | | | | 0.94 | 0.0414131 | 0.0218 |
| 211353_at | LRIT1 | leucine-rich repeat, immunoglobulin-like and transmembrane domains 1 | 26103 | 0.94 | 0.04461 | 0.0232 |
| 1569331_at | | | | 0.94 | 0.0452903 | 0.0275 |
| 237153_at | | | | 0.93 | 0.01783 | 0.0035 |
| 234387_at | COL4A5 | collagen, type IV, alpha 5 | 1287 | 0.93 | 0.0211845 | 0.0036 |
| 215120_s_at | SAMD4A | sterile alpha motif domain containing 4A | 23034 | 0.93 | 0.0184506 | 0.0048 |
| 237292_at | DPYSL3 | dihydropyrimidinase-like 3 | 1809 | 0.93 | 0.0195315 | 0.0049 |
| 1561224_at | | | | 0.93 | 0.0135087 | 0.0053 |
| 232446_at | MGC45800 | uncharacterized LOC90768 | 90768 | 0.93 | 0.0260552 | 0.0102 |
| 203902_at | HEPH | hephaestin | 9843 | 0.93 | 0.0222636 | 0.0115 |
| 237664_at | | | | 0.93 | 0.0267622 | 0.0118 |
| 233486_at | | | | 0.93 | 0.0242159 | 0.0119 |
| 208250_s_at | DMBT1 | deleted in malignant brain tumors 1 | 1755 | 0.93 | 0.0245978 | 0.0122 |
| 206724_at | CBX4 | chromobox homolog 4 | 8535 | 0.93 | 0.0289658 | 0.0124 |
| 208245_at | RAB9BP1 | RAB9B, member RAS oncogene family pseudogene 1 | 9366 | 0.93 | 0.0293017 | 0.0131 |
| 1569560_at | HIRA | HIR histone cell cycle regulation defective homolog A (S. cerevisiae) | 7290 | 0.93 | 0.0350531 | 0.0133 |
| 216307_at | DGKB | diacylglycerol kinase, beta 90kDa | 1607 | 0.93 | 0.0260956 | 0.0135 |
| 217376_at | LOC1002894 | cytoskeleton associated protein 2-like pseudogene | 100289473 | 0.93 | 0.0283762 | 0.0137 |
| 210310_s_at | FGF5 | fibroblast growth factor 5 | 2250 | 0.93 | 0.0266745 | 0.0148 |
| 216167_at | LRRN2 | leucine rich repeat neuronal 2 | 10446 | 0.93 | 0.0271956 | 0.0153 |
| 1560070_at | | | | 0.93 | 0.0293882 | 0.0153 |
| 234169_at | | | | 0.93 | 0.0368303 | 0.0165 |
| 215064_at | SC5DL | sterol-C5-desaturase (ERG3 delta-5-desaturase homolog, S. cerevisiae)-like | 6309 | 0.93 | 0.0316644 | 0.0166 |
| 201789_at | DHRS7 | dehydrogenase/reductase (SDR family) member 7 | 51635 | 0.93 | 0.0339341 | 0.0177 |
| 234308_at | TUBGCP6 | tubulin, gamma complex associated protein 6 | 85378 | 0.93 | 0.0351625 | 0.0179 |
| 1567272_at | OR2K2 | olfactory receptor, family 2, subfamily K, member 2 | 26248 | 0.93 | 0.0331112 | 0.0185 |
| 230642_at | | | | 0.93 | 0.0343278 | 0.0185 |
| 210435_at | | | | 0.93 | 0.0342506 | 0.0188 |
| 241247_at | | | | 0.93 | 0.0386636 | 0.0192 |
| 222897_s_at | ZFP64 | ZFP64 zinc finger protein | 55734 | 0.93 | 0.0337827 | 0.0205 |
| 214533_at | CMA1 | chymase 1, mast cell | 1215 | 0.93 | 0.0472124 | 0.0215 |
| 215658_at | LINC00675 | long intergenic non-protein coding RNA 675 | 100289255 | 0.93 | 0.0353787 | 0.0216 |
| 237550_at | | | | 0.93 | 0.0341813 | 0.0217 |
| 216159_s_at | | | | 0.93 | 0.0372907 | 0.0221 |
| 209846_s_at | BTN3A2 | butyrophilin, subfamily 3, member A2 | 11118 | 0.93 | 0.0445092 | 0.0221 |
| 220505_at | C9orf53 | chromosome 9 open reading frame 53 | 51198 | 0.93 | 0.0332022 | 0.0224 |
| 1554931_at | CYP4A11 | cytochrome P450, family 4, subfamily A, polypeptide 11 | 1579 | 0.93 | 0.0436811 | 0.0228 |
| 200081_s_at | RPS6 | ribosomal protein S6 | 6194 | 0.93 | 0.0372109 | 0.023 |
| 223667_at | FKBP7 | FK506 binding protein 7 | 51661 | 0.93 | 0.0429858 | 0.0253 |
| 237485_at | SRSF3 | serine/arginine-rich splicing factor 3 | 6428 | 0.93 | 0.0423849 | 0.0258 |
| 241400_at | | | | 0.93 | 0.0419887 | 0.0259 |
| 231159_at | | | | 0.93 | 0.0385926 | 0.0265 |
| 208153_s_at | FAT2 | FAT tumor suppressor homolog 2 (Drosophila) | 2196 | 0.93 | 0.0466871 | 0.0267 |
| 236225_at | GGT6 | gamma-glutamyltransferase 6 | 124975 | 0.93 | 0.0421057 | 0.0271 |
| 239270_at | PLCXD3 | phosphatidylinositol-specific phospholipase C, X domain containing 3 | 345557 | 0.93 | 0.0412514 | 0.0274 |
| 213001_at | ANGPTL2 | angiopoietin-like 2 | 23452 | 0.93 | 0.0389957 | 0.0276 |
| 229263_at | IL17RD | interleukin 17 receptor D | 54756 | 0.93 | 0.0429246 | 0.0276 |
| 211549_s_at | HPGD | hydroxyprostaglandin dehydrogenase 15-(NAD) | 3248 | 0.93 | 0.0432168 | 0.0277 |
| 210834_s_at | PTGER3 | prostaglandin E receptor 3 (subtype EP3) | 5733 | 0.93 | 0.0427571 | 0.0278 |
| 221165_s_at | IL22 | interleukin 22 | 50616 | 0.93 | 0.0399243 | 0.028 |
| 212389_at | SBF1 | SET binding factor 1 | 6305 | 0.93 | 0.0403413 | 0.028 |
| 243319_at | | | | 0.93 | 0.0443373 | 0.0283 |
| 211203_s_at | CNTN1 | contactin 1 | 1272 | 0.93 | 0.0469379 | 0.0286 |
| 1564385_at | LOC219688 | uncharacterized LOC219688 | 219688 | 0.93 | 0.039724 | 0.029 |
| 231586_at | SRG7 | spermatogenesis-related protein 7 | 642864 | 0.93 | 0.04364 | 0.0292 |
| 238430_x_at | SLFN5 | schlafen family member 5 | 162394 | 0.93 | 0.0455372 | 0.0294 |
| 1569508_at | PRDM5 | PR domain containing 5 | 11107 | 0.93 | 0.0447248 | 0.0303 |
| 208449_s_at | FGF8 | fibroblast growth factor 8 (androgen-induced) | 2253 | 0.93 | 0.045608 | 0.0307 |
| 1552295_a_ | SLC39A13 | solute carrier family 39 (zinc transporter), member 13 | 91252 | 0.93 | 0.0418867 | 0.0312 |
| 1562121_at | | | | 0.93 | 0.0406226 | 0.0315 |
| 212292_at | SLC7A1 | solute carrier family 7 (cationic amino acid transporter, y+ system), member 1 | 6541 | 0.93 | 0.0440038 | 0.032 |
| 1556014_at | MESP2 | mesoderm posterior 2 homolog (mouse) | 145873 | 0.93 | 0.046623 | 0.0327 |
| 1562623_at | LOC146513 | uncharacterized LOC146513 | 146513 | 0.93 | 0.048996 | 0.034 |
| 234453_s_at | C14orf166B | chromosome 14 open reading frame 166B | 145497 | 0.93 | 0.0492869 | 0.0354 |
| 236880_at | RAD52 | RAD52 homolog (S. cerevisiae) | 5893 | 0.93 | 0.047992 | 0.0355 |
| 234529_at | PCGEM1 | PCGEM1, prostate-specific transcript (non-protein coding) | 64002 | 0.93 | 0.0498309 | 0.0355 |
| 1559491_at | | | | 0.93 | 0.0466266 | 0.0373 |

| ProbeSet | Symbol | Name | EntrezID | Fold Change | Parametric p-value | Permutation p-value |
|---|---|---|---|---|---|---|
| 208487_at | LMX1B | LIM homeobox transcription factor 1, beta | 4010 | 0.93 | 0.0479041 | 0.0376 |
| 228503_at | RPS6KA6 | ribosomal protein S6 kinase, 90kDa, polypeptide 6 | 27330 | 0.92 | 0.0033371 | 2.00E-04 |
| 231354_at | LOC780529 | uncharacterized LOC780529 | 780529 | 0.92 | 0.0067314 | 0.001 |
| 218934_s_at | HSPB7 | heat shock 27kDa protein family, member 7 (cardiovascular) | 27129 | 0.92 | 0.0046837 | 0.0011 |
| 234636_at | | | | 0.92 | 0.0072332 | 0.0015 |
| 221371_at | TNFSF18 | tumor necrosis factor (ligand) superfamily, member 18 | 8995 | 0.92 | 0.0099676 | 0.0021 |
| 210562_at | GREB1 | growth regulation by estrogen in breast cancer 1 | 9687 | 0.92 | 0.0090703 | 0.0028 |
| 231740_at | KCNJ11 | potassium inwardly-rectifying channel, subfamily J, member 11 | 3767 | 0.92 | 0.0131937 | 0.004 |
| 1564387_at | DOPEY1 | dopey family member 1 | 23033 | 0.92 | 0.01345 | 0.0052 |
| 220197_at | ATP6V0A4 | ATPase, H+ transporting, lysosomal V0 subunit a4 | 50617 | 0.92 | 0.0115264 | 0.0057 |
| 242173_at | | | | 0.92 | 0.0129618 | 0.006 |
| 220593_s_at | CCDC40 | coiled-coil domain containing 40 | 55036 | 0.92 | 0.013842 | 0.0062 |
| 222384_at | SMG7-AS1 | SMG7 antisense RNA 1 | 284649 | 0.92 | 0.0132081 | 0.0073 |
| 234810_at | ZFP1 | ZFP1 zinc finger protein | 162239 | 0.92 | 0.014036 | 0.0089 |
| 242085_at | | | | 0.92 | 0.0142966 | 0.0095 |
| 224767_at | | | | 0.92 | 0.019202 | 0.0099 |
| 203510_at | MET | met proto-oncogene (hepatocyte growth factor receptor) | 4233 | 0.92 | 0.0199261 | 0.0107 |
| 1561584_at | | | | 0.92 | 0.0204234 | 0.0111 |
| 1566295_at | | | | 0.92 | 0.0210791 | 0.0114 |
| 233636_at | MIR17HG | miR-17-92 cluster host gene (non-protein coding) | 407975 | 0.92 | 0.0229594 | 0.0125 |
| 241078_at | SLC35E4 | solute carrier family 35, member E4 | 339665 | 0.92 | 0.0209537 | 0.0133 |
| 240083_at | MEG8 | maternally expressed 8 (non-protein coding) | 79104 | 0.92 | 0.0256194 | 0.0134 |
| 1556684_at | RPPH1 | ribonuclease P RNA component H1 | 85495 | 0.92 | 0.0181633 | 0.0135 |
| 235071_at | | | | 0.92 | 0.0185551 | 0.0137 |
| 1557620_a_a | CCDC38 | coiled-coil domain containing 38 | 120935 | 0.92 | 0.0247528 | 0.0137 |
| 204596_s_at | STC1 | stanniocalcin 1 | 6781 | 0.92 | 0.0227181 | 0.0138 |
| 208043_at | | | | 0.92 | 0.0226203 | 0.0154 |
| 211624_s_at | DRD2 | dopamine receptor D2 | 1813 | 0.92 | 0.0261984 | 0.0157 |
| 206996_x_at | CACNB1 | calcium channel, voltage-dependent, beta 1 subunit | 782 | 0.92 | 0.0282123 | 0.0157 |
| 1569659_at | LOC1005068 | uncharacterized LOC100506895 | 100506895 | 0.92 | 0.0264999 | 0.0162 |
| 1558577_at | LOC148709 | actin pseudogene | 148709 | 0.92 | 0.0241284 | 0.0166 |
| 237955_at | C5orf58 | chromosome 5 open reading frame 58 | 133874 | 0.92 | 0.027741 | 0.0166 |
| 217343_at | | | | 0.92 | 0.0270734 | 0.017 |
| 203441_s_at | CDH2 | cadherin 2, type 1, N-cadherin (neuronal) | 1000 | 0.92 | 0.0215654 | 0.0171 |
| 238240_at | | | | 0.92 | 0.0236454 | 0.0171 |
| 1563569_at | | | | 0.92 | 0.0273022 | 0.0176 |
| 1563822_at | LOC1001317 | uncharacterized LOC100131763 | 100131763 | 0.92 | 0.0273951 | 0.018 |
| 211942_x_at | | | | 0.92 | 0.0240226 | 0.0189 |
| 243610_at | C9orf135 | chromosome 9 open reading frame 135 | 138255 | 0.92 | 0.0254914 | 0.0189 |
| 217715_x_at | | | | 0.92 | 0.0262684 | 0.0193 |
| 1562689_at | LOC151484 | uncharacterized LOC151484 | 151484 | 0.92 | 0.0259196 | 0.02 |
| 1565612_at | DYNLRB1 | dynein, light chain, roadblock-type 1 | 83658 | 0.92 | 0.033162 | 0.0205 |
| 214351_x_at | | | | 0.92 | 0.0266921 | 0.0207 |
| 239961_at | | | | 0.92 | 0.0295116 | 0.021 |
| 234872_at | | | | 0.92 | 0.0280178 | 0.0211 |
| 214989_x_at | PLEKHA5 | pleckstrin homology domain containing, family A member 5 | 54477 | 0.92 | 0.0329443 | 0.0216 |
| 1569321_at | | | | 0.92 | 0.029428 | 0.0219 |
| 234711_s_at | BCORL1 | BCL6 corepressor-like 1 | 63035 | 0.92 | 0.0297177 | 0.022 |
| 231741_at | S1PR3 | sphingosine-1-phosphate receptor 3 | 1903 | 0.92 | 0.0327125 | 0.022 |
| 240589_at | LINC00313 | long intergenic non-protein coding RNA 313 | 114038 | 0.92 | 0.0321848 | 0.0223 |
| 210020_x_at | CALML3 | calmodulin-like 3 | 810 | 0.92 | 0.0315597 | 0.0224 |
| 230447_at | FAXC | failed axon connections homolog (Drosophila) | 84553 | 0.92 | 0.029776 | 0.0225 |
| 216340_s_at | CYP2A7P1 | cytochrome P450, family 2, subfamily A, polypeptide 7 pseudogene 1 | 1550 | 0.92 | 0.0306168 | 0.0228 |
| 1564282_a_a | LINC00491 | long intergenic non-protein coding RNA 491 | 285708 | 0.92 | 0.0319519 | 0.0235 |
| 1558765_a_at | | | | 0.92 | 0.0321542 | 0.0235 |
| 230624_at | SLC25A27 | solute carrier family 25, member 27 | 9481 | 0.92 | 0.0333265 | 0.0235 |
| 207479_at | | | | 0.92 | 0.0331916 | 0.0237 |
| 219984_s_at | HRASLS | HRAS-like suppressor | 57110 | 0.92 | 0.0323433 | 0.024 |
| 231534_at | CDK1 | cyclin-dependent kinase 1 | 983 | 0.92 | 0.032699 | 0.0242 |
| 206661_at | DBF4B | DBF4 homolog B (S. cerevisiae) | 80174 | 0.92 | 0.0310493 | 0.0243 |
| 205586_x_at | VGF | VGF nerve growth factor inducible | 7425 | 0.92 | 0.03001 | 0.0245 |
| 241642_x_at | TLK1 | tousled-like kinase 1 | 9874 | 0.92 | 0.0361401 | 0.0253 |
| 235650_at | CDHR3 | cadherin-related family member 3 | 222256 | 0.92 | 0.0305768 | 0.0257 |
| 1557031_at | RNF212 | ring finger protein 212 | 285498 | 0.92 | 0.0309782 | 0.0257 |
| 233206_at | SLC22A23 | solute carrier family 22, member 23 | 63027 | 0.92 | 0.0333116 | 0.0259 |
| 224244_s_at | APOA5 | apolipoprotein A-V | 116519 | 0.92 | 0.0319841 | 0.0262 |
| 241267_at | EHD3 | EH-domain containing 3 | 30845 | 0.92 | 0.0348774 | 0.0266 |
| 220437_at | C19orf80 | chromosome 19 open reading frame 80 | 55908 | 0.92 | 0.0369108 | 0.0268 |
| 242260_at | MATR3 | matrin 3 | 9782 | 0.92 | 0.0351966 | 0.0269 |
| 1570200_at | HELB | helicase (DNA) B | 92797 | 0.92 | 0.0404471 | 0.0275 |
| 237986_at | | | | 0.92 | 0.0330764 | 0.0276 |
| 205319_at | PSCA | prostate stem cell antigen | 8000 | 0.92 | 0.0391696 | 0.0277 |
| 224499_s_at | AICDA | activation-induced cytidine deaminase | 57379 | 0.92 | 0.0344984 | 0.0282 |
| 227345_at | TNFRSF10D | tumor necrosis factor receptor superfamily, member 10d, decoy with truncated death domain | 8793 | 0.92 | 0.0345689 | 0.0282 |
| 240942_at | MPHOSPH8 | M-phase phosphoprotein 8 | 54737 | 0.92 | 0.0371343 | 0.0289 |
| 207444_at | SLC22A13 | solute carrier family 22 (organic anion transporter), member 13 | 9390 | 0.92 | 0.0369954 | 0.0292 |
| 233607_at | | | | 0.92 | 0.0378195 | 0.0294 |
| 1569009_s_at | | | | 0.92 | 0.0336635 | 0.0296 |

| ProbeSet | Symbol | Name | EntrezID | Fold Change | Parametric p-value | Permutation p-value |
|---|---|---|---|---|---|---|
| 1558167_a_a | MGC16275 | uncharacterized protein MGC16275 | 85001 | 0.92 | 0.0373603 | 0.0296 |
| 1561266_at | LOC1005070 | uncharacterized LOC100507033 | 100507033 | 0.92 | 0.0368698 | 0.0308 |
| 243425_at | | | | 0.92 | 0.039604 | 0.0308 |
| 1553234_at | ADAMTS18 | ADAM metallopeptidase with thrombospondin type 1 motif, 18 | 170692 | 0.92 | 0.0389834 | 0.0318 |
| 204723_at | SCN3B | sodium channel, voltage-gated, type III, beta subunit | 55800 | 0.92 | 0.0396902 | 0.0321 |
| 220454_s_at | SEMA6A | sema domain, transmembrane domain (TM), and cytoplasmic domain, (semaphorin) 6A | 57556 | 0.92 | 0.035372 | 0.0322 |
| 205465_x_at | HS3ST1 | heparan sulfate (glucosamine) 3-O-sulfotransferase 1 | 9957 | 0.92 | 0.0416073 | 0.0324 |
| 217580_x_at | | | | 0.92 | 0.0363874 | 0.0328 |
| 242959_at | | | | 0.92 | 0.040069 | 0.0331 |
| 234959_at | | | | 0.92 | 0.0422952 | 0.0333 |
| 209646_x_at | ALDH1B1 | aldehyde dehydrogenase 1 family, member B1 | 219 | 0.92 | 0.0433499 | 0.0333 |
| 221325_at | KCNK13 | potassium channel, subfamily K, member 13 | 56659 | 0.92 | 0.040692 | 0.0338 |
| 204021_s_at | PURA | purine-rich element binding protein A | 5813 | 0.92 | 0.0395535 | 0.0341 |
| 240534_at | | | | 0.92 | 0.039819 | 0.0341 |
| 1553089_a_a | WFDC2 | WAP four-disulfide core domain 2 | 10406 | 0.92 | 0.0438567 | 0.0347 |
| 1559174_at | RAB6A | RAB6A, member RAS oncogene family | 5870 | 0.92 | 0.0406348 | 0.0358 |
| 233617_at | TSPY26P | testis specific protein, Y-linked 26, pseudogene | 128854 | 0.92 | 0.044821 | 0.0363 |
| 243917_at | CLIC5 | chloride intracellular channel 5 | 53405 | 0.92 | 0.0433521 | 0.0366 |
| 1569272_at | PIK3C3 | phosphatidylinositol 3-kinase, catalytic subunit type 3 | 5289 | 0.92 | 0.0458204 | 0.0368 |
| 202882_x_at | NOL7 | nucleolar protein 7, 27kDa | 51406 | 0.92 | 0.0427871 | 0.0375 |
| 238292_at | | | | 0.92 | 0.0428303 | 0.0378 |
| 1556231_a_at | | | | 0.92 | 0.0458668 | 0.0378 |
| 1559796_at | EBF3 | early B-cell factor 3 | 253738 | 0.92 | 0.0399471 | 0.0379 |
| 207490_at | TUBA4B | tubulin, alpha 4b (pseudogene) | 80086 | 0.92 | 0.0467291 | 0.038 |
| 208324_at | AKAP13 | A kinase (PRKA) anchor protein 13 | 11214 | 0.92 | 0.0429942 | 0.0382 |
| 237959_at | | | | 0.92 | 0.0451196 | 0.0383 |
| 208157_at | SIM2 | single-minded homolog 2 (Drosophila) | 6493 | 0.92 | 0.0380331 | 0.0386 |
| 1563149_at | | | | 0.92 | 0.0430486 | 0.0386 |
| 219932_at | SLC27A6 | solute carrier family 27 (fatty acid transporter), member 6 | 28965 | 0.92 | 0.046072 | 0.0386 |
| 1560917_at | | | | 0.92 | 0.0498682 | 0.0387 |
| 225901_at | PTPMT1 | protein tyrosine phosphatase, mitochondrial 1 | 114971 | 0.92 | 0.0441444 | 0.0388 |
| 238099_at | | | | 0.92 | 0.0470499 | 0.0389 |
| 204939_s_at | PLN | phospholamban | 5350 | 0.92 | 0.0426111 | 0.0403 |
| 227779_at | ECSCR | endothelial cell surface expressed chemotaxis and apoptosis regulator | 641700 | 0.92 | 0.0471052 | 0.0405 |
| 1559795_at | | | | 0.92 | 0.0439514 | 0.0413 |
| 230910_s_at | | | | 0.92 | 0.045141 | 0.0413 |
| 220188_at | JPH3 | junctophilin 3 | 57338 | 0.92 | 0.0498056 | 0.0416 |
| 228635_at | PCDH10 | protocadherin 10 | 57575 | 0.92 | 0.0438593 | 0.0417 |
| 233171_at | GRIN3A | glutamate receptor, ionotropic, N-methyl-D-aspartate 3A | 116443 | 0.92 | 0.0466057 | 0.0417 |
| 235941_s_at | | | | 0.92 | 0.0464568 | 0.0419 |
| 1554615_at | | | | 0.92 | 0.0477233 | 0.0425 |
| 1555141_a_a | SLC9B1 | solute carrier family 9, subfamily B (NHA1, cation proton antiporter 1), member 1 | 150159 | 0.92 | 0.0490843 | 0.0431 |
| 235335_at | ABCA9 | ATP-binding cassette, sub-family A (ABC1), member 9 | 10350 | 0.92 | 0.0471133 | 0.0437 |
| 229199_at | SCN9A | sodium channel, voltage-gated, type IX, alpha subunit | 6335 | 0.92 | 0.0447624 | 0.044 |
| 226679_at | SLC26A11 | solute carrier family 26, member 11 | 284129 | 0.92 | 0.0469891 | 0.0448 |
| 243769_at | | | | 0.92 | 0.0469324 | 0.0449 |
| 1555462_at | PPP1R1C | protein phosphatase 1, regulatory (inhibitor) subunit 1C | 151242 | 0.92 | 0.0475985 | 0.0464 |
| 210263_at | KCNF1 | potassium voltage-gated channel, subfamily F, member 1 | 3754 | 0.92 | 0.0479089 | 0.0466 |
| 218731_s_at | VWA1 | von Willebrand factor A domain containing 1 | 64856 | 0.92 | 0.0494334 | 0.0476 |
| 223813_at | PRND | prion protein 2 (dublet) | 23627 | 0.92 | 0.0489518 | 0.0488 |
| 237214_at | TFRC | transferrin receptor (p90, CD71) | 7037 | 0.91 | 0.0026235 | 0.0011 |
| 1556920_s_a | LOC646168 | uncharacterized LOC646168 | 646168 | 0.91 | 0.0064641 | 0.0023 |
| 1570474_s_a | ANKRD30BP | ankyrin repeat domain 30B pseudogene 3 | 338579 | 0.91 | 0.0092849 | 0.0047 |
| 204422_s_at | FGF2 | fibroblast growth factor 2 (basic) | 2247 | 0.91 | 0.0091464 | 0.0053 |
| 1555083_at | RPL13AP17 | ribosomal protein L13a pseudogene 17 | 399670 | 0.91 | 0.0159679 | 0.0069 |
| 234442_at | | | | 0.91 | 0.011381 | 0.0074 |
| 1568635_at | | | | 0.91 | 0.0123884 | 0.0078 |
| 221259_s_at | TEX11 | testis expressed 11 | 56159 | 0.91 | 0.0092517 | 0.0079 |
| 237711_at | ZNF705G | zinc finger protein 705G | 100131980 | 0.91 | 0.014009 | 0.008 |
| 231559_at | NNMT | nicotinamide N-methyltransferase | 4837 | 0.91 | 0.0166315 | 0.0082 |
| 235044_at | CYYR1 | cysteine/tyrosine-rich 1 | 116159 | 0.91 | 0.013524 | 0.0083 |
| 238858_at | TIFA | TRAF-interacting protein with forkhead-associated domain | 92610 | 0.91 | 0.0146625 | 0.0097 |
| 1565795_at | DUOX1 | dual oxidase 1 | 53905 | 0.91 | 0.0139249 | 0.0101 |
| 235815_at | TSHZ2 | teashirt zinc finger homeobox 2 | 128553 | 0.91 | 0.0158219 | 0.0103 |
| 1553298_at | C17orf77 | chromosome 17 open reading frame 77 | 146723 | 0.91 | 0.0153907 | 0.0107 |
| 1560921_at | ZNF169 | zinc finger protein 169 | 169841 | 0.91 | 0.0140135 | 0.011 |
| 226926_at | DMKN | dermokine | 93099 | 0.91 | 0.0164615 | 0.011 |
| 243901_at | ALDOB | aldolase B, fructose-bisphosphate | 229 | 0.91 | 0.0168483 | 0.0125 |
| 219533_at | CDKN1C | cyclin-dependent kinase inhibitor 1C (p57, Kip2) | 1028 | 0.91 | 0.0174308 | 0.0128 |
| 225867_at | VASN | vasorin | 114990 | 0.91 | 0.0190429 | 0.0129 |
| 231327_at | | | | 0.91 | 0.0192487 | 0.0131 |
| 1555774_at | ZAR1 | zygote arrest 1 | 326340 | 0.91 | 0.0207893 | 0.0133 |
| 240784_at | NAT16 | N-acetyltransferase 16 (GCN5-related, putative) | 375607 | 0.91 | 0.0192727 | 0.0135 |
| 233414_at | | | | 0.91 | 0.0161482 | 0.0136 |
| 1561899_at | CLECL1 | C-type lectin-like 1 | 160365 | 0.91 | 0.0151193 | 0.0147 |
| 1559672_a_a | CCDC171 | coiled-coil domain containing 171 | 203238 | 0.91 | 0.0197016 | 0.0158 |
| 223715_at | BRSK2 | BR serine/threonine kinase 2 | 9024 | 0.91 | 0.0173008 | 0.016 |
| 229301_at | TUG1 | taurine upregulated 1 (non-protein coding) | 55000 | 0.91 | 0.0214239 | 0.0163 |

| ProbeSet | Symbol | Name | EntrezID | Fold Change | Parametric p-value | Permutation p-value |
|---|---|---|---|---|---|---|
| 1552280_at | TIMD4 | T-cell immunoglobulin and mucin domain containing 4 | 91937 | 0.91 | 0.0193015 | 0.0164 |
| 1561311_at | | | | 0.91 | 0.0213474 | 0.0169 |
| 244105_at | | | | 0.91 | 0.0226942 | 0.0177 |
| 232712_at | | | | 0.91 | 0.023325 | 0.0178 |
| 217217_at | | | | 0.91 | 0.0211694 | 0.0182 |
| 1556941_a_a | LOC283484 | uncharacterized LOC283484 | 283484 | 0.91 | 0.0198383 | 0.0183 |
| 240144_at | DNASE1 | deoxyribonuclease I | 1773 | 0.91 | 0.0223135 | 0.0188 |
| 216139_s_at | MAPK8IP3 | mitogen-activated protein kinase 8 interacting protein 3 | 23162 | 0.91 | 0.031311 | 0.0192 |
| 205345_at | BARD1 | BRCA1 associated RING domain 1 | 580 | 0.91 | 0.0221148 | 0.0199 |
| 230677_at | EXOC3L4 | exocyst complex component 3-like 4 | 91828 | 0.91 | 0.0204594 | 0.0201 |
| 244404_at | STXBP4 | syntaxin binding protein 4 | 252983 | 0.91 | 0.0269161 | 0.0208 |
| 227886_at | IFITM10 | interferon induced transmembrane protein 10 | 402778 | 0.91 | 0.0239199 | 0.0211 |
| 233564_s_at | CDADC1 | cytidine and dCMP deaminase domain containing 1 | 81602 | 0.91 | 0.0255629 | 0.0215 |
| 231849_at | KRT80 | keratin 80 | 144501 | 0.91 | 0.0247135 | 0.0216 |
| 214932_at | KIDINS220 | kinase D-interacting substrate, 220kDa | 57498 | 0.91 | 0.025308 | 0.0216 |
| 228166_at | ALG2 | ALG2, alpha-1,3/1,6-mannosyltransferase | 85365 | 0.91 | 0.0254307 | 0.0216 |
| 211174_s_at | CCKAR | cholecystokinin A receptor | 886 | 0.91 | 0.0260822 | 0.022 |
| 1561461_at | | | | 0.91 | 0.0264457 | 0.0222 |
| 1560148_at | MIA3 | melanoma inhibitory activity family, member 3 | 375056 | 0.91 | 0.0240841 | 0.0223 |
| 231807_at | KIAA1217 | KIAA1217 | 56243 | 0.91 | 0.0300272 | 0.0224 |
| 1554961_at | FGFR4 | fibroblast growth factor receptor 4 | 2264 | 0.91 | 0.026814 | 0.0227 |
| 1560059_at | VPS37C | vacuolar protein sorting 37 homolog C (S. cerevisiae) | 55048 | 0.91 | 0.0262998 | 0.0229 |
| 237704_at | | | | 0.91 | 0.0280777 | 0.0236 |
| 1554766_s_at | | | | 0.91 | 0.0264378 | 0.0237 |
| 1559561_at | FBXO18 | F-box protein, helicase, 18 | 84893 | 0.91 | 0.0294291 | 0.0238 |
| 244459_at | | | | 0.91 | 0.0238978 | 0.0241 |
| 237564_at | | | | 0.91 | 0.0271545 | 0.0244 |
| 233061_at | FITM2 | fat storage-inducing transmembrane protein 2 | 128486 | 0.91 | 0.0274844 | 0.025 |
| 1554163_at | TWIST2 | twist basic helix-loop-helix transcription factor 2 | 117581 | 0.91 | 0.0312144 | 0.0251 |
| 227443_at | LURAP1L | leucine rich adaptor protein 1-like | 286343 | 0.91 | 0.0277984 | 0.0253 |
| 231539_at | | | | 0.91 | 0.0294587 | 0.0255 |
| 238295_at | TEFM | transcription elongation factor, mitochondrial | 79736 | 0.91 | 0.0271288 | 0.0257 |
| 207043_s_at | SLC6A9 | solute carrier family 6 (neurotransmitter transporter, glycine), member 9 | 6536 | 0.91 | 0.0282983 | 0.0261 |
| 218990_s_at | SPRR3 | small proline-rich protein 3 | 6707 | 0.91 | 0.0303594 | 0.0262 |
| 208553_at | HIST1H1E | histone cluster 1, H1e | 3008 | 0.91 | 0.0304313 | 0.0263 |
| 233353_at | FER1L5 | fer-1-like 5 (C. elegans) | 90342 | 0.91 | 0.0249778 | 0.0268 |
| 221347_at | CHRM5 | cholinergic receptor, muscarinic 5 | 1133 | 0.91 | 0.0334029 | 0.027 |
| 1552466_x_a | LINC00161 | long intergenic non-protein coding RNA 161 | 118421 | 0.91 | 0.0308221 | 0.0272 |
| 240272_at | | | | 0.91 | 0.0279865 | 0.0274 |
| 215807_s_at | PLXNB1 | plexin B1 | 5364 | 0.91 | 0.027674 | 0.0275 |
| 241065_x_at | CMAS | cytidine monophosphate N-acetylneuraminic acid synthetase | 55907 | 0.91 | 0.0323852 | 0.0276 |
| 233513_at | | | | 0.91 | 0.0312465 | 0.028 |
| 203368_at | CRELD1 | cysteine-rich with EGF-like domains 1 | 78987 | 0.91 | 0.0318701 | 0.0281 |
| 217123_x_at | PMCHL1 | pro-melanin-concentrating hormone-like 1, pseudogene | 5369 | 0.91 | 0.0279282 | 0.0284 |
| 1560559_at | | | | 0.91 | 0.0293025 | 0.0284 |
| 209983_s_at | NRXN2 | neurexin 2 | 9379 | 0.91 | 0.0293124 | 0.0287 |
| 243983_at | | | | 0.91 | 0.0309528 | 0.0287 |
| 241596_at | NUDT10 | nudix (nucleoside diphosphate linked moiety X)-type motif 10 | 170685 | 0.91 | 0.0333235 | 0.029 |
| 238882_at | | | | 0.91 | 0.0327586 | 0.0298 |
| 212783_at | RBBP6 | retinoblastoma binding protein 6 | 5930 | 0.91 | 0.0298002 | 0.0301 |
| 205677_s_at | DLEU1 | deleted in lymphocytic leukemia 1 (non-protein coding) | 10301 | 0.91 | 0.0284363 | 0.0302 |
| 206230_at | LHX1 | LIM homeobox 1 | 3975 | 0.91 | 0.0297258 | 0.0302 |
| 223605_at | SLC25A18 | solute carrier family 25 (glutamate carrier), member 18 | 83733 | 0.91 | 0.025165 | 0.0304 |
| 204813_at | MAPK10 | mitogen-activated protein kinase 10 | 5602 | 0.91 | 0.031982 | 0.0308 |
| 214354_x_at | SFTPB | surfactant protein B | 6439 | 0.91 | 0.0330017 | 0.0312 |
| 230583_s_at | | | | 0.91 | 0.0370785 | 0.0321 |
| 231459_at | | | | 0.91 | 0.034374 | 0.0324 |
| 215068_s_at | FBXL18 | F-box and leucine-rich repeat protein 18 | 80028 | 0.91 | 0.0298534 | 0.0335 |
| 208458_at | SCNN1D | sodium channel, non-voltage-gated 1, delta subunit | 6339 | 0.91 | 0.0348876 | 0.0335 |
| 1560385_x_at | | | | 0.91 | 0.0325906 | 0.0339 |
| 1562849_at | | | | 0.91 | 0.0344802 | 0.034 |
| 1558334_a_a | C22orf15 | chromosome 22 open reading frame 15 | 150248 | 0.91 | 0.0319244 | 0.0344 |
| 1561990_at | LOC157931 | uncharacterized LOC157931 | 157931 | 0.91 | 0.0345994 | 0.0344 |
| 1557795_s_a | NTRK3 | neurotrophic tyrosine kinase, receptor, type 3 | 4916 | 0.91 | 0.0363302 | 0.0347 |
| 211181_x_at | | | | 0.91 | 0.0374726 | 0.0348 |
| 1560615_a_at | | | | 0.91 | 0.0356346 | 0.0357 |
| 206816_s_at | SPAG8 | sperm associated antigen 8 | 26206 | 0.91 | 0.0319614 | 0.0358 |
| 244862_at | | | | 0.91 | 0.0355075 | 0.0367 |
| 1554842_at | SLC12A1 | solute carrier family 12 (sodium/potassium/chloride transporters), member 1 | 6557 | 0.91 | 0.0355587 | 0.0375 |
| 207311_at | DOC2B | double C2-like domains, beta | 8447 | 0.91 | 0.037759 | 0.038 |
| 236903_at | | | | 0.91 | 0.0402142 | 0.0383 |
| 206094_x_at | | | | 0.91 | 0.0385487 | 0.0394 |
| 222222_s_at | HOMER3 | homer homolog 3 (Drosophila) | 9454 | 0.91 | 0.0400935 | 0.0394 |
| 201283_s_at | TRAK1 | trafficking protein, kinesin binding 1 | 22906 | 0.91 | 0.0440508 | 0.0395 |
| 1554379_a_a | TP73 | tumor protein p73 | 7161 | 0.91 | 0.039624 | 0.0398 |
| 1557382_x_a | AGAP11 | ankyrin repeat and GTPase domain Arf GTPase activating protein 11 | 119385 | 0.91 | 0.0402965 | 0.04 |
| 239106_at | | | | 0.91 | 0.0362135 | 0.0401 |
| 211277_x_at | APP | amyloid beta (A4) precursor protein | 351 | 0.91 | 0.0374305 | 0.0401 |

| ProbeSet | Symbol | Name | EntrezID | Fold Change | Parametric p-value | Permutation p-value |
|---|---|---|---|---|---|---|
| 217652_at | MAU2 | MAU2 chromatid cohesion factor homolog (C. elegans) | 23383 | 0.91 | 0.0404588 | 0.0401 |
| 216990_at | GART | phosphoribosylglycinamide formyltransferase, phosphoribosylglycinamide synthetase, phosphoribos | 2618 | 0.91 | 0.0384667 | 0.0402 |
| 217504_at | ABCA6 | ATP-binding cassette, sub-family A (ABC1), member 6 | 23460 | 0.91 | 0.0429977 | 0.0402 |
| 210925_at | CIITA | class II, major histocompatibility complex, transactivator | 4261 | 0.91 | 0.0384839 | 0.0406 |
| 1553388_at | FAM26D | family with sequence similarity 26, member D | 221301 | 0.91 | 0.0362013 | 0.0407 |
| 219507_at | RSRC1 | arginine/serine-rich coiled-coil 1 | 51319 | 0.91 | 0.0386687 | 0.0407 |
| 1553523_at | NLRP14 | NLR family, pyrin domain containing 14 | 338323 | 0.91 | 0.038239 | 0.0408 |
| 238664_s_at | MGC12916 | uncharacterized protein MGC12916 | 84815 | 0.91 | 0.0400527 | 0.0408 |
| 232124_at | FAM198A | family with sequence similarity 198, member A | 729085 | 0.91 | 0.0418569 | 0.041 |
| 217136_at | | | | 0.91 | 0.0390999 | 0.0417 |
| 1553995_a_a | NT5E | 5'-nucleotidase, ecto (CD73) | 4907 | 0.91 | 0.038353 | 0.042 |
| 1565016_at | PRMT1 | protein arginine methyltransferase 1 | 3276 | 0.91 | 0.041456 | 0.0422 |
| 212018_s_at | RSL1D1 | ribosomal L1 domain containing 1 | 26156 | 0.91 | 0.0377566 | 0.0423 |
| 221553_at | MAGT1 | magnesium transporter 1 | 84061 | 0.91 | 0.0402131 | 0.0425 |
| 1557158_s_a | MLL3 | myeloid/lymphoid or mixed-lineage leukemia 3 | 58508 | 0.91 | 0.0470905 | 0.0428 |
| 232083_at | KIF16B | kinesin family member 16B | 55614 | 0.91 | 0.0401279 | 0.0429 |
| 230523_at | QSOX1 | quiescin Q6 sulfhydryl oxidase 1 | 5768 | 0.91 | 0.0419233 | 0.0432 |
| 234468_at | | | | 0.91 | 0.0437859 | 0.0441 |
| 226446_at | HES6 | hairy and enhancer of split 6 (Drosophila) | 55502 | 0.91 | 0.0419541 | 0.0442 |
| 240073_at | | | | 0.91 | 0.0422361 | 0.0445 |
| 227262_at | HAPLN3 | hyaluronan and proteoglycan link protein 3 | 145864 | 0.91 | 0.0467983 | 0.0458 |
| 217373_x_at | MDM2 | Mdm2, p53 E3 ubiquitin protein ligase homolog (mouse) | 4193 | 0.91 | 0.0433893 | 0.0459 |
| 241303_x_at | | | | 0.91 | 0.0426222 | 0.0461 |
| 1554847_at | ATP6V1B1 | ATPase, H+ transporting, lysosomal 56/58kDa, V1 subunit B1 | 525 | 0.91 | 0.0496983 | 0.0467 |
| 216839_at | LAMA2 | laminin, alpha 2 | 3908 | 0.91 | 0.043638 | 0.0472 |
| 234271_at | OTOP2 | otopetrin 2 | 92736 | 0.91 | 0.0454529 | 0.0478 |
| 1555321_at | ACOT11 | acyl-CoA thioesterase 11 | 26027 | 0.91 | 0.0480408 | 0.0481 |
| 1553248_at | CCDC57 | coiled-coil domain containing 57 | 284001 | 0.91 | 0.0426734 | 0.049 |
| 222344_at | | | | 0.9 | 0.0024546 | 5.00E-04 |
| 1552318_at | GIMAP1 | GTPase, IMAP family member 1 | 170575 | 0.9 | 0.0029988 | 0.0017 |
| 1566586_at | | | | 0.9 | 0.0048306 | 0.0021 |
| 229708_at | TOR4A | torsin family 4, member A | 54863 | 0.9 | 0.0039889 | 0.0028 |
| 204969_s_at | RDX | radixin | 5962 | 0.9 | 0.004246 | 0.0029 |
| 1561978_at | LOC284798 | uncharacterized LOC284798 | 284798 | 0.9 | 0.006317 | 0.0032 |
| 1569724_at | | | | 0.9 | 0.0060279 | 0.0037 |
| 216296_at | CLTA | clathrin, light chain A | 1211 | 0.9 | 0.0112425 | 0.005 |
| 1558160_at | CCDC171 | coiled-coil domain containing 171 | 203238 | 0.9 | 0.008216 | 0.0052 |
| 1561964_at | | | | 0.9 | 0.0087656 | 0.0055 |
| 219762_s_at | RPL36 | ribosomal protein L36 | 25873 | 0.9 | 0.0067977 | 0.0056 |
| 236479_at | | | | 0.9 | 0.0086621 | 0.0064 |
| 206837_at | ALX1 | ALX homeobox 1 | 8092 | 0.9 | 0.0071878 | 0.0066 |
| 232642_at | VWA5B2 | von Willebrand factor A domain containing 5B2 | 90113 | 0.9 | 0.0086608 | 0.0067 |
| 203294_s_at | LMAN1 | lectin, mannose-binding, 1 | 3998 | 0.9 | 0.0081208 | 0.0068 |
| 239039_at | | | | 0.9 | 0.0088813 | 0.0069 |
| 227690_at | GABRB3 | gamma-aminobutyric acid (GABA) A receptor, beta 3 | 2562 | 0.9 | 0.0090485 | 0.007 |
| 1560097_at | FHAD1 | forkhead-associated (FHA) phosphopeptide binding domain 1 | 114827 | 0.9 | 0.0086792 | 0.0072 |
| 1557190_at | | | | 0.9 | 0.0108274 | 0.0073 |
| 1555587_at | PDZRN3 | PDZ domain containing ring finger 3 | 23024 | 0.9 | 0.0116679 | 0.0076 |
| 1561307_at | | | | 0.9 | 0.0088451 | 0.0082 |
| 216090_x_at | | | | 0.9 | 0.0105077 | 0.0084 |
| 235713_at | ALKBH8 | alkB, alkylation repair homolog 8 (E. coli) | 91801 | 0.9 | 0.0120109 | 0.0085 |
| 1569235_a_at | | | | 0.9 | 0.0103639 | 0.0094 |
| 217444_at | | | | 0.9 | 0.0123765 | 0.0095 |
| 1556844_at | LOC1001320 | uncharacterized LOC100132078 | 100132078 | 0.9 | 0.0116755 | 0.0096 |
| 214154_s_at | PKP2 | plakophilin 2 | 5318 | 0.9 | 0.0097454 | 0.0105 |
| 1559591_s_a | CHDH | choline dehydrogenase | 55349 | 0.9 | 0.010929 | 0.0108 |
| 229239_x_at | SLCO4A1 | solute carrier organic anion transporter family, member 4A1 | 28231 | 0.9 | 0.011815 | 0.0109 |
| 215547_at | TSC22D2 | TSC22 domain family, member 2 | 9819 | 0.9 | 0.0121222 | 0.0112 |
| 235586_at | | | | 0.9 | 0.0136842 | 0.0115 |
| 207978_s_at | NR4A3 | nuclear receptor subfamily 4, group A, member 3 | 8013 | 0.9 | 0.0138058 | 0.0118 |
| 226216_at | INSR | insulin receptor | 3643 | 0.9 | 0.0147646 | 0.0127 |
| 223579_s_at | APOB | apolipoprotein B (including Ag(x) antigen) | 338 | 0.9 | 0.0113263 | 0.0129 |
| 1562786_at | | | | 0.9 | 0.0131939 | 0.0134 |
| 217021_at | CYB5A | cytochrome b5 type A (microsomal) | 1528 | 0.9 | 0.014659 | 0.0134 |
| 1564784_at | | | | 0.9 | 0.0115386 | 0.0138 |
| 235017_s_at | CSRNP3 | cysteine-serine-rich nuclear protein 3 | 80034 | 0.9 | 0.0145179 | 0.0141 |
| 1559324_at | USP32P2 | ubiquitin specific peptidase 32 pseudogene 2 | 220594 | 0.9 | 0.0142771 | 0.0144 |
| 1561144_at | | | | 0.9 | 0.0178301 | 0.0146 |
| 1563082_at | LINC00486 | long intergenic non-protein coding RNA 486 | 285045 | 0.9 | 0.0148947 | 0.0149 |
| 242683_at | LINC00668 | long intergenic non-protein coding RNA 668 | 400643 | 0.9 | 0.015019 | 0.0149 |
| 207653_at | FOXD2 | forkhead box D2 | 2306 | 0.9 | 0.0164979 | 0.0158 |
| 236434_at | PES1 | pescadillo ribosomal biogenesis factor 1 | 23481 | 0.9 | 0.0172565 | 0.0158 |
| 241411_at | | | | 0.9 | 0.016517 | 0.0159 |
| 1570212_a_a | GNN | Grp94 neighboring nucleotidase pseudogene | 253724 | 0.9 | 0.019704 | 0.0164 |
| 1552573_s_a | MIPOL1 | mirror-image polydactyly 1 | 145282 | 0.9 | 0.0200862 | 0.0173 |
| 240576_at | | | | 0.9 | 0.0219995 | 0.0176 |
| 222300_at | | | | 0.9 | 0.0197296 | 0.018 |
| 233950_at | CADPS | Ca++-dependent secretion activator | 8618 | 0.9 | 0.0206382 | 0.018 |

| ProbeSet | Symbol | Name | EntrezID | Fold Change | Parametric p-value | Permutation p-value |
|---|---|---|---|---|---|---|
| 219888_at | SPAG4 | sperm associated antigen 4 | 6676 | 0.9 | 0.0183598 | 0.0193 |
| 1562922_at | | | | 0.9 | 0.019108 | 0.0198 |
| 214499_s_at | BCLAF1 | BCL2-associated transcription factor 1 | 9774 | 0.9 | 0.0189279 | 0.0199 |
| 242115_at | | | | 0.9 | 0.0211668 | 0.02 |
| 1564070_s_a | HOTTIP | HOXA distal transcript antisense RNA | 100316868 | 0.9 | 0.0189333 | 0.0202 |
| 243468_at | PATE1 | prostate and testis expressed 1 | 160065 | 0.9 | 0.0201843 | 0.0207 |
| 228169_s_at | HES6 | hairy and enhancer of split 6 (Drosophila) | 55502 | 0.9 | 0.0201722 | 0.0209 |
| 244864_at | | | | 0.9 | 0.0225695 | 0.0209 |
| 220589_s_at | | | | 0.9 | 0.0197347 | 0.021 |
| 233828_at | | | | 0.9 | 0.0211874 | 0.022 |
| 226900_at | | | | 0.9 | 0.0211985 | 0.0222 |
| 223079_s_at | GLS | glutaminase | 2744 | 0.9 | 0.0238503 | 0.0222 |
| 219419_at | RBFA | ribosome binding factor A (putative) | 79863 | 0.9 | 0.0201871 | 0.0224 |
| 207692_s_at | ACAN | aggrecan | 176 | 0.9 | 0.0212748 | 0.0232 |
| 241112_at | ZNF517 | zinc finger protein 517 | 340385 | 0.9 | 0.0235084 | 0.0242 |
| 224258_at | DBIL5P2 | diazepam binding inhibitor-like 5 pseudogene 2 | 100169989 | 0.9 | 0.0236173 | 0.0255 |
| 231531_at | FANCD2OS | FANCD2 opposite strand | 115795 | 0.9 | 0.024789 | 0.0263 |
| 215604_x_at | | | | 0.9 | 0.0274651 | 0.0265 |
| 1564653_s_a | LEKR1 | leucine, glutamate and lysine rich 1 | 389170 | 0.9 | 0.0289898 | 0.0266 |
| 1555145_at | CCSAP | centriole, cilia and spindle-associated protein | 126731 | 0.9 | 0.0251619 | 0.0272 |
| 1569212_at | LOC619207 | scavenger receptor protein family member | 619207 | 0.9 | 0.0325295 | 0.0278 |
| 204403_x_at | | | | 0.9 | 0.026037 | 0.0281 |
| 234194_at | | | | 0.9 | 0.0381586 | 0.0282 |
| 240553_at | TESC | tescalcin | 54997 | 0.9 | 0.0264009 | 0.0285 |
| 234597_at | | | | 0.9 | 0.0281798 | 0.0286 |
| 242112_at | LSM11 | LSM11, U7 small nuclear RNA associated | 134353 | 0.9 | 0.0301464 | 0.0291 |
| 211001_at | TRIM29 | tripartite motif containing 29 | 23650 | 0.9 | 0.0277792 | 0.0292 |
| 229827_at | BUB3 | BUB3 mitotic checkpoint protein | 9184 | 0.9 | 0.0253813 | 0.0293 |
| 237215_s_at | TFRC | transferrin receptor (p90, CD71) | 7037 | 0.9 | 0.0254396 | 0.0303 |
| 231164_at | ABCA17P | ATP-binding cassette, sub-family A (ABC1), member 17, pseudogene | 650655 | 0.9 | 0.0272825 | 0.0306 |
| 240382_at | | | | 0.9 | 0.0323965 | 0.0308 |
| 213497_at | ABTB2 | ankyrin repeat and BTB (POZ) domain containing 2 | 25841 | 0.9 | 0.0256219 | 0.0311 |
| 235090_at | ZNF710 | zinc finger protein 710 | 374655 | 0.9 | 0.0270066 | 0.0312 |
| 1556555_at | LOC1001294 | uncharacterized LOC100129461 | 100129461 | 0.9 | 0.0283036 | 0.0314 |
| 210885_s_at | TRIM15 | tripartite motif containing 15 | 89870 | 0.9 | 0.0257848 | 0.0315 |
| 211695_x_at | MUC1 | mucin 1, cell surface associated | 4582 | 0.9 | 0.0307873 | 0.0317 |
| 223812_at | FAM178B | family with sequence similarity 178, member B | 51252 | 0.9 | 0.0330063 | 0.0324 |
| 1554476_x_a | ZNF808 | zinc finger protein 808 | 388558 | 0.9 | 0.0302282 | 0.0326 |
| 223372_at | | | | 0.9 | 0.0321308 | 0.0326 |
| 1564451_at | | | | 0.9 | 0.0327703 | 0.0326 |
| 213551_x_at | | | | 0.9 | 0.0289508 | 0.0327 |
| 210287_s_at | FLT1 | fms-related tyrosine kinase 1 | 2321 | 0.9 | 0.029942 | 0.034 |
| 236353_at | | | | 0.9 | 0.0332268 | 0.034 |
| 1570414_x_a | FLJ13197 | uncharacterized FLJ13197 | 79667 | 0.9 | 0.0321492 | 0.0347 |
| 230706_s_at | CAMK2N2 | calcium/calmodulin-dependent protein kinase II inhibitor 2 | 94032 | 0.9 | 0.0351052 | 0.0349 |
| 1558586_at | ZNF33B | zinc finger protein 33B | 7582 | 0.9 | 0.0314127 | 0.035 |
| 216854_at | GDF11 | growth differentiation factor 11 | 10220 | 0.9 | 0.0312732 | 0.0351 |
| 204404_at | SLC12A2 | solute carrier family 12 (sodium/potassium/chloride transporters), member 2 | 6558 | 0.9 | 0.0305859 | 0.0352 |
| 1560512_at | | | | 0.9 | 0.0308252 | 0.0356 |
| 240642_at | ZMYM2 | zinc finger, MYM-type 2 | 7750 | 0.9 | 0.0332682 | 0.0362 |
| 217579_x_at | | | | 0.9 | 0.0349844 | 0.0363 |
| 1560617_at | LOC1001281 | uncharacterized LOC100128198 | 100128198 | 0.9 | 0.0319561 | 0.0364 |
| 212551_at | CAP2 | CAP, adenylate cyclase-associated protein, 2 (yeast) | 10486 | 0.9 | 0.0340561 | 0.0366 |
| 1570241_at | SPATA21 | spermatogenesis associated 21 | 374955 | 0.9 | 0.0364431 | 0.0367 |
| 230830_at | SLC51B | solute carrier family 51, beta subunit | 123264 | 0.9 | 0.0342591 | 0.0371 |
| 230134_s_at | RC3H2 | ring finger and CCCH-type domains 2 | 54542 | 0.9 | 0.0351286 | 0.0373 |
| 242148_at | BCL6B | B-cell CLL/lymphoma 6, member B | 255877 | 0.9 | 0.033291 | 0.0378 |
| 223808_s_at | | | | 0.9 | 0.0351113 | 0.0379 |
| 1569629_x_a | LOC389906 | zinc finger protein 839 pseudogene | 389906 | 0.9 | 0.0370934 | 0.0381 |
| 1559745_at | | | | 0.9 | 0.0339276 | 0.0382 |
| 203786_s_at | TPD52L1 | tumor protein D52-like 1 | 7164 | 0.9 | 0.0362088 | 0.0386 |
| 1553799_at | FAM227B | family with sequence similarity 227, member B | 196951 | 0.9 | 0.0369132 | 0.0386 |
| 1552493_s_a | CYP11B1 | cytochrome P450, family 11, subfamily B, polypeptide 1 | 1584 | 0.9 | 0.0326075 | 0.0388 |
| 204440_at | CD83 | CD83 molecule | 9308 | 0.9 | 0.0377237 | 0.0391 |
| 214596_at | CHRM3 | cholinergic receptor, muscarinic 3 | 1131 | 0.9 | 0.0337095 | 0.0396 |
| 1554934_at | RCBTB1 | regulator of chromosome condensation (RCC1) and BTB (POZ) domain containing protein 1 | 55213 | 0.9 | 0.0388819 | 0.0408 |
| 205058_at | | | | 0.9 | 0.0331916 | 0.0411 |
| 234416_at | | | | 0.9 | 0.036782 | 0.0411 |
| 218759_at | DVL2 | dishevelled, dsh homolog 2 (Drosophila) | 1856 | 0.9 | 0.0348284 | 0.0412 |
| 1564039_at | ZSCAN23 | zinc finger and SCAN domain containing 23 | 222696 | 0.9 | 0.0359598 | 0.0419 |
| 219927_at | FCF1 | FCF1 small subunit (SSU) processome component homolog (S. cerevisiae) | 51077 | 0.9 | 0.0357238 | 0.042 |
| 237909_at | ADAM6 | ADAM metallopeptidase domain 6, pseudogene | 8755 | 0.9 | 0.0345966 | 0.0422 |
| 241131_at | | | | 0.9 | 0.040294 | 0.0424 |
| 216001_at | PRAMEF12 | PRAME family member 12 | 390999 | 0.9 | 0.0375392 | 0.0426 |
| 213675_at | PARVA | parvin, alpha | 55742 | 0.9 | 0.0355853 | 0.043 |
| 222577_at | CCDC90B | coiled-coil domain containing 90B | 60492 | 0.9 | 0.035807 | 0.0434 |
| 219442_at | CLUHP3 | clustered mitochondria (cluA/CLU1) homolog pseudogene 3 | 100132341 | 0.9 | 0.0381317 | 0.0436 |
| 231065_at | PDE6D | phosphodiesterase 6D, cGMP-specific, rod, delta | 5147 | 0.9 | 0.0379607 | 0.0437 |

| ProbeSet | Symbol | Name | EntrezID | Fold Change | Parametric p-value | Permutation p-value |
|---|---|---|---|---|---|---|
| 1562743_at | ZNF33B | zinc finger protein 33B | 7582 | 0.9 | 0.0385602 | 0.044 |
| 222568_at | UGGT1 | UDP-glucose glycoprotein glucosyltransferase 1 | 56886 | 0.9 | 0.0377951 | 0.0447 |
| 210082_at | ABCA4 | ATP-binding cassette, sub-family A (ABC1), member 4 | 24 | 0.9 | 0.0406824 | 0.0457 |
| 1557385_at | FAM161A | family with sequence similarity 161, member A | 84140 | 0.9 | 0.0414434 | 0.0463 |
| 1558778_s_a | MKL2 | MKL/myocardin-like 2 | 57496 | 0.9 | 0.0416179 | 0.047 |
| 223323_x_at | TRPM7 | transient receptor potential cation channel, subfamily M, member 7 | 54822 | 0.9 | 0.0424069 | 0.0481 |
| 1553593_a_a | TAL2 | T-cell acute lymphocytic leukemia 2 | 6887 | 0.9 | 0.0427086 | 0.0485 |
| 204664_at | ALPP | alkaline phosphatase, placental | 250 | 0.9 | 0.0427549 | 0.0487 |
| 242446_at | C6orf163 | chromosome 6 open reading frame 163 | 206412 | 0.9 | 0.0432151 | 0.049 |
| 222766_at | POLR3K | polymerase (RNA) III (DNA directed) polypeptide K, 12.3 kDa | 51728 | 0.9 | 0.0463059 | 0.0494 |
| 222267_at | TMEM209 | transmembrane protein 209 | 84928 | 0.9 | 0.0423552 | 0.0496 |
| 209961_s_at | HGF | hepatocyte growth factor (hepapoietin A; scatter factor) | 3082 | 0.89 | 0.0025573 | 5.00E-04 |
| 1569172_a_a | LOC402160 | uncharacterized LOC402160 | 402160 | 0.89 | 0.001958 | 7.00E-04 |
| 240380_at | LOC728040 | hCG1813624 | 728040 | 0.89 | 0.0016657 | 9.00E-04 |
| 230212_at | SPRY1 | sprouty homolog 1, antagonist of FGF signaling (Drosophila) | 10252 | 0.89 | 0.0020482 | 0.0012 |
| 233927_at | | | | 0.89 | 0.0032643 | 0.0022 |
| 243859_at | | | | 0.89 | 0.0047761 | 0.0035 |
| 1564868_a_a | FAM117B | family with sequence similarity 117, member B | 150864 | 0.89 | 0.0054997 | 0.0042 |
| 214212_x_at | FERMT2 | fermitin family member 2 | 10979 | 0.89 | 0.0051674 | 0.0046 |
| 239729_at | | | | 0.89 | 0.0074545 | 0.0053 |
| 1563086_at | | | | 0.89 | 0.0074769 | 0.0065 |
| 210671_x_at | MAPK8 | mitogen-activated protein kinase 8 | 5599 | 0.89 | 0.009603 | 0.0067 |
| 240790_at | | | | 0.89 | 0.007823 | 0.0069 |
| 231537_at | | | | 0.89 | 0.0098654 | 0.007 |
| 1559646_a_a | LINC00184 | long intergenic non-protein coding RNA 184 | 100302691 | 0.89 | 0.0075121 | 0.0074 |
| 1566931_at | TFB2M | transcription factor B2, mitochondrial | 64216 | 0.89 | 0.0076004 | 0.008 |
| 1561459_at | | | | 0.89 | 0.0075827 | 0.0081 |
| 213299_at | ZBTB7A | zinc finger and BTB domain containing 7A | 51341 | 0.89 | 0.0094171 | 0.0083 |
| 217467_at | | | | 0.89 | 0.0090932 | 0.0091 |
| 236392_at | CUL9 | cullin 9 | 23113 | 0.89 | 0.0084791 | 0.0098 |
| 236005_at | | | | 0.89 | 0.010073 | 0.01 |
| 244855_at | | | | 0.89 | 0.0132854 | 0.0115 |
| 225211_at | PVRL1 | poliovirus receptor-related 1 (herpesvirus entry mediator C) | 5818 | 0.89 | 0.0098845 | 0.0116 |
| 236034_at | ANGPT2 | angiopoietin 2 | 285 | 0.89 | 0.0132113 | 0.0123 |
| 215345_x_at | TARP | TCR gamma alternate reading frame protein | 445347 | 0.89 | 0.0104973 | 0.0124 |
| 1557918_s_a | SLC16A1 | solute carrier family 16, member 1 (monocarboxylic acid transporter 1) | 6566 | 0.89 | 0.012761 | 0.0126 |
| 211186_s_at | AAK1 | AP2 associated kinase 1 | 22848 | 0.89 | 0.0130949 | 0.013 |
| 225465_at | MAGI1 | membrane associated guanylate kinase, WW and PDZ domain containing 1 | 9223 | 0.89 | 0.0159393 | 0.013 |
| 214546_s_at | | | | 0.89 | 0.0133072 | 0.0133 |
| 1570327_at | C20orf62 | chromosome 20 open reading frame 62 | 140834 | 0.89 | 0.0120658 | 0.0136 |
| 243590_at | | | | 0.89 | 0.0140454 | 0.0136 |
| 1553470_at | DNAH17 | dynein, axonemal, heavy chain 17 | 8632 | 0.89 | 0.0141366 | 0.014 |
| 241259_at | GAB3 | GRB2-associated binding protein 3 | 139716 | 0.89 | 0.014535 | 0.0144 |
| 1557759_at | ATP5SL | ATP5S-like | 55101 | 0.89 | 0.0154064 | 0.0145 |
| 206870_at | PPARA | peroxisome proliferator-activated receptor alpha | 5465 | 0.89 | 0.013548 | 0.0146 |
| 240219_at | LINC00327 | long intergenic non-protein coding RNA 327 | 100506697 | 0.89 | 0.0130565 | 0.0156 |
| 216690_at | OR7C1 | olfactory receptor, family 7, subfamily C, member 1 | 26664 | 0.89 | 0.0166863 | 0.0161 |
| 235087_at | UNKL | unkempt homolog (Drosophila)-like | 64718 | 0.89 | 0.0153421 | 0.0163 |
| 203129_s_at | KIF5C | kinesin family member 5C | 3800 | 0.89 | 0.0186166 | 0.0179 |
| 1552769_at | ZNF625 | zinc finger protein 625 | 90589 | 0.89 | 0.015283 | 0.0183 |
| 229792_at | KLHL17 | kelch-like family member 17 | 339451 | 0.89 | 0.0196401 | 0.0183 |
| 1561242_at | | | | 0.89 | 0.0196704 | 0.0187 |
| 243147_x_at | | | | 0.89 | 0.0177572 | 0.0191 |
| 1566740_at | PLCE1 | phospholipase C, epsilon 1 | 51196 | 0.89 | 0.0182048 | 0.0193 |
| 1565783_at | TNK2 | tyrosine kinase, non-receptor, 2 | 10188 | 0.89 | 0.0187754 | 0.0193 |
| 233593_at | | | | 0.89 | 0.0166658 | 0.0196 |
| 1557893_a_a | LOC1001445 | uncharacterized LOC100144597 | 100144597 | 0.89 | 0.0180012 | 0.0206 |
| 221624_at | TCL6 | T-cell leukemia/lymphoma 6 (non-protein coding) | 27004 | 0.89 | 0.017333 | 0.0208 |
| 1557124_at | TMEM198B | transmembrane protein 198B, pseudogene | 440104 | 0.89 | 0.0169695 | 0.0215 |
| 214744_s_at | RPL23 | ribosomal protein L23 | 9349 | 0.89 | 0.0183033 | 0.0218 |
| 218766_s_at | WARS2 | tryptophanyl tRNA synthetase 2, mitochondrial | 10352 | 0.89 | 0.0190403 | 0.0222 |
| 1552576_at | ALMS1P | Alstrom syndrome 1 pseudogene | 200420 | 0.89 | 0.0180976 | 0.0223 |
| 230124_at | | | | 0.89 | 0.0206869 | 0.0227 |
| 211737_x_at | PTN | pleiotrophin | 5764 | 0.89 | 0.018113 | 0.0228 |
| 225151_at | RTKN | rhotekin | 6242 | 0.89 | 0.0206332 | 0.023 |
| 217019_at | | | | 0.89 | 0.0199408 | 0.0236 |
| 241725_at | E2F7 | E2F transcription factor 7 | 144455 | 0.89 | 0.0205331 | 0.0242 |
| 217364_x_at | | | | 0.89 | 0.0195033 | 0.0245 |
| 238112_at | LOC283177 | uncharacterized LOC283177 | 283177 | 0.89 | 0.022186 | 0.0257 |
| 208492_at | RFXAP | regulatory factor X-associated protein | 5994 | 0.89 | 0.0238011 | 0.0257 |
| 222796_at | | | | 0.89 | 0.0238067 | 0.0265 |
| 233734_s_at | OSBPL5 | oxysterol binding protein-like 5 | 114879 | 0.89 | 0.0248396 | 0.0268 |
| 206894_at | APOA4 | apolipoprotein A-IV | 337 | 0.89 | 0.0245879 | 0.0278 |
| 220057_at | | | | 0.89 | 0.0267641 | 0.0282 |
| 211189_x_at | CD84 | CD84 molecule | 8832 | 0.89 | 0.0277933 | 0.0297 |
| 211588_s_at | PML | promyelocytic leukemia | 5371 | 0.89 | 0.0257065 | 0.0302 |
| 231196_x_at | LINC00202-1 | long intergenic non-protein coding RNA 202-1 | 387644 | 0.89 | 0.0285126 | 0.0308 |
| 222813_at | ZNF668 | zinc finger protein 668 | 79759 | 0.89 | 0.0229978 | 0.0311 |

| ProbeSet | Symbol | Name | EntrezID | Fold Change | Parametric p-value | Permutation p-value |
|---|---|---|---|---|---|---|
| 230792_at | FAAH2 | fatty acid amide hydrolase 2 | 158584 | 0.89 | 0.0294354 | 0.0327 |
| 1558844_at | LOC1005061 | putative uncharacterized protein FLJ37770-like | 100506127 | 0.89 | 0.0306746 | 0.033 |
| 206831_s_at | ARSD | arylsulfatase D | 414 | 0.89 | 0.0262895 | 0.0331 |
| 222907_x_at | TMEM50B | transmembrane protein 50B | 757 | 0.89 | 0.0285022 | 0.0331 |
| 1554105_at | TMEM185A | transmembrane protein 185A | 84548 | 0.89 | 0.0306873 | 0.0332 |
| 236670_s_at | | | | 0.89 | 0.0268523 | 0.0336 |
| 222234_s_at | DBNDD1 | dysbindin (dystrobrevin binding protein 1) domain containing 1 | 79007 | 0.89 | 0.0272383 | 0.0338 |
| 225983_s_at | VWA1 | von Willebrand factor A domain containing 1 | 64856 | 0.89 | 0.0280788 | 0.0342 |
| 1557744_at | | | | 0.89 | 0.0319578 | 0.0347 |
| 204963_at | SSPN | sarcospan | 8082 | 0.89 | 0.0286029 | 0.0348 |
| 222224_at | NACA2 | nascent polypeptide-associated complex alpha subunit 2 | 342538 | 0.89 | 0.0305701 | 0.035 |
| 226626_at | THOC2 | THO complex 2 | 57187 | 0.89 | 0.0279984 | 0.0352 |
| 240455_at | | | | 0.89 | 0.0309487 | 0.0353 |
| 220433_at | PRRG3 | proline rich Gla (G-carboxyglutamic acid) 3 (transmembrane) | 79057 | 0.89 | 0.0334809 | 0.0364 |
| 223657_at | FAM167B | family with sequence similarity 167, member B | 84734 | 0.89 | 0.0340656 | 0.0411 |
| 230038_at | ATXN7L2 | ataxin 7-like 2 | 127002 | 0.89 | 0.0329286 | 0.0415 |
| 239590_x_at | FAM95B1 | family with sequence similarity 95, member B1 | 100133036 | 0.89 | 0.035043 | 0.0423 |
| 218408_at | TIMM10 | translocase of inner mitochondrial membrane 10 homolog (yeast) | 26519 | 0.89 | 0.0382085 | 0.0428 |
| 1570297_at | | | | 0.89 | 0.0360871 | 0.0435 |
| 220575_at | FAM106A | family with sequence similarity 106, member A | 80039 | 0.89 | 0.035631 | 0.0437 |
| 237795_s_at | SP2 | Sp2 transcription factor | 6668 | 0.89 | 0.0357815 | 0.0437 |
| 204979_s_at | SH3BGR | SH3 domain binding glutamic acid-rich protein | 6450 | 0.89 | 0.0364695 | 0.0439 |
| 228508_at | MAML3 | mastermind-like 3 (Drosophila) | 55534 | 0.89 | 0.036311 | 0.0441 |
| 236949_at | | | | 0.89 | 0.0382467 | 0.0452 |
| 234753_x_at | | | | 0.89 | 0.0381873 | 0.0454 |
| 228843_at | ARL10 | ADP-ribosylation factor-like 10 | 285598 | 0.89 | 0.0368531 | 0.0457 |
| 239523_at | TUSC5 | tumor suppressor candidate 5 | 286753 | 0.89 | 0.0402364 | 0.0458 |
| 1560451_at | | | | 0.89 | 0.0368261 | 0.0459 |
| 228512_at | PTCD3 | pentatricopeptide repeat domain 3 | 55037 | 0.89 | 0.0384212 | 0.0461 |
| 223131_s_at | TRIM8 | tripartite motif containing 8 | 81603 | 0.89 | 0.0397913 | 0.0476 |
| 239163_at | UBE2B | ubiquitin-conjugating enzyme E2B | 7320 | 0.89 | 0.0395911 | 0.0486 |
| 231873_at | BMPR2 | bone morphogenetic protein receptor, type II (serine/threonine kinase) | 659 | 0.89 | 0.043463 | 0.0498 |
| 243797_at | STK17B | serine/threonine kinase 17b | 9262 | 0.88 | 0.0019413 | 7.00E-04 |
| 214616_at | | | | 0.88 | 0.0042799 | 0.0031 |
| 204397_at | EML2 | echinoderm microtubule associated protein like 2 | 24139 | 0.88 | 0.0052069 | 0.0034 |
| 206056_x_at | SPN | sialophorin | 6693 | 0.88 | 0.0047397 | 0.0037 |
| 231311_at | | | | 0.88 | 0.0028016 | 0.004 |
| 216169_at | | | | 0.88 | 0.0037209 | 0.0041 |
| 1555122_at | GPR125 | G protein-coupled receptor 125 | 166647 | 0.88 | 0.008327 | 0.0043 |
| 221793_at | DOCK6 | dedicator of cytokinesis 6 | 57572 | 0.88 | 0.006178 | 0.0063 |
| 241570_at | | | | 0.88 | 0.0063029 | 0.0063 |
| 237905_at | KRT25 | keratin 25 | 147183 | 0.88 | 0.0078108 | 0.0068 |
| 244237_at | FLJ90680 | FLJ90680 protein | 400926 | 0.88 | 0.0068938 | 0.0087 |
| 1562739_at | LOC285593 | uncharacterized LOC285593 | 285593 | 0.88 | 0.0102114 | 0.0091 |
| 208554_at | POU4F3 | POU class 4 homeobox 3 | 5459 | 0.88 | 0.0093463 | 0.0098 |
| 231068_at | SLC47A2 | solute carrier family 47, member 2 | 146802 | 0.88 | 0.0085228 | 0.0109 |
| 213926_s_at | AGFG1 | ArfGAP with FG repeats 1 | 3267 | 0.88 | 0.0091407 | 0.0117 |
| 234425_at | | | | 0.88 | 0.0094265 | 0.0122 |
| 230537_at | | | | 0.88 | 0.0100867 | 0.0133 |
| 234132_at | | | | 0.88 | 0.0128196 | 0.0138 |
| 216018_at | RNF5 | ring finger protein 5, E3 ubiquitin protein ligase | 6048 | 0.88 | 0.0126314 | 0.0139 |
| 240777_at | SYNE2 | spectrin repeat containing, nuclear envelope 2 | 23224 | 0.88 | 0.0129971 | 0.0159 |
| 217073_x_at | APOA1 | apolipoprotein A-I | 335 | 0.88 | 0.0145869 | 0.016 |
| 1553775_at | FLJ31715 | uncharacterized FLJ31715 | 152048 | 0.88 | 0.0120683 | 0.0166 |
| 244322_at | LOC646329 | uncharacterized LOC646329 | 646329 | 0.88 | 0.0146709 | 0.0169 |
| 211386_at | MGC12488 | uncharacterized protein MGC12488 | 84786 | 0.88 | 0.0144013 | 0.0186 |
| 241440_at | | | | 0.88 | 0.0147279 | 0.0188 |
| 1554193_s_a | MANEA | mannosidase, endo-alpha | 79694 | 0.88 | 0.0159258 | 0.0191 |
| 244716_x_at | TMIGD2 | transmembrane and immunoglobulin domain containing 2 | 126259 | 0.88 | 0.0167226 | 0.0206 |
| 233765_at | | | | 0.88 | 0.0213427 | 0.0245 |
| 215510_at | ETV2 | ets variant 2 | 2116 | 0.88 | 0.0192028 | 0.0252 |
| 225914_s_at | CAB39L | calcium binding protein 39-like | 81617 | 0.88 | 0.0210837 | 0.0262 |
| 222372_at | MAGI1 | membrane associated guanylate kinase, WW and PDZ domain containing 1 | 9223 | 0.88 | 0.0266955 | 0.0281 |
| 237877_at | | | | 0.88 | 0.0212034 | 0.0299 |
| 1565436_s_a | MLL | myeloid/lymphoid or mixed-lineage leukemia (trithorax homolog, Drosophila) | 4297 | 0.88 | 0.0240505 | 0.0308 |
| 237380_at | | | | 0.88 | 0.0253402 | 0.0313 |
| 205468_s_at | IRF5 | interferon regulatory factor 5 | 3663 | 0.88 | 0.0243844 | 0.033 |
| 241322_at | LOC1009966 | uncharacterized LOC100996613 | 100996613 | 0.88 | 0.0270811 | 0.0348 |
| 228140_s_at | PPP2R2C | protein phosphatase 2, regulatory subunit B, gamma | 5522 | 0.88 | 0.0294445 | 0.0366 |
| 238975_at | MMAB | methylmalonic aciduria (cobalamin deficiency) cblB type | 326625 | 0.88 | 0.0268184 | 0.0374 |
| 239517_at | ITGB6 | integrin, beta 6 | 3694 | 0.88 | 0.0310204 | 0.0378 |
| 233541_at | LIMD1-AS1 | LIMD1 antisense RNA 1 | 644714 | 0.88 | 0.0281316 | 0.0384 |
| 223929_s_at | | | | 0.88 | 0.030152 | 0.0402 |
| 228116_at | DUXAP10 | double homeobox A pseudogene 10 | 503639 | 0.88 | 0.0332056 | 0.0423 |
| 232976_at | | | | 0.88 | 0.0326897 | 0.0436 |
| 1565802_at | | | | 0.88 | 0.0354818 | 0.0441 |
| 223865_at | SOX6 | SRY (sex determining region Y)-box 6 | 55553 | 0.88 | 0.0336243 | 0.0448 |
| 1554341_a_a | HELQ | helicase, POLQ-like | 113510 | 0.88 | 0.0343904 | 0.0452 |

| ProbeSet | Symbol | Name | EntrezID | Fold Change | Parametric p-value | Permutation p-value |
|---|---|---|---|---|---|---|
| 1568926_x_a | MYLK3 | myosin light chain kinase 3 | 91807 | 0.88 | 0.0356188 | 0.0459 |
| 244296_at | | | | 0.88 | 0.0359794 | 0.0462 |
| 1556134_a_a | B3GNT5 | UDP-GlcNAc:betaGal beta-1,3-N-acetylglucosaminyltransferase 5 | 84002 | 0.87 | 0.0008276 | 3.00E-04 |
| 1560911_at | LOC1001334 | uncharacterized LOC100133461 | 100133461 | 0.87 | 0.0012019 | 0.001 |
| 226288_s_at | NLGN2 | neuroligin 2 | 57555 | 0.87 | 0.0012387 | 0.0012 |
| 216615_s_at | HTR3A | 5-hydroxytryptamine (serotonin) receptor 3A, ionotropic | 3359 | 0.87 | 0.0007973 | 0.0013 |
| 238855_at | AHNAK | AHNAK nucleoprotein | 79026 | 0.87 | 0.0021545 | 0.0021 |
| 1563946_at | | | | 0.87 | 0.0032695 | 0.0032 |
| 230191_at | TTBK1 | tau tubulin kinase 1 | 84630 | 0.87 | 0.0041001 | 0.004 |
| 1560322_at | RBMS3 | RNA binding motif, single stranded interacting protein 3 | 27303 | 0.87 | 0.0035428 | 0.0045 |
| 215801_at | | | | 0.87 | 0.0047465 | 0.0051 |
| 1559148_at | LOC1010606 | uncharacterized LOC101060609 | 101060609 | 0.87 | 0.0036502 | 0.0055 |
| 1558474_at | | | | 0.87 | 0.0039938 | 0.0056 |
| 222091_at | HPCAL4 | hippocalcin like 4 | 51440 | 0.87 | 0.004408 | 0.0063 |
| 236786_at | FAM135B | family with sequence similarity 135, member B | 51059 | 0.87 | 0.0044225 | 0.0063 |
| 1555490_s_a | PDZD3 | PDZ domain containing 3 | 79849 | 0.87 | 0.0051071 | 0.008 |
| 243133_at | TSC22D1 | TSC22 domain family, member 1 | 8848 | 0.87 | 0.0068187 | 0.0086 |
| 205798_at | IL7R | interleukin 7 receptor | 3575 | 0.87 | 0.0083513 | 0.0107 |
| 238844_s_at | NPHP1 | nephronophthisis 1 (juvenile) | 4867 | 0.87 | 0.0088152 | 0.0117 |
| 1553991_s_a | VSIG10 | V-set and immunoglobulin domain containing 10 | 54621 | 0.87 | 0.0117178 | 0.0129 |
| 236598_at | LOC1009965 | uncharacterized LOC100996579 | 100996579 | 0.87 | 0.0108273 | 0.013 |
| 241825_at | TYW5 | tRNA-yW synthesizing protein 5 | 129450 | 0.87 | 0.0105138 | 0.0139 |
| 1563036_at | | | | 0.87 | 0.0120737 | 0.0141 |
| 235628_x_at | FLJ33630 | uncharacterized LOC644873 | 644873 | 0.87 | 0.011204 | 0.0157 |
| 212773_s_at | TOMM20 | translocase of outer mitochondrial membrane 20 homolog (yeast) | 9804 | 0.87 | 0.0113791 | 0.0157 |
| 223779_at | AFAP1-AS1 | AFAP1 antisense RNA 1 | 84740 | 0.87 | 0.0097489 | 0.0162 |
| 236041_at | | | | 0.87 | 0.0119378 | 0.0162 |
| 1560784_x_at | | | | 0.87 | 0.0135971 | 0.0164 |
| 240531_at | | | | 0.87 | 0.0115235 | 0.0167 |
| 219321_at | MPP5 | membrane protein, palmitoylated 5 (MAGUK p55 subfamily member 5) | 64398 | 0.87 | 0.0163929 | 0.018 |
| 1558356_at | UACA | uveal autoantigen with coiled-coil domains and ankyrin repeats | 55075 | 0.87 | 0.0172651 | 0.025 |
| 226497_s_at | FLT1 | fms-related tyrosine kinase 1 | 2321 | 0.87 | 0.0188769 | 0.0255 |
| 230824_at | 10-Mar | membrane-associated ring finger (C3HC4) 10, E3 ubiquitin protein ligase | 162333 | 0.87 | 0.0195361 | 0.026 |
| 1557961_s_a | LOC1001279 | uncharacterized LOC100127983 | 100127983 | 0.87 | 0.0248157 | 0.0266 |
| 234981_x_at | CMBL | carboxymethylenebutenolidase homolog (Pseudomonas) | 134147 | 0.87 | 0.0210969 | 0.027 |
| 230879_at | BAG2 | BCL2-associated athanogene 2 | 9532 | 0.87 | 0.0233272 | 0.0271 |
| 234397_at | | | | 0.87 | 0.0211329 | 0.0274 |
| 1561894_at | | | | 0.87 | 0.0248423 | 0.0324 |
| 234875_at | | | | 0.87 | 0.0250647 | 0.0352 |
| 206963_s_at | | | | 0.87 | 0.0248296 | 0.0366 |
| 1553701_a_a | DUSP18 | dual specificity phosphatase 18 | 150290 | 0.87 | 0.0301359 | 0.0397 |
| 244072_at | | | | 0.87 | 0.030465 | 0.0418 |
| 1566633_at | | | | 0.87 | 0.031063 | 0.0421 |
| 232691_at | SFXN5 | sideroflexin 5 | 94097 | 0.87 | 0.0357424 | 0.0473 |
| 215363_x_at | | | | 0.86 | 0.0008305 | 8.00E-04 |
| 205579_at | HRH1 | histamine receptor H1 | 3269 | 0.86 | 0.0024626 | 0.0024 |
| 216684_s_at | SS18 | synovial sarcoma translocation, chromosome 18 | 6760 | 0.86 | 0.0038576 | 0.0026 |
| 210576_at | CYP4F8 | cytochrome P450, family 4, subfamily F, polypeptide 8 | 11283 | 0.86 | 0.0026086 | 0.0028 |
| 223799_at | MSANTD4 | Myb/SANT-like DNA-binding domain containing 4 with coiled-coils | 84437 | 0.86 | 0.0037053 | 0.0044 |
| 1559142_at | KAT6A | K(lysine) acetyltransferase 6A | 7994 | 0.86 | 0.0040818 | 0.0044 |
| 242543_at | SH2D6 | SH2 domain containing 6 | 284948 | 0.86 | 0.004327 | 0.0066 |
| 217291_at | CEACAM5 | carcinoembryonic antigen-related cell adhesion molecule 5 | 1048 | 0.86 | 0.0046376 | 0.0067 |
| 1557630_s_at | | | | 0.86 | 0.0056232 | 0.0068 |
| 236929_at | LOC441242 | uncharacterized LOC441242 | 441242 | 0.86 | 0.0042616 | 0.007 |
| 1562367_at | C15orf54 | chromosome 15 open reading frame 54 | 400360 | 0.86 | 0.005806 | 0.0092 |
| 224803_s_at | | | | 0.86 | 0.0060514 | 0.0097 |
| 241599_at | LSM11 | LSM11, U7 small nuclear RNA associated | 134353 | 0.86 | 0.0061456 | 0.0099 |
| 235920_at | | | | 0.86 | 0.0206491 | 0.0126 |
| 239318_at | FAM118B | family with sequence similarity 118, member B | 79607 | 0.86 | 0.0081207 | 0.0134 |
| 1568853_at | | | | 0.86 | 0.0107249 | 0.0155 |
| 1565756_a_a | METAP1D | methionyl aminopeptidase type 1D (mitochondrial) | 254042 | 0.86 | 0.01204 | 0.0155 |
| 237616_at | | | | 0.86 | 0.0099287 | 0.0159 |
| 1560599_a_a | CEP89 | centrosomal protein 89kDa | 84902 | 0.86 | 0.0099452 | 0.0167 |
| 230808_at | FNTA | farnesyltransferase, CAAX box, alpha | 2339 | 0.86 | 0.0104815 | 0.018 |
| 227735_s_at | C10orf99 | chromosome 10 open reading frame 99 | 387695 | 0.86 | 0.015659 | 0.0202 |
| 232310_at | LOC1005076 | uncharacterized LOC100507637 | 100507637 | 0.86 | 0.0148433 | 0.0233 |
| 238145_at | ZNF496 | zinc finger protein 496 | 84838 | 0.86 | 0.0152321 | 0.0239 |
| 216806_at | | | | 0.86 | 0.016283 | 0.0242 |
| 1554494_at | MTHFSD | methenyltetrahydrofolate synthetase domain containing | 64779 | 0.86 | 0.0176764 | 0.0253 |
| 1556263_s_a | PWRN1 | Prader-Willi region non-protein coding RNA 1 | 791114 | 0.86 | 0.0188301 | 0.0253 |
| 216383_at | | | | 0.86 | 0.0185038 | 0.0267 |
| 211354_s_at | LEPR | leptin receptor | 3953 | 0.86 | 0.0205359 | 0.0281 |
| 242738_s_at | ZFHX3 | zinc finger homeobox 3 | 463 | 0.86 | 0.0200922 | 0.0287 |
| 212509_s_at | MXRA7 | matrix-remodelling associated 7 | 439921 | 0.86 | 0.0222072 | 0.0322 |
| 241762_at | FBXO32 | F-box protein 32 | 114907 | 0.86 | 0.0259888 | 0.0344 |
| 240636_at | | | | 0.86 | 0.0246596 | 0.0356 |
| 211653_x_at | AKR1C2 | aldo-keto reductase family 1, member C2 | 1646 | 0.86 | 0.0264952 | 0.0376 |
| 214595_at | KCNG1 | potassium voltage-gated channel, subfamily G, member 1 | 3755 | 0.86 | 0.0257716 | 0.0381 |

| ProbeSet | Symbol | Name | EntrezID | Fold Change | Parametric p-value | Permutation p-value |
|---|---|---|---|---|---|---|
| 220459_at | MCM3AP-AS | MCM3AP antisense RNA 1 | 114044 | 0.86 | 0.0257584 | 0.0394 |
| 239417_x_at | C6orf52 | chromosome 6 open reading frame 52 | 347744 | 0.86 | 0.0299901 | 0.0405 |
| 213472_at | HNRNPH1 | heterogeneous nuclear ribonucleoprotein H1 (H) | 3187 | 0.86 | 0.030364 | 0.0432 |
| 227501_at | | | | 0.86 | 0.0332136 | 0.0436 |
| 1563445_x_a | CTSL1P8 | cathepsin L1 pseudogene 8 | 1518 | 0.86 | 0.0313583 | 0.0438 |
| 1553634_a_a | FLJ40852 | uncharacterized LOC285962 | 285962 | 0.86 | 0.0301423 | 0.0441 |
| 234785_at | | | | 0.86 | 0.0357542 | 0.0493 |
| 232109_at | UBXN10 | UBX domain protein 10 | 127733 | 0.85 | 0.001898 | 0.0018 |
| 1552893_at | CAMK2N2 | calcium/calmodulin-dependent protein kinase II inhibitor 2 | 94032 | 0.85 | 0.0020063 | 0.0019 |
| 228579_at | KCNQ3 | potassium voltage-gated channel, KQT-like subfamily, member 3 | 3786 | 0.85 | 0.0017189 | 0.0024 |
| 1555294_a_a | ERC1 | ELKS/RAB6-interacting/CAST family member 1 | 23085 | 0.85 | 0.0031317 | 0.0053 |
| 220527_at | MRPL20 | mitochondrial ribosomal protein L20 | 55052 | 0.85 | 0.0083378 | 0.0108 |
| 232866_at | | | | 0.85 | 0.0078075 | 0.0138 |
| 216575_at | | | | 0.85 | 0.0125405 | 0.0173 |
| 238962_at | ZNF681 | zinc finger protein 681 | 148213 | 0.85 | 0.0118147 | 0.0187 |
| 1554512_a_a | CEP89 | centrosomal protein 89kDa | 84902 | 0.85 | 0.0111386 | 0.0191 |
| 216177_at | | | | 0.85 | 0.0148872 | 0.0228 |
| 238968_at | | | | 0.85 | 0.0199964 | 0.0293 |
| 229193_at | LUC7L3 | LUC7-like 3 (S. cerevisiae) | 51747 | 0.85 | 0.0317995 | 0.0447 |
| 240715_at | TBX5 | T-box 5 | 6910 | 0.85 | 0.0312654 | 0.0473 |
| 1560750_at | LOC151121 | uncharacterized LOC151121 | 151121 | 0.85 | 0.0336665 | 0.0497 |
| 1555441_at | UBA6 | ubiquitin-like modifier activating enzyme 6 | 55236 | 0.84 | 0.0005098 | 0.001 |
| 240872_at | | | | 0.84 | 0.0005681 | 0.0015 |
| 206557_at | ZNF702P | zinc finger protein 702, pseudogene | 79986 | 0.84 | 0.0051735 | 0.0017 |
| 229881_at | KLF12 | Kruppel-like factor 12 | 11278 | 0.84 | 0.0050992 | 0.0077 |
| 1564121_at | | | | 0.84 | 0.0052632 | 0.0077 |
| 232975_at | HCG18 | HLA complex group 18 (non-protein coding) | 414777 | 0.84 | 0.0049765 | 0.0085 |
| 243376_at | TANK | TRAF family member-associated NFKB activator | 10010 | 0.84 | 0.0051785 | 0.009 |
| 240796_at | | | | 0.84 | 0.0071637 | 0.0111 |
| 205114_s_at | | | | 0.84 | 0.0195233 | 0.0214 |
| 1569551_at | | | | 0.84 | 0.0148573 | 0.022 |
| 242647_at | USP34 | ubiquitin specific peptidase 34 | 9736 | 0.84 | 0.0146223 | 0.0248 |
| 210349_at | CAMK4 | calcium/calmodulin-dependent protein kinase IV | 814 | 0.84 | 0.0179758 | 0.0256 |
| 1555978_s_a | MYL12A | myosin, light chain 12A, regulatory, non-sarcomeric | 10627 | 0.84 | 0.0222171 | 0.0288 |
| 200834_s_at | RPS21 | ribosomal protein S21 | 6227 | 0.84 | 0.0218585 | 0.0331 |
| 1555363_s_a | LINC00663 | long intergenic non-protein coding RNA 663 | 284440 | 0.84 | 0.0322065 | 0.0479 |
| 1553215_s_a | CCDC7 | coiled-coil domain containing 7 | 221016 | 0.84 | 0.0001304 | < 1e-07 |
| 215352_at | | | | 0.83 | 0.0045107 | 0.0075 |
| 206220_s_at | RASA3 | RAS p21 protein activator 3 | 22821 | 0.83 | 0.0110871 | 0.0209 |
| 223623_at | C2orf40 | chromosome 2 open reading frame 40 | 84417 | 0.83 | 0.020268 | 0.0297 |
| 241394_at | | | | 0.83 | 0.0250253 | 0.0372 |
| 214590_s_at | UBE2D1 | ubiquitin-conjugating enzyme E2D 1 | 7321 | 0.83 | 0.0297814 | 0.045 |
| 225812_at | FAM229B | family with sequence similarity 229, member B | 619208 | 0.82 | 0.0002212 | 2.00E-04 |
| 227326_at | MXRA7 | matrix-remodelling associated 7 | 439921 | 0.82 | 0.0013281 | 0.0014 |
| 1557036_at | ZBTB1 | zinc finger and BTB domain containing 1 | 22890 | 0.82 | 0.0038762 | 0.0055 |
| 1570623_at | | | | 0.82 | 0.0041173 | 0.0066 |
| 236188_s_at | NAP1L4 | nucleosome assembly protein 1-like 4 | 4676 | 0.82 | 0.0048535 | 0.0076 |
| 232800_at | LOC1009969 | uncharacterized LOC100996920 | 100996920 | 0.82 | 0.0142213 | 0.0219 |
| 243683_at | MORF4L2 | mortality factor 4 like 2 | 9643 | 0.82 | 0.0175552 | 0.0262 |
| 1559170_at | ANKRD20A5 | ankyrin repeat domain 20 family, member A5, pseudogene | 440482 | 0.81 | 0.0148151 | 0.0181 |
| 1555926_a_at | | | | 0.81 | 0.0188846 | 0.029 |
| 217602_at | PPIA | peptidylprolyl isomerase A (cyclophilin A) | 5478 | 0.8 | 0.0014436 | 0.0035 |
| 242751_at | | | | 0.8 | 0.0040271 | 0.0083 |
| 1559514_at | LOC1001320 | uncharacterized LOC100132077 | 100132077 | 0.8 | 0.0060378 | 0.0104 |
| 1557275_a_a | TLCD2 | TLC domain containing 2 | 727910 | 0.8 | 0.0095766 | 0.0152 |
| 233600_at | EFCAB6 | EF-hand calcium binding domain 6 | 64800 | 0.79 | 0.0014913 | 0.0023 |
| 1563321_s_a | MLLT10 | myeloid/lymphoid or mixed-lineage leukemia (trithorax homolog, Drosophila); translocated to, 10 | 8028 | 0.79 | 0.0044628 | 0.0091 |
| 1563080_at | | | | 0.79 | 0.0090402 | 0.0164 |
| 243337_at | FREM3 | FRAS1 related extracellular matrix 3 | 166752 | 0.77 | 0.0004172 | 3.00E-04 |
| 243902_at | | | | 0.75 | 0.0007027 | 0.0024 |
| 1561856_at | | | | 0.71 | 0.0180526 | 0.0293 |
| 233621_s_at | ARHGEF12 | Rho guanine nucleotide exchange factor (GEF) 12 | 23365 | 0.66 | 0.0231311 | 0.0396 |

**Table S2:** BP responder-specific gene expression changes based on stringent differential expression criteria (p value <0.05 and FC>1.2).

| **Table S2: BP responder-specific gene expression changes based on stringent differential expression analysis** | | | | | | | | | | |
|---|---|---|---|---|---|---|---|---|---|---|
| probe set | gene | Accession | EntrezGene | Description | Responders_Baseline | Responders_8 weeks | fold change | non-Responders_Baseline | non-Responders_8 weeks | fold change |
| 200928_s_at | RAB14 | g7328165 | Hs.371563 | RAB14, member RAS oncogene family | 21.67 | 26.46 | 1.22 | 23.05 | 22.75 | -1.01 |
| 201549_x_at | KDM5B | g5729977 | Hs.443650 | lysine (K)-specific demethylase 5B | 50.99 | 64 | 1.26 | 51.84 | 57.9 | 1.12 |
| 201617_x_at | CALD1 | g11091984 | Hs.490203 | caldesmon 1 | 17.56 | 14.45 | -1.21 | 16.31 | 16.29 | -1 |
| 202254_at | SIPA1L1 | Hs.172180.0 | Hs.654657 | signal-induced proliferation-associated 1 like 1 | 62.1 | 78.31 | 1.26 | 62.01 | 65.99 | 1.06 |
| 202369_s_at | TRAM2 | g6912449 | Hs.520182 | translocation associated membrane protein 2 | 53.28 | 64.91 | 1.22 | 56.91 | 52.67 | -1.08 |
| 202848_s_at | GRK6 | Hs.76297.0 | Hs.235116 | G protein-coupled receptor kinase 6 | 122.1 | 100.89 | -1.21 | 105.98 | 111.15 | 1.05 |
| 203170_at | RRP8 | Hs.5158.0 | Hs.652255 | ribosomal RNA processing 8, methyltransferase, homolog (yeast) | 21.22 | 25.94 | 1.22 | 21.25 | 23.46 | 1.1 |
| 203527_s_at | APC | g4557318 | Hs.158932 | adenomatous polyposis coli | 12.26 | 15.73 | 1.28 | 14.96 | 13.07 | -1.14 |
| 203821_at | HBEGF | g4503412 | Hs.799 | heparin-binding EGF-like growth factor | 23.32 | 19.08 | -1.22 | 20.59 | 18.79 | -1.1 |
| 204241_at | ACOX3 | Hs.12773.0 | Hs.479122 | acyl-CoA oxidase 3, pristanoyl | 55.19 | 66.28 | 1.2 | 66.78 | 67.22 | 1.01 |
| 204491_at | PDE4D | Hs.172081.0 | Hs.117545 | phosphodiesterase 4D, cAMP-specific | 36.83 | 26.97 | -1.37 | 36.36 | 34.72 | -1.05 |
| 204501_at | NOV | g4505422 | Hs.235935 | nephroblastoma overexpressed | 46.22 | 64.66 | 1.4 | 47.2 | 48.09 | 1.02 |
| 204794_at | DUSP2 | g12707563 | Hs.1183 | dual specificity phosphatase 2 | 50.32 | 41.18 | -1.22 | 48.34 | 45.96 | -1.05 |
| 204933_s_at | TNFRSF11B | g4507566 | Hs.81791 | tumor necrosis factor receptor superfamily, member 11b | 10.17 | 8 | -1.27 | 9.4 | 9.87 | 1.05 |
| 204956_at | MTAP | g6006025 | Hs.193268 | methylthioadenosine phosphorylase | 10.43 | 13.33 | 1.28 | 11.31 | 11.82 | 1.05 |
| 205069_s_at | ARHGAP26 | g7662207 | Hs.654668 | Rho GTPase activating protein 26 | 11.66 | 14.67 | 1.26 | 12.8 | 13.06 | 1.02 |
| 205095_s_at | ATP6V0A1 | g4885084 | Hs.463074 | ATPase, H+ transporting, lysosomal V0 subunit a1 | 22.55 | 28.55 | 1.27 | 22.02 | 20.96 | -1.05 |
| 205114_s_at | CCL3 /// CCL | g4506842 | Hs.514107 | chemokine (C-C motif) ligand 3 /// chemokine (C-C motif) ligand 3-like 1 /// che | 76.43 | 60.42 | -1.27 | 59.83 | 52.33 | -1.14 |
| 205153_s_at | CD40 | g4507580 | Hs.472860 | CD40 molecule, TNF receptor superfamily member 5 | 97.36 | 77.33 | -1.26 | 87.74 | 91.86 | 1.05 |
| 205420_at | PEX7 | g4505730 | Hs.280932 | peroxisomal biogenesis factor 7 | 11.03 | 13.61 | 1.23 | 12.99 | 13.09 | 1.01 |
| 205426_s_at | HIP1 | g2072422 | Hs.329266 | huntingtin interacting protein 1 | 21.37 | 26.77 | 1.25 | 23.69 | 19.94 | -1.19 |
| 205548_s_at | BTG3 | g5802989 | Hs.473420 | BTG family, member 3 | 8.55 | 10.7 | 1.25 | 11.45 | 9.85 | -1.16 |
| 205944_s_at | CLTCL1 | g9257201 | Hs.368266 | clathrin, heavy chain-like 1 | 70.32 | 88.34 | 1.26 | 80.9 | 77.8 | -1.04 |
| 206557_at | ZNF702P | g13430873 | Hs.714428 | zinc finger protein 702, pseudogene | 19.61 | 15.81 | -1.24 | 17.96 | 15.42 | -1.16 |
| 206588_at | DAZL | g4503258 | Hs.131179 | deleted in azoospermia-like | 4.48 | 5.41 | 1.21 | 4.8 | 5.05 | 1.05 |
| 206826_at | PMP2 | g4505908 | Hs.571512 | peripheral myelin protein 2 | 5.97 | 4.73 | -1.26 | 5.13 | 5.55 | 1.08 |
| 207283_at | RPL23AP32 | g9910199 | --- | ribosomal protein L23a pseudogene 32 | 71.63 | 59.48 | -1.2 | 67.24 | 64.73 | -1.04 |
| 207289_at | MMP25 | g4758727 | Hs.654979 | matrix metallopeptidase 25 | 72.81 | 89.05 | 1.22 | 87.74 | 73.34 | -1.2 |
| 208057_s_at | GLI2 | g439899 | Hs.111867 | GLI family zinc finger 2 | 15.61 | 12.74 | -1.23 | 13.84 | 14.35 | 1.04 |
| 208070_s_at | REV3L | g4506482 | Hs.232021 | REV3-like, polymerase (DNA directed), zeta, catalytic subunit | 20.69 | 25.06 | 1.21 | 25.04 | 23.12 | -1.08 |
| 208474_at | CLDN6 | g11141862 | Hs.533779 | claudin 6 | 13.54 | 16.83 | 1.24 | 13.73 | 15.67 | 1.14 |
| 208554_at | POU4F3 | g4505964 | Hs.553499 | POU class 4 homeobox 3 | 21.64 | 18.04 | -1.2 | 20.2 | 19.12 | -1.06 |
| 208599_at | HUWE1 | g6692990 | Hs.136905 | HECT, UBA and WWE domain containing 1, E3 ubiquitin protein ligase | 11.01 | 13.3 | 1.21 | 11.56 | 11 | -1.05 |
| 209791_at | PADI2 | Hs.33455.0 | Hs.33455 | peptidyl arginine deiminase, type II | 1218.11 | 1469.06 | 1.21 | 1248.42 | 1290.92 | 1.03 |
| 209943_at | FBXL4 | g6103636 | Hs.536850 | F-box and leucine-rich repeat protein 4 | 37.99 | 47.14 | 1.24 | 44.55 | 46.28 | 1.04 |
| 210082_at | ABCA4 | g1888526 | Hs.416707 | ATP-binding cassette, sub-family A (ABC1), member 4 | 8.54 | 7.09 | -1.2 | 7.47 | 7.4 | -1.01 |
| 210281_s_at | ZMYM2 | g12052767 | Hs.507433 | zinc finger, MYM-type 2 | 17.21 | 21.95 | 1.28 | 18.29 | 18.45 | 1.01 |
| 210349_at | CAMK4 | g407005 | Hs.591269 | calcium/calmodulin-dependent protein kinase IV | 33.56 | 27.25 | -1.23 | 36.76 | 32.13 | -1.14 |
| 210598_at | --- | g11493408 | --- | --- | 37.31 | 45.33 | 1.21 | 36.96 | 36.22 | -1.02 |
| 210730_s_at | NPY2R | g1063633 | Hs.37125 | neuropeptide Y receptor Y2 | 15.91 | 13.23 | -1.2 | 14.61 | 15.88 | 1.09 |
| 210896_s_at | ASPH | g11991236 | Hs.332422 | aspartate beta-hydroxylase | 14.63 | 19.85 | 1.36 | 17.1 | 14.83 | -1.15 |
| 211242_x_at | KIR2DL4 | g12006296 | Hs.512572 | killer cell immunoglobulin-like receptor, two domains, long cytoplasmic tail, 4 | 17.93 | 14.05 | -1.28 | 14.93 | 14.4 | -1.04 |
| 211354_s_at | LEPR | g1279902 | Hs.723178 | leptin receptor | 19.02 | 14.57 | -1.31 | 16.18 | 15.02 | -1.08 |
| 211386_at | MGC12488 | g13529217 | Hs.659351 | uncharacterized protein MGC12488 | 96.96 | 80.26 | -1.21 | 89.22 | 84.75 | -1.05 |
| 211588_s_at | PML | g12275890 | Hs.526464 | promyelocytic leukemia | 13.04 | 10.62 | -1.23 | 11.28 | 11.3 | 1 |
| 211653_x_at | AKR1C2 /// L | g187444 | Hs.567256 | aldo-keto reductase family 1, member C2 (dihydrodiol dehydrogenase 2; bile ac | 27.37 | 21.36 | -1.28 | 26.54 | 23.34 | -1.14 |
| 212208_at | MED13L | Hs.4084.0 | Hs.603766 | mediator complex subunit 13-like | 70.31 | 86.3 | 1.23 | 57.63 | 62.95 | 1.09 |
| 212241_at | GCOM1 /// N | Hs.6283.0 | Hs.437256 | GRINL1A complex locus 1 /// myocardial zonula adherens protein /// polymeras | 50.77 | 39.96 | -1.27 | 49.32 | 51.76 | 1.05 |
| 212436_at | TRIM33 | Hs.26837.0 | Hs.26837 | tripartite motif containing 33 | 43.26 | 52.78 | 1.22 | 47.31 | 44.89 | -1.05 |
| 212509_s_at | MXRA7 | Hs.250723.2 | Hs.601108 | matrix-remodelling associated 7 | 210.65 | 165.07 | -1.28 | 192.37 | 190.32 | -1.01 |
| 212618_at | ZNF609 | Hs.155979.0 | Hs.595451 | zinc finger protein 609 | 34.63 | 42.87 | 1.24 | 32.64 | 28.93 | -1.13 |

| | | | | | | | | | | |
|---|---|---|---|---|---|---|---|---|---|---|
| 212719_at | PHLPP1 | Hs.38176.0 | Hs.465337 | PH domain and leucine rich repeat protein phosphatase 1 | 294.24 | 363.86 | 1.24 | 307.73 | 344.07 | 1.12 |
| 212963_at | TM2D1 | Hs.180532.3 | Hs.656790 | TM2 domain containing 1 | 49.11 | 59.34 | 1.21 | 49.84 | 49.17 | -1.01 |
| 213380_x_at | MST1P9 | Hs.278657.1 | Hs.655432 | macrophage stimulating 1 (hepatocyte growth factor-like) pseudogene 9 | 46.73 | 38.32 | -1.22 | 47.18 | 45.54 | -1.04 |
| 213413_at | STON1 | Hs.23437.0 | Hs.44385 | stonin 1 | 7.71 | 6.39 | -1.21 | 6.95 | 6.8 | -1.02 |
| 213478_at | KAZN | Hs.27742.0 | Hs.368823 | kazrin, periplakin interacting protein | 60.28 | 80.43 | 1.33 | 65.83 | 64.28 | -1.02 |
| 213587_s_at | ATP6V0E2 | Hs.91379.1 | Hs.698060 | ATPase, H+ transporting V0 subunit e2 | 589.14 | 478.66 | -1.23 | 541.38 | 574.99 | 1.06 |
| 213812_s_at | CAMKK2 | Hs.108708.2 | Hs.297343 | calcium/calmodulin-dependent protein kinase kinase 2, beta | 217.26 | 266.99 | 1.23 | 234.65 | 233.23 | -1.01 |
| 213832_at | KCND3 | Hs.23729.0 | Hs.666367 | potassium voltage-gated channel, Shal-related subfamily, member 3 | 13.12 | 16.66 | 1.27 | 17.28 | 16.65 | -1.04 |
| 214125_s_at | NENF | Hs.109494.3 | Hs.461787 | Neudesin neurotrophic factor | 128.5 | 104.08 | -1.23 | 126.84 | 128.06 | 1.01 |
| 214322_at | CAMK2G | Hs.250857.2 | Hs.523045 | calcium/calmodulin-dependent protein kinase II gamma | 34.86 | 43.69 | 1.25 | 41.54 | 35.19 | -1.18 |
| 214472_at | HIST1H2AD / | Hs.143042.0 | Hs.546315 | histone cluster 1, H2ad /// histone cluster 1, H3a /// histone cluster 1, H3b /// h | 18.91 | 25.69 | 1.36 | 21.15 | 22.48 | 1.06 |
| 214481_at | HIST1H2AM | Hs.134999.0 | Hs.134999 | histone cluster 1, H2am | 25.71 | 21.18 | -1.21 | 23.24 | 23.41 | 1.01 |
| 214595_at | KCNG1 | Hs.118695.0 | Hs.118695 | potassium voltage-gated channel, subfamily G, member 1 | 30.07 | 21.49 | -1.4 | 29.34 | 29.74 | 1.01 |
| 214657_s_at | LOC1006530 | Hs.322149.0 | Hs.523789 | uncharacterized LOC100653017 /// microRNA 612 /// nuclear paraspeckle asser | 32.93 | 40.7 | 1.24 | 34.62 | 32.66 | -1.06 |
| 214674_at | USP19 | Hs.301373.1 | Hs.255596 | ubiquitin specific peptidase 19 | 29.23 | 35.64 | 1.22 | 30.97 | 35.13 | 1.13 |
| 214871_x_at | LOC1002875 | Hs.233354.0 | Hs.463010 | uncharacterized LOC100287590 | 41.94 | 34.51 | -1.22 | 40.43 | 43.95 | 1.09 |
| 215177_s_at | ITGA6 | Hs.227730.2 | Hs.133397 | integrin, alpha 6 | 22.16 | 17.98 | -1.23 | 18.65 | 20.52 | 1.1 |
| 215191_at | --- | Hs.278648.0 | Hs.636888 | --- | 82.23 | 99.64 | 1.21 | 90.7 | 85.02 | -1.07 |
| 215318_at | MINOS1P1 | Hs.184938.0 | --- | mitochondrial inner membrane organizing system 1 pseudogene 1 | 94.96 | 116.11 | 1.22 | 96.17 | 99.38 | 1.03 |
| 215330_at | --- | Hs.226103.0 | Hs.671957 | --- | 9.62 | 11.75 | 1.22 | 11.09 | 11.7 | 1.06 |
| 215352_at | GIMAP1-GIM | Hs.272534.0 | Hs.647079 | GIMAP1-GIMAP5 readthrough /// GTPase, IMAP family member 5 | 37.41 | 29.89 | -1.25 | 35.97 | 32.5 | -1.11 |
| 215801_at | --- | Hs.274568.0 | Hs.610977 | --- | 12.54 | 10.4 | -1.21 | 12.53 | 11.54 | -1.09 |
| 216014_s_at | ZXDA /// ZXD | Hs.159249.0 | Hs.550094 | zinc finger, X-linked, duplicated A /// zinc finger, X-linked, duplicated B | 23.65 | 18.41 | -1.28 | 20.93 | 23.11 | 1.1 |
| 216018_at | RNF5 | Hs.226019.0 | Hs.731774 | ring finger protein 5, E3 ubiquitin protein ligase | 10.6 | 8.5 | -1.25 | 9.41 | 8.95 | -1.05 |
| 216040_x_at | --- | Hs.296751.0 | Hs.677262 | --- | 12.11 | 9.95 | -1.22 | 10.72 | 11.09 | 1.03 |
| 216383_at | --- | Hs.283952.0 | --- | --- | 79.49 | 65.83 | -1.21 | 77.82 | 70.51 | -1.1 |
| 216547_at | --- | Hs.302094.0 | --- | --- | 22.63 | 18.6 | -1.22 | 23.63 | 21.91 | -1.08 |
| 216589_at | --- | Hs.247773.0 | --- | --- | 30.47 | 24.58 | -1.24 | 25.7 | 26.21 | 1.02 |
| 216813_at | --- | Hs.307068.0 | --- | --- | 13.21 | 15.88 | 1.2 | 14.3 | 14.96 | 1.05 |
| 216984_x_at | CKAP2 /// IG | Hs.121508.0 | Hs.449585 // | Cytoskeleton associated protein 2 /// Immunoglobulin lambda constant 1 (Mcg | 392.62 | 286.25 | -1.37 | 269.11 | 320.45 | 1.19 |
| 217044_s_at | PLEKHG3 | Hs.198037.2 | Hs.509637 | pleckstrin homology domain containing, family G (with RhoGef domain) membe | 24.98 | 30.75 | 1.23 | 22.19 | 24.53 | 1.11 |
| 217266_at | RPL15 | Hs.247851.0 | Hs.381219 | ribosomal protein L15 | 207.55 | 164.83 | -1.26 | 204.49 | 185.89 | -1.1 |
| 217291_at | CEACAM5 | Hs.166040.0 | Hs.709196 | carcinoembryonic antigen-related cell adhesion molecule 5 | 10.65 | 8.66 | -1.23 | 10.03 | 9.25 | -1.08 |
| 217295_at | MUC8 | Hs.1607.0 | Hs.592357 | mucin 8 | 13.66 | 18.68 | 1.37 | 15.94 | 14.46 | -1.1 |
| 217405_x_at | GPLD1 | Hs.272529.1 | Hs.533291 | glycosylphosphatidylinositol specific phospholipase D1 | 10.83 | 13.05 | 1.2 | 12.04 | 10.4 | -1.16 |
| 217507_at | SLC11A1 | Hs.265982.0 | Hs.591607 | solute carrier family 11 (proton-coupled divalent metal ion transporters), meml | 44.31 | 56.63 | 1.28 | 55.19 | 53.75 | -1.03 |
| 217534_at | FAM49B | Hs.282721.0 | Hs.126941 | family with sequence similarity 49, member B | 16.7 | 21.4 | 1.28 | 19.81 | 19 | -1.04 |
| 217540_at | NXPE3 | Hs.293253.0 | Hs.595933 | neurexophilin and PC-esterase domain family, member 3 | 12.67 | 17.14 | 1.35 | 14.49 | 15.09 | 1.04 |
| 217622_at | RHBDD3 | Hs.313951.0 | Hs.106730 | rhomboid domain containing 3 | 24.14 | 19.27 | -1.25 | 22.63 | 22.89 | 1.01 |
| 217659_at | --- | Hs.280645.0 | --- | --- | 27.69 | 34.72 | 1.25 | 32.07 | 29.21 | -1.1 |
| 217753_s_at | RPS26 | g4506708 | Hs.447562 | ribosomal protein S26 | 1354.75 | 1059.69 | -1.28 | 1365.95 | 1377.02 | 1.01 |
| 218066_at | SLC12A7 | g5730042 | Hs.172613 | solute carrier family 12 (potassium/chloride transporters), member 7 | 379.9 | 289.08 | -1.31 | 334.28 | 323.71 | -1.03 |
| 218086_at | NPDC1 | g10181099 | Hs.719906 | neural proliferation, differentiation and control, 1 | 93.44 | 76.44 | -1.22 | 87.8 | 89.91 | 1.02 |
| 218218_at | APPL2 | g8922576 | Hs.506603 | adaptor protein, phosphotyrosine interaction, PH domain and leucine zipper co | 47.29 | 58.17 | 1.23 | 47.99 | 47.13 | -1.02 |
| 218406_x_at | NENF | Hs.109494.0 | Hs.461787 | neudesin neurotrophic factor | 66.67 | 55.16 | -1.21 | 68.83 | 73.84 | 1.07 |
| 218407_x_at | NENF | g7019544 | Hs.461787 | neudesin neurotrophic factor | 221.95 | 181.01 | -1.23 | 227.69 | 234.72 | 1.03 |
| 218658_s_at | ACTR8 | g12597636 | Hs.412186 | ARP8 actin-related protein 8 homolog (yeast) | 29.86 | 36.06 | 1.21 | 31.63 | 33.2 | 1.05 |
| 219236_at | PAQR6 | g13376350 | Hs.235873 | progestin and adipoQ receptor family member VI | 70.49 | 85.03 | 1.21 | 83.35 | 82.56 | -1.01 |
| 219320_at | MYO19 | g13376680 | Hs.302051 | myosin XIX | 16.57 | 13.8 | -1.2 | 16.72 | 17.07 | 1.02 |
| 219366_at | AVEN | g9966840 | Hs.555966 | apoptosis, caspase activation inhibitor | 49.85 | 41.48 | -1.2 | 46.31 | 48.63 | 1.05 |
| 219497_s_at | BCL11A | g13375629 | Hs.370549 | B-cell CLL/lymphoma 11A (zinc finger protein) | 137.13 | 169.87 | 1.24 | 162.96 | 169.45 | 1.04 |
| 219928_s_at | CABYR | g6912377 | Hs.511983 | calcium binding tyrosine-(Y)-phosphorylation regulated | 8.53 | 10.28 | 1.2 | 8.31 | 9.28 | 1.12 |
| 220112_at | ANKRD55 | g13375927 | Hs.436214 | ankyrin repeat domain 55 | 100.3 | 83.49 | -1.2 | 91.39 | 89.17 | -1.02 |

| | | | | | | | | | | |
|---|---|---|---|---|---|---|---|---|---|---|
| 220241_at | TMCO3 | g8923574 | Hs.317593 | transmembrane and coiled-coil domains 3 | 6.84 | 8.25 | 1.21 | 6.87 | 6.66 | -1.03 |
| 220662_s_at | HEYL | g7657153 | Hs.472566 | hairy/enhancer-of-split related with YRPW motif-like | 40.78 | 33.89 | -1.2 | 37.11 | 37.75 | 1.02 |
| 220712_at | C8orf60 | g13376485 | --- | chromosome 8 open reading frame 60 | 49.19 | 60.76 | 1.24 | 52.29 | 56.22 | 1.08 |
| 220992_s_at | TRMT1L | g13569898 | Hs.107149 | tRNA methyltransferase 1 homolog (S. cerevisiae)-like | 76.82 | 92.45 | 1.2 | 95.85 | 109.62 | 1.14 |
| 221138_s_at | --- | g7662605 | --- | --- | 16.87 | 20.27 | 1.2 | 19.59 | 18.97 | -1.03 |
| 221669_s_at | ACAD8 | g12805020 | Hs.14791 | acyl-CoA dehydrogenase family, member 8 | 17.73 | 21.78 | 1.23 | 16.76 | 18.59 | 1.11 |
| 221752_at | SSH1 | Hs.60377.0 | Hs.199763 | slingshot homolog 1 (Drosophila) | 35.99 | 44.35 | 1.23 | 34.8 | 32.81 | -1.06 |
| 221805_at | NEFL | Hs.211584.0 | Hs.521461 | neurofilament, light polypeptide | 11.42 | 9.03 | -1.27 | 12.55 | 12.37 | -1.01 |
| 221847_at | LOC1001293 | Hs.45033.1 | --- | chromosome X open reading frame 69 pseudogene | 280.75 | 232.12 | -1.21 | 297.66 | 313.91 | 1.05 |
| 222091_at | HPCAL4 | Hs.272891.1 | Hs.740383 | hippocalcin like 4 | 59.14 | 46.55 | -1.27 | 54.12 | 54.21 | 1 |
| 222113_s_at | EPS15L1 | Hs.147176.1 | Hs.740367 | epidermal growth factor receptor pathway substrate 15-like 1 | 165.81 | 207.29 | 1.25 | 194.57 | 187.46 | -1.04 |
| 222159_at | --- | Hs.287546.0 | Hs.675607 | --- | 19.25 | 23.34 | 1.21 | 20.52 | 23.11 | 1.13 |
| 222214_at | --- | Hs.303001.0 | Hs.648756 | --- | 59.64 | 75.25 | 1.26 | 60.81 | 58.92 | -1.03 |
| 222234_s_at | DBNDD1 | Hs.301394.1 | Hs.301394 | dysbindin (dystrobrevin binding protein 1) domain containing 1 | 37.94 | 31.56 | -1.2 | 34.95 | 33.43 | -1.05 |
| 39313_at | WNK1 | 4856616_RC | Hs.731621 | WNK lysine deficient protein kinase 1 | 21.43 | 25.89 | 1.21 | 22.05 | 23.34 | 1.06 |
| 222755_s_at | CHD7 | Hs.105461.0 | Hs.609549 | chromodomain helicase DNA binding protein 7 | 14.88 | 18.69 | 1.26 | 14.87 | 13.41 | -1.11 |
| 222813_at | ZNF668 | Hs.102928.0 | Hs.102928 | zinc finger protein 668 | 9.51 | 7.72 | -1.23 | 8.68 | 8.53 | -1.02 |
| 223131_s_at | TRIM8 | Hs.54580.0 | Hs.336810 | tripartite motif containing 8 | 9.67 | 7.92 | -1.22 | 8.8 | 8.64 | -1.02 |
| 223194_s_at | SLC22A23 | g12224886 | Hs.713588 | solute carrier family 22, member 23 | 32.34 | 26.89 | -1.2 | 35.21 | 38.96 | 1.11 |
| 223393_s_at | TSHZ3 | g12053120 | Hs.278436 | teashirt zinc finger homeobox 3 | 90.22 | 114.87 | 1.27 | 96.46 | 99.09 | 1.03 |
| 223623_at | C2orf40 | g11991655 | Hs.43125 | chromosome 2 open reading frame 40 | 22.25 | 18.47 | -1.21 | 27.45 | 24.43 | -1.12 |
| 223747_x_at | WWOX | g8927394 | Hs.461453 | WW domain containing oxidoreductase | 7.34 | 8.98 | 1.22 | 7.48 | 8.02 | 1.07 |
| 223779_at | AFAP1-AS1 | g13325159 | Hs.663029 | AFAP1 antisense RNA 1 (non-protein coding) | 18.4 | 15.09 | -1.22 | 17.62 | 16.59 | -1.06 |
| 223975_at | TRIM51 /// T | g13477118 | Hs.740241 | tripartite motif-containing 51 /// tripartite motif-containing 51G, pseudogene | 16.48 | 12.3 | -1.34 | 12.5 | 14.21 | 1.14 |
| 223983_s_at | C19orf12 | g13436340 | Hs.529094 | chromosome 19 open reading frame 12 | 243.32 | 202.33 | -1.2 | 241.17 | 245.06 | 1.02 |
| 224009_x_at | DHRS9 | g9082136 | Hs.179608 | dehydrogenase/reductase (SDR family) member 9 | 180.46 | 223.99 | 1.24 | 181.06 | 183.53 | 1.01 |
| 224043_s_at | UPB1 | g6288770 | Hs.731656 | ureidopropionase, beta | 12.01 | 14.95 | 1.25 | 12.77 | 13.95 | 1.09 |
| 224225_s_at | ETV7 | g7274371 | Hs.272398 | ets variant 7 | 102.45 | 135.34 | 1.32 | 73.95 | 85.11 | 1.15 |
| 224760_at | SP1 | Hs.9460.0 | Hs.649191 | Sp1 transcription factor | 104.99 | 126.02 | 1.2 | 88.4 | 102.69 | 1.16 |
| 224773_at | NAV1 | Hs.6298.0 | Hs.585374 | neuron navigator 1 | 13.63 | 17.4 | 1.28 | 14.93 | 15.17 | 1.02 |
| 224811_at | LPP | Hs.5724.1 | Hs.5724 | LIM domain containing preferred translocation partner in lipoma | 335.36 | 406.09 | 1.21 | 309.26 | 318.52 | 1.03 |
| 224940_s_at | PAPPA | Hs.250655.4 | Hs.643599 | pregnancy-associated plasma protein A, pappalysin 1 | 10.31 | 12.78 | 1.24 | 13.15 | 11.77 | -1.12 |
| 225142_at | JHDM1D | Hs.222707.0 | Hs.308710 | jumonji C domain containing histone demethylase 1 homolog D (S. cerevisiae) | 47.06 | 58.66 | 1.25 | 53.75 | 55.55 | 1.03 |
| 225489_at | TMEM18 | Hs.43899.0 | Hs.43899 | transmembrane protein 18 | 193.45 | 150.69 | -1.28 | 179.4 | 173.5 | -1.03 |
| 225778_at | RBMS2 | Hs.20340.0 | Hs.505729 | RNA binding motif, single stranded interacting protein 2 | 39.77 | 47.88 | 1.2 | 36.93 | 35.26 | -1.05 |
| 225821_s_at | BOD1L1 | Hs.106204.0 | Hs.444517 | biorientation of chromosomes in cell division 1-like 1 | 191.39 | 251.27 | 1.31 | 206.04 | 181.75 | -1.13 |
| 225914_s_at | CAB39L | Hs.87159.0 | Hs.87159 | calcium binding protein 39-like | 11.26 | 9.02 | -1.25 | 9.77 | 9.44 | -1.04 |
| 225998_at | GAB1 | Hs.239706.1 | Hs.80720 | GRB2-associated binding protein 1 | 14.08 | 17.56 | 1.25 | 14.4 | 14.69 | 1.02 |
| 226081_at | LZIC | Hs.153959.0 | Hs.327252 | leucine zipper and CTNNBIP1 domain containing | 12.55 | 15.29 | 1.22 | 12.98 | 13.57 | 1.05 |
| 226183_at | GSK3B | Hs.90063.1 | Hs.445733 | glycogen synthase kinase 3 beta | 47.05 | 57.52 | 1.22 | 53.85 | 53.07 | -1.01 |
| 226223_at | PAWR | Hs.42683.0 | Hs.643130 | PRKC, apoptosis, WT1, regulator | 10.71 | 13.07 | 1.22 | 12.24 | 12.78 | 1.04 |
| 226364_at | HIP1 | Hs.38489.0 | Hs.329266 | huntingtin interacting protein 1 | 211.73 | 260.33 | 1.23 | 221.41 | 217.87 | -1.02 |
| 226578_s_at | DUSP1 | Hs.171695.1 | Hs.171695 | Dual specificity phosphatase 1 | 10.46 | 13.66 | 1.31 | 10.59 | 10.1 | -1.05 |
| 226688_at | C3orf23 | Hs.296401.0 | Hs.55131 | chromosome 3 open reading frame 23 | 13.79 | 16.75 | 1.21 | 14.21 | 13.79 | -1.03 |
| 226825_s_at | TMEM165 | Hs.236510.1 | Hs.479766 | transmembrane protein 165 | 46.02 | 58.69 | 1.28 | 46.08 | 49.14 | 1.07 |
| 227004_at | --- | Hs.158196.2 | Hs.696079 | --- | 12.45 | 16.13 | 1.3 | 12.09 | 11.66 | -1.04 |
| 227055_at | METTL7B | Hs.51483.0 | Hs.51483 | methyltransferase like 7B | 5.26 | 6.53 | 1.24 | 4.81 | 5.44 | 1.13 |
| 227300_at | TMEM119 | Hs.93135.0 | Hs.449718 | transmembrane protein 119 | 4.93 | 6.26 | 1.27 | 5.49 | 5.45 | -1.01 |
| 227326_at | MXRA7 | Hs.11924.0 | Hs.601108 | matrix-remodelling associated 7 | 39.24 | 29.36 | -1.34 | 37.11 | 32.12 | -1.16 |
| 227496_at | LOC253842 / | Hs.195161.0 | Hs.586460 | uncharacterized LOC253842 /// nuclear receptor subfamily 6, group A, member | 41.49 | 54.27 | 1.31 | 45.09 | 45.42 | 1.01 |
| 227777_at | --- | Hs.26434.1 | Hs.432548 | --- | 43.44 | 54.23 | 1.25 | 45.67 | 53.72 | 1.18 |
| 228062_at | NAP1L5 | Hs.12554.1 | Hs.12554 | nucleosome assembly protein 1-like 5 | 13.56 | 17.06 | 1.26 | 16.33 | 17.31 | 1.06 |
| 228085_at | LOC1005074 | Hs.166437.0 | Hs.715074 | uncharacterized LOC100507419 /// uncharacterized LOC100653076 | 13.69 | 16.69 | 1.22 | 16.03 | 13.72 | -1.17 |

| | | | | | | | | | | |
|---|---|---|---|---|---|---|---|---|---|---|
| 228116_at | --- | Hs.214410.0 | --- | --- | 22.15 | 18.08 | -1.22 | 20.17 | 20.36 | 1.01 |
| 228140_s_at | PPP2R2C | Hs.279909.1 | Hs.479069 | protein phosphatase 2, regulatory subunit B, gamma | 15.26 | 12.71 | -1.2 | 14.07 | 13.42 | -1.05 |
| 228272_at | DNLZ | Hs.135133.0 | Hs.528581 | DNL-type zinc finger | 60.12 | 46.66 | -1.29 | 50.17 | 58.42 | 1.16 |
| 228362_s_at | FAM26F | Hs.54277.1 | Hs.381220 | Family with sequence similarity 26, member F | 243.68 | 201.81 | -1.21 | 217.47 | 220.37 | 1.01 |
| 228566_at | RPRD1A | Hs.47044.0 | Hs.464912 | Regulation of nuclear pre-mRNA domain containing 1A | 42.21 | 51.27 | 1.21 | 51.99 | 44.5 | -1.17 |
| 228579_at | KCNQ3 | Hs.6407.0 | Hs.374023 | potassium voltage-gated channel, KQT-like subfamily, member 3 | 8.57 | 7.05 | -1.22 | 9.35 | 8.1 | -1.15 |
| 228749_at | ZDBF2 | Hs.28838.0 | Hs.110489 | zinc finger, DBF-type containing 2 | 8.85 | 10.73 | 1.21 | 10.99 | 10.17 | -1.08 |
| 228866_at | --- | Hs.125063.0 | Hs.659870 | --- | 62.46 | 75.03 | 1.2 | 65.57 | 63.4 | -1.03 |
| 228872_at | FAM108B1 | Hs.124831.1 | Hs.380389 | family with sequence similarity 108, member B1 | 7.88 | 6.56 | -1.2 | 7.12 | 7.41 | 1.04 |
| 228894_at | LOC253842 / | Hs.144054.0 | Hs.586460 | uncharacterized LOC253842 /// nuclear receptor subfamily 6, group A, member | 56.25 | 68.92 | 1.23 | 53.28 | 56.85 | 1.07 |
| 229005_at | MCTP2 | Hs.50742.0 | Hs.33368 | multiple C2 domains, transmembrane 2 | 127.63 | 153.57 | 1.2 | 125.47 | 127.17 | 1.01 |
| 229090_at | ZEB1-AS1 | Hs.97093.0 | Hs.372654 | ZEB1 antisense RNA 1 (non-protein coding) | 7.78 | 10.01 | 1.29 | 8.63 | 8.13 | -1.06 |
| 229094_at | ATP6V0E2-AS | Hs.6427.0 | --- | ATP6V0E2 antisense RNA 1 (non-protein coding) | 12.48 | 9.93 | -1.26 | 10.33 | 11.07 | 1.07 |
| 229128_s_at | ANP32E | Hs.71331.1 | Hs.656466 | Acidic (leucine-rich) nuclear phosphoprotein 32 family, member E | 12.55 | 19.38 | 1.55 | 16.41 | 14.32 | -1.15 |
| 229193_at | LUC7L3 | Hs.12653.0 | Hs.130293 | LUC7-like 3 (S. cerevisiae) | 42.95 | 31.8 | -1.35 | 35.11 | 37.29 | 1.06 |
| 229214_at | --- | Hs.152335.0 | Hs.733409 | --- | 65.14 | 80.33 | 1.23 | 80.08 | 78.9 | -1.02 |
| 229249_at | --- | Hs.24218.0 | Hs.389906 | --- | 60.66 | 80.72 | 1.33 | 62.67 | 70.06 | 1.12 |
| 229253_at | THEM4 | Hs.266155.0 | Hs.164070 | thioesterase superfamily member 4 | 358.23 | 293.56 | -1.22 | 351.86 | 372.73 | 1.06 |
| 229390_at | FAM26F | Hs.54277.2 | Hs.381220 | family with sequence similarity 26, member F | 788.64 | 635.77 | -1.24 | 664.6 | 704.97 | 1.06 |
| 229391_s_at | FAM26F | Hs.54277.2 | Hs.381220 | family with sequence similarity 26, member F | 633.17 | 513.29 | -1.23 | 560.86 | 564.9 | 1.01 |
| 229622_at | FAM132B | Hs.24951.0 | Hs.24951 | family with sequence similarity 132, member B | 17.75 | 14.75 | -1.2 | 15.53 | 15.77 | 1.02 |
| 229756_at | --- | Hs.128064.0 | Hs.659481 | --- | 12 | 14.41 | 1.2 | 13.48 | 15.52 | 1.15 |
| 229776_at | SLCO3A1 | Hs.124799.0 | Hs.311187 | solute carrier organic anion transporter family, member 3A1 | 33.86 | 41.48 | 1.23 | 34.86 | 32.27 | -1.08 |
| 229889_at | FAM211A | Hs.295362.1 | Hs.25425 | family with sequence similarity 211, member A | 53.2 | 64.15 | 1.21 | 55.83 | 66.46 | 1.19 |
| 230175_s_at | DCBLD2 | Hs.173374.0 | Hs.203691 | Discoidin, CUB and LCCL domain containing 2 | 73.81 | 88.92 | 1.2 | 77.71 | 85.14 | 1.1 |
| 230378_at | SCGB3A1 | Hs.62492.0 | Hs.62492 | secretoglobin, family 3A, member 1 | 34.68 | 28.64 | -1.21 | 26.82 | 26.66 | -1.01 |
| 230516_at | MALSU1 | Hs.87385.1 | Hs.87385 | Mitochondrial assembly of ribosomal large subunit 1 | 54.29 | 65.3 | 1.2 | 60.22 | 67.76 | 1.13 |
| 230736_at | LOC387647 | Hs.110835.0 | Hs.740552 | patched domain containing 3 pseudogene | 39.65 | 47.59 | 1.2 | 41.97 | 43.17 | 1.03 |
| 230902_at | --- | Hs.4254.0 | Hs.719922 | --- | 13.67 | 17.42 | 1.27 | 16.47 | 19.57 | 1.19 |
| 231068_at | SLC47A2 | Hs.126830.0 | Hs.126830 | solute carrier family 47, member 2 | 8.47 | 6.99 | -1.21 | 7.72 | 7.26 | -1.06 |
| 231236_at | ZFP57 | Hs.156326.0 | Hs.156326 | zinc finger protein 57 homolog (mouse) | 34.35 | 53.05 | 1.54 | 37.19 | 41.8 | 1.12 |
| 231311_at | --- | Hs.120451.0 | Hs.634674 | --- | 5.75 | 4.62 | -1.24 | 5.43 | 5.23 | -1.04 |
| 231497_at | ZBTB20-AS1 | Hs.236816.0 | Hs.122417 | ZBTB20 antisense RNA 1 (non-protein coding) | 28.96 | 23.82 | -1.22 | 24.84 | 26.86 | 1.08 |
| 231555_at | --- | Hs.7041.1 | Hs.664835 | --- | 10.95 | 13.3 | 1.21 | 10.16 | 9.44 | -1.08 |
| 231557_at | --- | Hs.61960.1 | Hs.602583 // | --- | 12.73 | 15.73 | 1.24 | 12.93 | 14.56 | 1.13 |
| 231963_at | ANKRD33B | Hs.26039.0 | Hs.26039 | ankyrin repeat domain 33B | 20.41 | 24.72 | 1.21 | 21.17 | 19.33 | -1.1 |
| 232087_at | CXorf23 | Hs.28896.0 | Hs.28896 | chromosome X open reading frame 23 | 7.66 | 10.48 | 1.37 | 9.61 | 9.97 | 1.04 |
| 232109_at | UBXN10 | Hs.202473.0 | Hs.432503 | UBX domain protein 10 | 11.88 | 9.24 | -1.29 | 11.97 | 11.51 | -1.04 |
| 232156_at | --- | Hs.135570.0 | Hs.720252 | --- | 20.52 | 27.9 | 1.36 | 23.19 | 26.52 | 1.14 |
| 232259_s_at | LOC1000096 | Hs.301956.2 | Hs.740439 | uncharacterized LOC100009676 | 34.27 | 28.41 | -1.21 | 26.68 | 29.47 | 1.1 |
| 232310_at | LOC1005076 | Hs.4786.0 | Hs.642767 | uncharacterized LOC100507637 | 19.54 | 15.89 | -1.23 | 19.04 | 17.36 | -1.1 |
| 232421_at | SCARB1 | Hs.287283.0 | Hs.731377 | scavenger receptor class B, member 1 | 18.21 | 25.25 | 1.39 | 23.78 | 24.56 | 1.03 |
| 232522_at | --- | Hs.288565.0 | Hs.733864 | --- | 31.05 | 40.7 | 1.31 | 28.11 | 28.43 | 1.01 |
| 232530_at | PLD1 | Hs.212533.0 | Hs.382865 | phospholipase D1, phosphatidylcholine-specific | 17.04 | 21.36 | 1.25 | 16.57 | 14.71 | -1.13 |
| 232539_at | --- | Hs.24022.0 | Hs.594374 | --- | 13.74 | 11.23 | -1.22 | 14.21 | 14.67 | 1.03 |
| 232691_at | SFXN5 | Hs.165814.0 | Hs.368171 | sideroflexin 5 | 7.39 | 6.12 | -1.21 | 7.74 | 7.02 | -1.1 |
| 232800_at | --- | Hs.153272.0 | Hs.534619 | --- | 10.83 | 8.73 | -1.24 | 10.98 | 9.47 | -1.16 |
| 232866_at | ZSCAN18 | Hs.235390.2 | Hs.235390 | zinc finger and SCAN domain containing 18 | 18.56 | 15 | -1.24 | 16.71 | 15.41 | -1.08 |
| 233099_at | --- | Hs.283859.0 | Hs.676447 | --- | 22.75 | 29.22 | 1.28 | 22.14 | 25.25 | 1.14 |
| 233134_at | RPH3AL | Hs.198551.2 | Hs.651925 | rabphilin 3A-like (without C2 domains) | 11.63 | 9.17 | -1.27 | 9.76 | 10.43 | 1.07 |
| 233217_at | --- | Hs.283934.0 | Hs.606581 | --- | 1350.15 | 1686.46 | 1.25 | 1163.42 | 1241.2 | 1.07 |
| 233236_at | TSPAN16 | Hs.283934.0 | Hs.579784 | tetraspanin 16 | 763.36 | 952.54 | 1.25 | 621.12 | 689.44 | 1.11 |
| 233713_at | --- | Hs.306627.0 | Hs.660880 | --- | 19.36 | 15.97 | -1.21 | 19.38 | 20.54 | 1.06 |

| | | | | | | | | | | |
|---|---|---|---|---|---|---|---|---|---|---|
| 233799_at | --- | Hs.296647.0 | Hs.677036 | --- | 15.53 | 12.5 | -1.24 | 15.16 | 14.57 | -1.04 |
| 233824_at | --- | Hs.287663.0 | Hs.668226 | --- | 22.3 | 27.27 | 1.22 | 19.52 | 21.79 | 1.12 |
| 233867_at | --- | Hs.272229.0 | Hs.610960 | --- | 309.83 | 380.84 | 1.23 | 288.04 | 284.79 | -1.01 |
| 233903_s_at | ARHGEF26 | Hs.240845.0 | Hs.240845 | Rho guanine nucleotide exchange factor (GEF) 26 | 18.3 | 21.98 | 1.2 | 20.01 | 18.53 | -1.08 |
| 234115_s_at | ZNRD1 | Hs.302037.0 | Hs.57813 | zinc ribbon domain containing 1 | 18.94 | 15.77 | -1.2 | 19.51 | 18.45 | -1.06 |
| 234196_at | --- | Hs.306749.0 | Hs.677313 | --- | 43.42 | 55.65 | 1.28 | 45.29 | 41.2 | -1.1 |
| 234322_at | --- | Hs.190327.0 | Hs.677289 | --- | 9.21 | 11.22 | 1.22 | 8.69 | 8.77 | 1.01 |
| 234397_at | --- | Hs.258613.0 | --- | --- | 12.33 | 9.96 | -1.24 | 10.56 | 10.19 | -1.04 |
| 234562_x_at | --- | Hs.306381.0 | Hs.675414 | --- | 558.53 | 453.74 | -1.23 | 564.94 | 505.39 | -1.12 |
| 234664_at | LOC1001320 | Hs.326048.3 | Hs.732199 | uncharacterized LOC100132062 /// uncharacterized LOC100288102 /// unchara | 53.78 | 65.1 | 1.21 | 57.59 | 58.21 | 1.01 |
| 235250_at | FLCN | Hs.172702.0 | Hs.31652 | folliculin | 23.45 | 29.59 | 1.26 | 30.11 | 31.81 | 1.06 |
| 235289_at | EIF5A2 | Hs.164144.0 | Hs.164144 | eukaryotic translation initiation factor 5A2 | 22.69 | 18.33 | -1.24 | 24.57 | 22.74 | -1.08 |
| 235334_at | ST6GALNAC3 | Hs.85618.0 | Hs.735443 | ST6 (alpha-N-acetyl-neuraminyl-2,3-beta-galactosyl-1,3)-N-acetylgalactosamini | 8.33 | 10.74 | 1.29 | 10.38 | 9.44 | -1.1 |
| 235542_at | TET3 | Hs.118047.0 | Hs.516107 | tet methylcytosine dioxygenase 3 | 74.1 | 95.76 | 1.29 | 69.5 | 73.39 | 1.06 |
| 235575_at | --- | Hs.166271.0 | Hs.715879 | --- | 62.11 | 45.63 | -1.36 | 68.2 | 67.35 | -1.01 |
| 235628_x_at | FLJ33630 | Hs.138531.0 | Hs.340623 | uncharacterized LOC644873 | 19.07 | 15.62 | -1.22 | 18.21 | 16.37 | -1.11 |
| 235680_at | --- | Hs.222240.0 | --- | --- | 53.01 | 64.75 | 1.22 | 53.98 | 46.34 | -1.16 |
| 235701_at | --- | Hs.132947.0 | Hs.660986 | --- | 53.96 | 67.25 | 1.25 | 53.31 | 47.16 | -1.13 |
| 235719_at | CYP4V2 | Hs.99237.0 | Hs.587231 | cytochrome P450, family 4, subfamily V, polypeptide 2 | 28.27 | 22.5 | -1.26 | 26.6 | 24.25 | -1.1 |
| 235802_at | PLD4 | Hs.136896.0 | Hs.407101 | phospholipase D family, member 4 | 32.94 | 41.37 | 1.26 | 37.37 | 35.08 | -1.07 |
| 235920_at | --- | Hs.19613.0 | --- | --- | 196.23 | 150.62 | -1.3 | 170.49 | 160.73 | -1.06 |
| 235993_at | PSMF1 | Hs.33431.0 | Hs.471917 | proteasome (prosome, macropain) inhibitor subunit 1 (PI31) | 58.37 | 73.14 | 1.25 | 68.92 | 67.84 | -1.02 |
| 236063_at | --- | Hs.159188.0 | Hs.600017 | --- | 86.72 | 104.4 | 1.2 | 85.78 | 89.06 | 1.04 |
| 236092_at | --- | Hs.125829.0 | Hs.125829 | --- | 14.54 | 11.91 | -1.22 | 11.63 | 13.38 | 1.15 |
| 236094_at | TCF7L2 | Hs.14931.0 | Hs.593995 | Transcription factor 7-like 2 (T-cell specific, HMG-box) | 45.44 | 55.1 | 1.21 | 43.06 | 39.11 | -1.1 |
| 236211_at | RAB40B | Hs.50925.0 | Hs.484068 | RAB40B, member RAS oncogene family | 13.81 | 17.31 | 1.25 | 13.61 | 14.62 | 1.07 |
| 236370_at | --- | Hs.13905.0 | Hs.655809 | --- | 46.44 | 58.31 | 1.26 | 49.11 | 46.78 | -1.05 |
| 236398_s_at | --- | Hs.169898.0 | Hs.520684 | --- | 127.1 | 157.43 | 1.24 | 132.24 | 154.26 | 1.17 |
| 236417_at | --- | Hs.135624.0 | Hs.657666 | --- | 138.27 | 165.98 | 1.2 | 145.39 | 131.87 | -1.1 |
| 236859_at | RUNX2 | Hs.122116.0 | Hs.535845 | runt-related transcription factor 2 | 18.68 | 14.47 | -1.29 | 18.42 | 17.37 | -1.06 |
| 236929_at | LOC441242 | Hs.49051.0 | Hs.373941 | Uncharacterized LOC441242 | 9.84 | 8.08 | -1.22 | 9.78 | 8.77 | -1.11 |
| 236988_x_at | ITGB2 | Hs.323138.0 | Hs.375957 | integrin, beta 2 (complement component 3 receptor 3 and 4 subunit) | 101.43 | 170.78 | 1.68 | 102.24 | 106.79 | 1.04 |
| 237136_at | --- | Hs.252813.0 | Hs.672118 | --- | 16.29 | 19.67 | 1.21 | 19.73 | 16.61 | -1.19 |
| 237315_at | --- | Hs.149264.0 | --- | --- | 67.74 | 86.99 | 1.28 | 80.75 | 71.32 | -1.13 |
| 237905_at | KRT25 | Hs.55412.0 | Hs.55412 | keratin 25 | 9.46 | 7.86 | -1.2 | 9.37 | 8.71 | -1.08 |
| 237909_at | ADAM6 | Hs.97508.0 | Hs.662288 | ADAM metallopeptidase domain 6 (pseudogene) | 14.15 | 11.63 | -1.22 | 13.01 | 13.07 | 1 |
| 238243_at | --- | Hs.185268.0 | Hs.657662 | --- | 13.24 | 16.08 | 1.21 | 12.88 | 12.39 | -1.04 |
| 238449_at | LOC595101 | Hs.331640.0 | Hs.654650 | Smg-1 homolog, phosphatidylinositol 3-kinase-related kinase (C. elegans) pseud | 60.16 | 74.84 | 1.24 | 63.28 | 54.33 | -1.16 |
| 238452_at | FCRLB | Hs.164495.0 | Hs.517422 | Fc receptor-like B | 14.82 | 18.23 | 1.23 | 16.79 | 18.53 | 1.1 |
| 238485_at | IQCH-AS1 | Hs.193236.0 | Hs.656245 | IQCH antisense RNA 1 (non-protein coding) | 46.1 | 57.23 | 1.24 | 65.22 | 63.07 | -1.03 |
| 238581_at | GBP5 | Hs.211535.0 | Hs.513726 | guanylate binding protein 5 | 150.31 | 203.56 | 1.35 | 129.5 | 144.87 | 1.12 |
| 238800_s_at | ZCCHC6 | Hs.102682.0 | Hs.597057 | Zinc finger, CCHC domain containing 6 | 212.99 | 256.99 | 1.21 | 202.36 | 192.08 | -1.05 |
| 239032_at | --- | Hs.130835.0 | Hs.605110 | --- | 19.48 | 23.45 | 1.2 | 22.94 | 20.47 | -1.12 |
| 239124_at | --- | Hs.18920.0 | Hs.656273 | --- | 146.83 | 180.44 | 1.23 | 161.98 | 158.46 | -1.02 |
| 239269_at | LOC1006530 | Hs.200577.0 | Hs.523789 | uncharacterized LOC100653017 /// microRNA 612 /// nuclear paraspeckle asser | 13.15 | 17.53 | 1.33 | 15 | 15.76 | 1.05 |
| 239364_at | --- | Hs.293972.1 | --- | --- | 24.53 | 30.94 | 1.26 | 22.53 | 19.64 | -1.15 |
| 239413_at | CEP152 | Hs.50500.0 | Hs.597323 | centrosomal protein 152kDa | 20.16 | 26.2 | 1.3 | 27.4 | 25.39 | -1.08 |
| 239453_at | --- | Hs.191534.0 | Hs.673408 | --- | 43.18 | 57.03 | 1.32 | 47.47 | 50.07 | 1.05 |
| 239701_at | --- | Hs.199310.0 | Hs.661976 | --- | 78.89 | 100.09 | 1.27 | 73.96 | 69.57 | -1.06 |
| 239758_at | --- | Hs.26125.0 | --- | --- | 16.86 | 21.18 | 1.26 | 18.92 | 17.93 | -1.06 |
| 239777_at | C14orf182 | Hs.296320.1 | Hs.660867 | chromosome 14 open reading frame 182 | 27.96 | 22.61 | -1.24 | 25.5 | 24.32 | -1.05 |
| 239809_at | --- | Hs.161333.0 | Hs.659380 | --- | 80.69 | 109.66 | 1.36 | 92.73 | 88.18 | -1.05 |
| 239902_at | --- | Hs.46517.0 | --- | --- | 34.04 | 41.1 | 1.21 | 39.23 | 41.45 | 1.06 |

| | | | | | | | | | | |
|---|---|---|---|---|---|---|---|---|---|---|
| 240019_at | --- | Hs.271305.0 | --- | --- | 171.57 | 237.61 | 1.38 | 300.95 | 294.64 | -1.02 |
| 240103_at | --- | Hs.158152.0 | Hs.602901 | --- | 15.59 | 21.25 | 1.36 | 19.94 | 18.94 | -1.05 |
| 240207_at | --- | Hs.117308.0 | Hs.664722 | --- | 11.12 | 9.07 | -1.23 | 8.75 | 9.29 | 1.06 |
| 240358_at | --- | Hs.97296.0 | Hs.625321 | --- | 80.9 | 97.57 | 1.21 | 87.26 | 80.01 | -1.09 |
| 240744_at | CPA5 | Hs.144699.0 | Hs.567642 | carboxypeptidase A5 | 26.47 | 16.73 | -1.58 | 19.3 | 21.55 | 1.12 |
| 240793_at | TTN | Hs.213042.0 | Hs.134602 | Titin | 52.29 | 65.67 | 1.26 | 54.6 | 61.93 | 1.13 |
| 240845_at | --- | Hs.25296.0 | Hs.656972 | --- | 20.2 | 25.28 | 1.25 | 22.63 | 23.29 | 1.03 |
| 240872_at | --- | Hs.262643.0 | Hs.262643 | --- | 15.02 | 12.05 | -1.25 | 15.33 | 14.08 | -1.09 |
| 241184_x_at | --- | Hs.32888.0 | --- | --- | 7.67 | 6.33 | -1.21 | 6.47 | 6.7 | 1.04 |
| 241205_at | --- | Hs.132697.0 | --- | --- | 28.05 | 34.26 | 1.22 | 29.93 | 28.14 | -1.06 |
| 241599_at | LSM11 | Hs.187117.0 | Hs.631954 | LSM11, U7 small nuclear RNA associated | 11.05 | 9.17 | -1.2 | 10.76 | 9.39 | -1.15 |
| 241788_x_at | --- | Hs.270998.0 | --- | --- | 10.01 | 14.21 | 1.42 | 10.72 | 10.4 | -1.03 |
| 241813_at | MBD1 | Hs.214140.0 | Hs.405610 | methyl-CpG binding domain protein 1 | 16.19 | 19.84 | 1.23 | 18.32 | 18.4 | 1 |
| 242075_at | --- | Hs.158209.0 | Hs.655948 | --- | 26.5 | 36.28 | 1.37 | 24.8 | 20.93 | -1.19 |
| 242197_x_at | CD36 | Hs.248425.0 | Hs.120949 | CD36 molecule (thrombospondin receptor) | 185.16 | 222.58 | 1.2 | 180.38 | 181.08 | 1 |
| 242295_at | FLJ32955 | Hs.123533.0 | Hs.470217 | uncharacterized protein FLJ32955 | 10.26 | 12.47 | 1.22 | 11.45 | 11.39 | -1 |
| 242408_at | STYX | Hs.105219.0 | Hs.364980 | serine/threonine/tyrosine interacting protein | 11.93 | 9.84 | -1.21 | 10.83 | 11.39 | 1.05 |
| 242425_at | --- | Hs.257266.0 | Hs.661595 | --- | 75.13 | 94.69 | 1.26 | 77.18 | 72.27 | -1.07 |
| 242543_at | SH2D6 | Hs.209542.0 | Hs.209542 | SH2 domain containing 6 | 18.74 | 15.51 | -1.21 | 17.49 | 15.82 | -1.11 |
| 242582_at | --- | Hs.156918.0 | --- | --- | 39.65 | 51.28 | 1.29 | 46.26 | 47.6 | 1.03 |
| 242612_at | --- | Hs.208800.0 | Hs.663718 | --- | 54.33 | 65.93 | 1.21 | 50.41 | 53.04 | 1.05 |
| 242642_at | --- | Hs.226717.0 | --- | --- | 75.77 | 62.88 | -1.2 | 68.46 | 63.24 | -1.08 |
| 242683_at | LOC400643 | Hs.253920.0 | Hs.253920 | uncharacterized LOC400643 | 11.21 | 9.29 | -1.21 | 9.92 | 9.78 | -1.01 |
| 242729_at | LOC1005068 | Hs.166999.0 | Hs.710694 | Uncharacterized LOC100506866 | 20.84 | 25.76 | 1.24 | 20.27 | 21.99 | 1.08 |
| 242738_s_at | ZFHX3 | Hs.163208.0 | Hs.598297 | zinc finger homeobox 3 | 11.23 | 9.25 | -1.21 | 11.17 | 9.99 | -1.12 |
| 242905_at | PNO1 | Hs.262858.0 | Hs.262858 | partner of NOB1 homolog (S. cerevisiae) | 11.96 | 14.67 | 1.23 | 12.16 | 14.19 | 1.17 |
| 243149_at | --- | Hs.173696.0 | Hs.669156 | --- | 6.19 | 8.16 | 1.32 | 6.28 | 6.3 | 1 |
| 243194_at | ZNF551 | Hs.308199.0 | Hs.109540 | zinc finger protein 551 | 20.47 | 16.82 | -1.22 | 18.2 | 18.2 | 1 |
| 243473_at | --- | Hs.269210.0 | --- | --- | 18.22 | 23.1 | 1.27 | 19.02 | 17.26 | -1.1 |
| 243534_at | CC2D2B /// L | Hs.112971.0 | Hs.538374 | coiled-coil and C2 domain containing 2B /// coiled-coil and C2 domain-containin | 17.13 | 21.88 | 1.28 | 18.28 | 21.08 | 1.15 |
| 243641_at | --- | Hs.279643.0 | Hs.660050 | --- | 16.39 | 13.21 | -1.24 | 14.17 | 15.77 | 1.11 |
| 243683_at | MORF4L2 | Hs.192905.0 | Hs.326387 | Mortality factor 4 like 2 | 30.9 | 25.7 | -1.2 | 23.8 | 21.25 | -1.12 |
| 243764_at | VSIG1 | Hs.177164.0 | Hs.177164 | V-set and immunoglobulin domain containing 1 | 50.03 | 40.41 | -1.24 | 46.21 | 48.68 | 1.05 |
| 243824_at | --- | Hs.46765.0 | Hs.351215 | --- | 211.51 | 256.78 | 1.21 | 334.93 | 291.02 | -1.15 |
| 243843_at | N4BP2L1 | Hs.152269.0 | Hs.732118 | NEDD4 binding protein 2-like 1 | 42.19 | 50.65 | 1.2 | 46.29 | 48.25 | 1.04 |
| 243859_at | --- | Hs.250488.0 | Hs.604501 | --- | 10.67 | 8.83 | -1.21 | 9.46 | 9.13 | -1.04 |
| 244072_at | --- | Hs.131237.0 | --- | --- | 13.03 | 10.73 | -1.21 | 12.06 | 11.44 | -1.05 |
| 244144_at | SYNE1 | Hs.192102.0 | Hs.12967 | spectrin repeat containing, nuclear envelope 1 | 36.83 | 48.43 | 1.31 | 41.16 | 36.99 | -1.11 |
| 244280_at | LOC1005072 | Hs.103070.0 | Hs.103070 | uncharacterized LOC100507254 | 12.67 | 16.45 | 1.3 | 14.37 | 14.53 | 1.01 |
| 244292_at | --- | Hs.132759.0 | Hs.633769 | --- | 47 | 56.75 | 1.21 | 51.58 | 48.4 | -1.07 |
| 244297_at | ANKRD18A | Hs.169001.0 | Hs.644630 | ankyrin repeat domain 18A | 22.81 | 27.95 | 1.23 | 28.88 | 26 | -1.11 |
| 244366_at | LOC1005063 | Hs.192269.0 | --- | uncharacterized LOC100506371 | 11.26 | 9.3 | -1.21 | 10.3 | 9.84 | -1.05 |
| 244548_at | --- | Hs.120915.0 | Hs.658232 | --- | 779.55 | 953.2 | 1.22 | 692.59 | 617.67 | -1.12 |
| 244679_at | --- | Hs.171618.0 | --- | --- | 48.45 | 59.92 | 1.24 | 44.56 | 41.45 | -1.07 |
| 244721_at | TP53INP1 | Hs.253962.0 | Hs.492261 | tumor protein p53 inducible nuclear protein 1 | 8.9 | 7.19 | -1.24 | 7.41 | 7.85 | 1.06 |
| 244766_at | 61E3.4 /// LO | Hs.293645.0 | Hs.723071 | nuclear pore complex interacting protein-like /// nuclear pore complex interact | 17.81 | 21.45 | 1.2 | 21.12 | 19.3 | -1.09 |
| 244860_at | --- | Hs.257683.0 | Hs.610342 | --- | 10.7 | 13.56 | 1.27 | 10.28 | 10.82 | 1.05 |
| 244868_at | --- | Hs.17263.0 | --- | --- | 51.18 | 66.23 | 1.29 | 64.77 | 56.5 | -1.15 |
| 244886_at | LOC389641 | Hs.178670.0 | Hs.591835 | uncharacterized LOC389641 | 61.52 | 47.7 | -1.29 | 57.43 | 62.39 | 1.09 |
| 1552334_at | TRIOBP | Hs2.40342.1 | Hs.533030 | TRIO and F-actin binding protein | 71.09 | 88.06 | 1.24 | 73.33 | 79.14 | 1.08 |
| 1552737_s_a | WWP2 | Hs2.333382. | Hs.408458 | WW domain containing E3 ubiquitin protein ligase 2 | 32.02 | 40.84 | 1.28 | 32.19 | 36.25 | 1.13 |
| 1552787_at | HELB | Hs2.192323. | Hs.505941 | helicase (DNA) B | 37 | 28.59 | -1.29 | 29.93 | 32.61 | 1.09 |
| 1552797_s_a | PROM2 | Hs2.145582. | Hs.469313 | prominin 2 | 18.36 | 22.15 | 1.21 | 22.65 | 22.43 | -1.01 |

| | | | | | | | | | | |
|---|---|---|---|---|---|---|---|---|---|---|
| 1552908_at | C1orf150 | Hs2.352641. | Hs.732113 | chromosome 1 open reading frame 150 | 12.58 | 10.32 | -1.22 | 11.65 | 11.16 | -1.04 |
| 1553061_at | OR6W1P | Hs2.339818. | Hs.339818 | olfactory receptor, family 6, subfamily W, member 1 pseudogene | 6.43 | 5.3 | -1.21 | 5.35 | 6.1 | 1.14 |
| 1553701_a_ | DUSP18 | Hs2.128782. | Hs.517544 | dual specificity phosphatase 18 | 18.62 | 15.37 | -1.21 | 16.92 | 15.72 | -1.08 |
| 1553805_at | C3orf49 | Hs2.278116. | Hs.506386 | chromosome 3 open reading frame 49 | 13.05 | 10.61 | -1.23 | 10.88 | 11.93 | 1.1 |
| 1553959_a_ | B3GALT6 | Hs2.284284. | Hs.284284 | UDP-Gal:betaGal beta 1,3-galactosyltransferase polypeptide 6 | 33.11 | 26.99 | -1.23 | 31.5 | 36.3 | 1.15 |
| 1553970_s_ | CEL | Hs2.406160. | Hs.533258 | carboxyl ester lipase (bile salt-stimulated lipase) | 9.72 | 7.63 | -1.27 | 9.29 | 9.36 | 1.01 |
| 1554384_at | PADI2 | Hs2.33455.2 | Hs.33455 | peptidyl arginine deiminase, type II | 23.99 | 31.92 | 1.33 | 28 | 26.67 | -1.05 |
| 1554385_a_ | PADI2 | Hs2.33455.2 | Hs.33455 | peptidyl arginine deiminase, type II | 38.29 | 50.54 | 1.32 | 43.89 | 40.56 | -1.08 |
| 1554424_at | FIP1L1 | Hs2.334812. | Hs.624245 | FIP1 like 1 (S. cerevisiae) | 18.21 | 24.61 | 1.35 | 20.19 | 19.71 | -1.02 |
| 1555294_a_ | ERC1 | Hs2.293705. | Hs.601216 | ELKS/RAB6-interacting/CAST family member 1 | 10.72 | 8.82 | -1.22 | 10.56 | 9.26 | -1.14 |
| 1554606_at | CEP120 | Hs2.42673.2 | Hs.483209 | centrosomal protein 120kDa | 6.1 | 7.35 | 1.21 | 7.01 | 6.84 | -1.03 |
| 1554710_at | KCNMB1 | Hs2.93841.3 | Hs.484099 | potassium large conductance calcium-activated channel, subfamily M, beta me | 22.11 | 27.91 | 1.26 | 20.37 | 23.04 | 1.13 |
| 1554806_a_ | FBXO8 | Hs2.76917.2 | Hs.76917 | F-box protein 8 | 15.97 | 19.68 | 1.23 | 19.54 | 16.77 | -1.17 |
| 1554833_at | MCTP2 | Hs2.33368.2 | Hs.33368 | multiple C2 domains, transmembrane 2 | 16.22 | 20.25 | 1.25 | 18.82 | 19.56 | 1.04 |
| 1555007_s_ | WDR66 | Hs2.356828. | Hs.709837 | WD repeat domain 66 | 6.98 | 8.39 | 1.2 | 8.12 | 6.95 | -1.17 |
| 1555363_s_ | LOC284440 | Hs2.255021. | Hs.665307 | uncharacterized LOC284440 | 17.5 | 13.58 | -1.29 | 19.34 | 17.4 | -1.11 |
| 1555374_at | --- | Hs2.6127.2 | --- | --- | 20.59 | 17.09 | -1.2 | 19.13 | 18.91 | -1.01 |
| 1555441_at | UBA6 | Hs2.59838.2 | Hs.212774 | ubiquitin-like modifier activating enzyme 6 | 13.13 | 10.34 | -1.27 | 11.76 | 10.67 | -1.1 |
| 1555749_at | SF1 | Hs2.382653. | Hs.502829 | splicing factor 1 | 32.94 | 46.3 | 1.41 | 30.97 | 33.07 | 1.07 |
| 1555819_s_ | SAMD14 | Hs2.106890. | Hs.567769 | sterile alpha motif domain containing 14 | 25.64 | 20.26 | -1.27 | 24.27 | 24.58 | 1.01 |
| 1555996_s_ | EIF4A2 /// M | Hs2.410186. | Hs.518475 | eukaryotic translation initiation factor 4A2 /// microRNA 1248 /// small nucleol | 7.61 | 9.5 | 1.25 | 8.87 | 8.45 | -1.05 |
| 1556004_at | LOC1005067 | Hs2.213950. | --- | uncharacterized LOC100506777 | 26.91 | 33.19 | 1.23 | 25.04 | 25.22 | 1.01 |
| 1556103_at | --- | Hs2.323409. | Hs.614375 // | --- | 43.18 | 55.14 | 1.28 | 54.68 | 54.05 | -1.01 |
| 1556175_at | MTSS1L | Hs2.355835. | Hs.432387 | metastasis suppressor 1-like | 85.45 | 107.57 | 1.26 | 105.47 | 96.08 | -1.1 |
| 1556352_at | --- | Hs2.421612. | Hs.579960 | --- | 84.28 | 107.66 | 1.28 | 85.05 | 86.52 | 1.02 |
| 1556896_at | LOC284751 | Hs2.282325. | Hs.282325 | uncharacterized LOC284751 | 26.76 | 46.03 | 1.72 | 29.77 | 29.36 | -1.01 |
| 1556958_at | --- | Hs2.344219. | Hs.551095 | --- | 15.51 | 12.57 | -1.23 | 14.71 | 15.6 | 1.06 |
| 1557036_at | ZBTB1 | Hs2.400802. | Hs.605143 | Zinc finger and BTB domain containing 1 | 19.58 | 15.66 | -1.25 | 19.79 | 16.82 | -1.18 |
| 1557195_at | --- | Hs2.153652. | Hs.153652 | --- | 43.27 | 34.73 | -1.25 | 48.39 | 50.01 | 1.03 |
| 1557415_s_ | LETM2 | Hs2.369065. | Hs.734005 | leucine zipper-EF-hand containing transmembrane protein 2 | 20 | 24.19 | 1.21 | 22.33 | 25.07 | 1.12 |
| 1557437_a_ | --- | Hs2.271478. | Hs.655836 | --- | 16.56 | 13.14 | -1.26 | 18.93 | 20.88 | 1.1 |
| 1557626_at | --- | Hs2.406987. | Hs.678911 | --- | 52.29 | 62.9 | 1.2 | 52.06 | 53.83 | 1.03 |
| 1557630_s_ | --- | Hs2.331219. | Hs.585593 | --- | 87.29 | 70.75 | -1.23 | 74.68 | 69.47 | -1.08 |
| 1561728_a_ | LOC400238 | Hs2.131037. | Hs.131037 | uncharacterized LOC400238 | 9.85 | 7.45 | -1.32 | 8.61 | 9.28 | 1.08 |
| 1557987_at | --- | Hs2.374403. | Hs.623973 | --- | 22.83 | 28.3 | 1.24 | 26.51 | 27.12 | 1.02 |
| 1558356_at | UACA | Hs2.406900. | Hs.108049 | uveal autoantigen with coiled-coil domains and ankyrin repeats | 8.26 | 6.86 | -1.2 | 8.47 | 7.7 | -1.1 |
| 1558371_a_ | --- | Hs2.231992. | Hs.663124 | --- | 237.53 | 287.43 | 1.21 | 249.82 | 268.04 | 1.07 |
| 1558573_at | MCTS1 | Hs2.292922. | Hs.102696 | malignant T cell amplified sequence 1 | 8.91 | 7.14 | -1.25 | 7.49 | 7.85 | 1.05 |
| 1558592_at | --- | Hs2.228431. | Hs.664234 | --- | 26.78 | 32.56 | 1.22 | 26.22 | 30.3 | 1.16 |
| 1558787_a_ | TSPAN3 | Hs2.302809. | Hs.740392 | Tetraspanin 3 | 42.32 | 50.9 | 1.2 | 38.8 | 38.45 | -1.01 |
| 1558822_at | --- | Hs2.396929. | Hs.684614 | --- | 54.05 | 66.96 | 1.24 | 59.29 | 56.15 | -1.06 |
| 1559249_at | ATXN1 | Hs2.396468. | Hs.434961 | ataxin 1 | 35.76 | 42.92 | 1.2 | 33.32 | 31.62 | -1.05 |
| 1559362_at | --- | Hs2.407647. | Hs.656873 | --- | 12.23 | 14.99 | 1.23 | 12.9 | 13.05 | 1.01 |
| 1560119_at | LOC389634 | Hs2.252705. | Hs.434403 | uncharacterized LOC389634 | 25.43 | 20.6 | -1.23 | 25.13 | 23.87 | -1.05 |
| 1560492_at | --- | Hs2.348321. | Hs.680376 | --- | 33 | 40.08 | 1.21 | 34.62 | 33.46 | -1.03 |
| 1560625_s_ | --- | Hs2.385746. | Hs.512386 | --- | 34.5 | 42.53 | 1.23 | 43.57 | 47.95 | 1.1 |
| 1560750_at | LOC151121 | Hs2.351589. | Hs.531687 | uncharacterized LOC151121 | 11.45 | 9.47 | -1.21 | 11.75 | 10.07 | -1.17 |
| 1561307_at | --- | Hs2.438469. | Hs.570351 // | --- | 7.51 | 6.25 | -1.2 | 6.87 | 6.81 | -1.01 |
| 1561896_at | --- | Hs2.334578. | Hs.523913 | --- | 8.08 | 6.47 | -1.25 | 6.48 | 7.25 | 1.12 |
| 1562209_at | DCAF4L1 | Hs2.192619. | Hs.213307 | DDB1 and CUL4 associated factor 4-like 1 | 9.2 | 11.72 | 1.27 | 11.59 | 10.66 | -1.09 |
| 1562289_at | --- | Hs2.376781. | Hs.638554 | --- | 214.64 | 258.1 | 1.2 | 230.68 | 221.01 | -1.04 |
| 1562511_at | LYST | Hs2.376915. | Hs.532411 | lysosomal trafficking regulator | 43.92 | 53.8 | 1.22 | 44.04 | 45.7 | 1.04 |
| 1563209_a_ | MACROD2 | Hs2.399982. | Hs.661576 | MACRO domain containing 2 | 61.58 | 75.58 | 1.23 | 56.92 | 47.78 | -1.19 |

| | | | | | | | | | | |
|---|---|---|---|---|---|---|---|---|---|---|
| 1563466_at | MYLK | Hs2.407139.1 | Hs.477375 | myosin light chain kinase | 16.97 | 14.04 | -1.21 | 15.73 | 15.33 | -1.03 |
| 1563621_at | --- | Hs2.356887.1 | Hs.681809 | --- | 39.61 | 50.53 | 1.28 | 42.98 | 46.52 | 1.08 |
| 1563983_at | TPT1-AS1 | Hs2.375766.1 | Hs.375766 | TPT1 antisense RNA 1 (non-protein coding) | 9.39 | 11.55 | 1.23 | 10.26 | 11.06 | 1.08 |
| 1564580_at | --- | Hs2.375647.1 | Hs.671960 | --- | 12.83 | 10.16 | -1.26 | 12.7 | 12.11 | -1.05 |
| 1564936_at | --- | Hs2.383613.1 | Hs.545050 | --- | 10.72 | 13.8 | 1.29 | 11.56 | 12.6 | 1.09 |
| 1566037_at | DGCR7 | Hs2.381379.1 | Hs.657950 | DiGeorge syndrome critical region gene 7 | 29.23 | 36.38 | 1.24 | 31.97 | 36.21 | 1.13 |
| 1566176_at | --- | Hs2.356886.1 | --- | --- | 7.14 | 5.88 | -1.21 | 6.6 | 6.46 | -1.02 |
| 1566269_at | RALGAPA1 | Hs2.384657.1 | Hs.113150 | Ral GTPase activating protein, alpha subunit 1 (catalytic) | 6.97 | 8.46 | 1.21 | 8.32 | 7.88 | -1.06 |
| 1566633_at | --- | Hs2.375770.1 | Hs.544880 | --- | 30.07 | 24.18 | -1.24 | 28.17 | 27.03 | -1.04 |
| 1568836_at | CLK4 | Hs2.401054.1 | Hs.406557 | CDC-like kinase 4 | 6.65 | 8.01 | 1.2 | 7.44 | 6.97 | -1.07 |
| 1568846_at | --- | Hs2.397100.1 | Hs.684488 | --- | 32.2 | 25.3 | -1.27 | 29.67 | 28.3 | -1.05 |
| 1569149_at | PDLIM7 | Hs2.385816.1 | Hs.533040 | PDZ and LIM domain 7 (enigma) | 52.69 | 68.75 | 1.3 | 62.39 | 61.7 | -1.01 |
| 1569150_x_a | PDLIM7 | Hs2.385816.1 | Hs.533040 | PDZ and LIM domain 7 (enigma) | 74.25 | 94.26 | 1.27 | 81.86 | 81.44 | -1.01 |
| 1569156_at | ZNF846 | Hs2.389717.1 | Hs.665717 | zinc finger protein 846 | 24.12 | 17.73 | -1.36 | 27.58 | 28.84 | 1.05 |
| 1569408_at | EIF2C4 | Hs2.397392.1 | Hs.471492 | Eukaryotic translation initiation factor 2C, 4 | 10.93 | 13.55 | 1.24 | 11.3 | 10.99 | -1.03 |
| 1569538_at | --- | Hs2.383371.1 | --- | --- | 11.96 | 15.6 | 1.3 | 11.79 | 11.9 | 1.01 |
| 1569551_at | --- | Hs2.326103.1 | Hs.665748 | --- | 14.41 | 11.54 | -1.25 | 13.48 | 11.69 | -1.15 |
| 1569856_at | TPP2 | Hs2.399768.1 | Hs.432424 | tripeptidyl peptidase II | 31.49 | 25.17 | -1.25 | 28.58 | 30.95 | 1.08 |
| 1570302_at | --- | Hs2.382133.1 | Hs.711807 | --- | 10.4 | 8.65 | -1.2 | 8.68 | 9.96 | 1.15 |
| $ | 388 genes in file 'C:\Data\MA\EndoThelial\EndoThelial -Project_2013\Genelists\AnalysisResults\RES05-NRES05-Together.txt' satisfied the comparison filtering criteria | | | | | | | | | |

**Table S3**: List of genes whose expression is significantly correlated with blood pressure changes in hypertension BP responders.

**Table S3: List of genes whose expression is significantly correlated with blood pressure changes in hypertension BP responders.**

| Probes | Gene Symbol | Name | Correlation coefficient | Parametric p-value | Permutation p---value |
|---|---|---|---|---|---|
| 1552280_at | TIMD4 | T-cell immunoglobulin and mucin domain containing 4 | ---0.765 | 0.0058975 | 0.0059 |
| 1552787_at | HELB | helicase (DNA) B | 0.607 | 0.040002 | 0.0425 |
| 1552923_a_at | PITPNM2 | phosphatidylinositol transfer protein, membrane-associated 2 | ---0.702 | 0.0145393 | 0.0157 |
| 1553672_at | ENAH | enabled homolog (Drosophila) | 0.702 | 0.0145393 | 0.0127 |
| 1553970_s_at | CEL | carboxyl ester lipase | ---0.719 | 0.0110205 | 0.0117 |
| 1555071_at | TLL1 | tolloid like 1 | ---0.59 | 0.0488449 | 0.0477 |
| 1555142_at | SLC9B1 | solute carrier family 9, subfamily B (NHA1, cation proton antiporter 1), member 1 | ---0.614 | 0.0373345 | 0.0356 |
| 1555363_s_at | LINC00663 | long intergenic non-protein coding RNA 663 | ---0.635 | 0.0301143 | 0.0259 |
| 1555490_s_at | PDZD3 | PDZ domain containing 3 | ---0.695 | 0.0158777 | 0.015 |
| 1555741_at | MRAP | melanocortin 2 receptor accessory protein | 0.649 | 0.0259115 | 0.0264 |
| 1555884_at | PSMD6 | proteasome 26S subunit, non-ATPase 6 | 0.807 | 0.0027461 | 0.0024 |
| 1556097_at | HOMER2 | homer scaffolding protein 2 | 0.74 | 0.0081706 | 0.0082 |
| 1557415_s_at | LETM2 | leucine zipper-EF-hand containing transmembrane protein 2 | 0.674 | 0.0204419 | 0.0203 |
| 1557811_a_at | --- | --- | 0.586 | 0.0488449 | 0.0498 |
| 1558256_at | LINC00662 | long intergenic non-protein coding RNA 662 | 0.744 | 0.0081706 | 0.0084 |
| 1558778_s_at | MKL2 | MKL/myocardin-like 2 | ---0.737 | 0.0090521 | 0.0071 |
| 1558890_at | FAM53A | family with sequence similarity 53, member A | ---0.691 | 0.0158777 | 0.0156 |
| 1560453_at | --- | --- | ---0.604 | 0.0428069 | 0.0474 |
| 1562367_at | C15orf54 | chromosome 15 open reading frame 54 | ---0.714 | 0.0121144 | 0.0121 |
| 1562674_at | LOC101929325 | uncharacterized LOC101929325 | ---0.653 | 0.0259115 | 0.0253 |
| 1562922_at | LOC101929133 | uncharacterized LOC101929133 | ---0.607 | 0.040002 | 0.0402 |
| 1564443_at | DLEU2 | deleted in lymphocytic leukemia 2 (non-protein coding) | 0.635 | 0.0301143 | 0.0302 |
| 1565849_a_at | SRRM5 | serine/arginine repetitive matrix 5 | ---0.695 | 0.0158777 | 0.0148 |
| 1566633_at | --- | --- | ---0.642 | 0.0279546 | 0.0273 |
| 1567333_at | --- | --- | 0.62 | 0.0348001 | 0.0345 |
| 1568999_at | PSG4 /// PSG6 | pregnancy specific beta-1-glycoprotein 4 /// pregnancy specific beta-1-glycoprotein 6 | ---0.719 | 0.0110205 | 0.0103 |
| 1570342_at | NKTR | natural killer cell triggering receptor | 0.642 | 0.0279546 | 0.0295 |
| 200054_at | ZPR1 | ZPR1 zinc finger | ---0.67 | 0.0204419 | 0.0218 |
| 201076_at | SNU13 | SNU13 homolog, small nuclear ribonucleoprotein (U4/U6.U5) | ---0.642 | 0.0279546 | 0.0277 |
| 201621_at | NBL1 | neuroblastoma 1, DAN family BMP antagonist | ---0.768 | 0.0052529 | 0.0051 |
| 201870_at | TOMM34 | translocase of outer mitochondrial membrane 34 | ---0.6 | 0.0428069 | 0.0401 |
| 202737_s_at | LSM4 | LSM4 homolog, U6 small nuclear RNA and mRNA degradation associated | ---0.726 | 0.0100009 | 0.0091 |
| 203815_at | GSTT1 | glutathione S-transferase theta 1 | ---0.698 | 0.0145393 | 0.0143 |
| 204683_at | ICAM2 | intercellular adhesion molecule 2 | ---0.723 | 0.0110205 | 0.0101 |
| 205978_at | KL | klotho | 0.737 | 0.0090521 | 0.0103 |
| 206043_s_at | ATP2C2 | ATPase, Ca++ transporting, type 2C, member 2 | 0.6 | 0.0428069 | 0.0413 |
| 206372_at | MYF6 | myogenic factor 6 (herculin) | ---0.628 | 0.0323947 | 0.0318 |
| 206498_at | OCA2 | oculocutaneous albinism II | ---0.61 | 0.040002 | 0.038 |
| 206588_at | DAZL | deleted in azoospermia-like | 0.632 | 0.0323947 | 0.0327 |
| 206964_at | NAT8B | N-acetyltransferase 8B (GCN5-related, putative, gene/pseudogene) | ---0.621 | 0.0348001 | 0.0341 |
| 207247_s_at | ZFX /// ZFY | zinc finger protein, X-linked /// zinc finger protein, Y-linked | 0.684 | 0.017305 | 0.0176 |
| 207771_at | SLC5A2 | solute carrier family 5 (sodium/glucose cotransporter), member 2 | ---0.79 | 0.0036168 | 0.0043 |
| 208464_at | GRIA4 | glutamate receptor, ionotropic, AMPA 4 | ---0.782 | 0.0041155 | 0.0032 |
| 209834_at | CHST3 | carbohydrate (chondroitin 6) sulfotransferase 3 | 0.614 | 0.0373345 | 0.0378 |
| 210268_at | NFX1 | nuclear transcription factor, X-box binding 1 | 0.919 | < 1e---07 | 1.00E---04 |
| 211126_s_at | CSRP2 | cysteine and glycine-rich protein 2 | ---0.593 | 0.0457531 | 0.0431 |
| 211436_at | --- | --- | ---0.706 | 0.0132861 | 0.0129 |
| 212924_s_at | LSM4 | LSM4 homolog, U6 small nuclear RNA and mRNA degradation associated | ---0.604 | 0.0428069 | 0.0426 |
| 215296_at | CDC42BPA | CDC42 binding protein kinase alpha (DMPK-like) | ---0.646 | 0.0279546 | 0.0283 |
| 215443_at | TSHR | thyroid stimulating hormone receptor | ---0.677 | 0.0188251 | 0.0172 |
| 217343_at | --- | --- | ---0.586 | 0.0488449 | 0.0475 |
| 217416_x_at | --- | --- | 0.758 | 0.0065965 | 0.006 |
| 217540_at | NXPE3 | neurexophilin and PC-esterase domain family, member 3 | 0.747 | 0.0073532 | 0.0073 |
| 217917_s_at | DYNLRB1 | dynein, light chain, roadblock-type 1 | ---0.593 | 0.0457531 | 0.0481 |
| 218066_at | SLC12A7 | solute carrier family 12 (potassium/chloride transporter), member 7 | ---0.691 | 0.0158777 | 0.0151 |
| 218801_at | UGGT2 | UDP-glucose glycoprotein glucosyltransferase 2 | ---0.621 | 0.0348001 | 0.0327 |
| 218931_at | RAB17 | RAB17, member RAS oncogene family | 0.628 | 0.0323947 | 0.0333 |
| 219419_at | RBFA | ribosome binding factor A (putative) | ---0.677 | 0.0188251 | 0.0186 |
| 220241_at | TMCO3 | transmembrane and coiled-coil domains 3 | 0.779 | 0.0046599 | 0.0034 |
| 221515_s_at | LCMT1 | leucine carboxyl methyltransferase 1 | ---0.61 | 0.040002 | 0.0425 |
| 221692_s_at | MRPL34 | mitochondrial ribosomal protein L34 | 0.639 | 0.0301143 | 0.0307 |
| 222013_x_at | EEF2KMT | eukaryotic elongation factor 2 lysine methyltransferase | ---0.782 | 0.0041155 | 0.0038 |
| 222481_at | TIMM10B | translocase of inner mitochondrial membrane 10 homolog B (yeast) | ---0.758 | 0.0065965 | 0.0043 |
| 223367_at | DNAJC30 | DnaJ (Hsp40) homolog, subfamily C, member 30 | ---0.639 | 0.0301143 | 0.032 |
| 223640_at | HCST | hematopoietic cell signal transducer | ---0.646 | 0.0279546 | 0.0255 |
| 223779_at | AFAP1-AS1 | AFAP1 antisense RNA 1 | ---0.656 | 0.0239811 | 0.0224 |
| 224137_at | CACNG7 | calcium channel, voltage-dependent, gamma subunit 7 | ---0.688 | 0.017305 | 0.0173 |
| 224885_s_at | KRTCAP2 | keratinocyte associated protein 2 | ---0.8 | 0.0031613 | 0.0024 |
| 225465_at | MAGI1 | membrane associated guanylate kinase, WW and PDZ domain containing 1 | 0.667 | 0.0221592 | 0.0219 |
| 225868_at | TRIM47 | tripartite motif containing 47 | ---0.674 | 0.0204419 | 0.0206 |
| 225979_at | PLEKHG2 | pleckstrin homology domain containing, family G (with RhoGef domain) member 2 | ---0.604 | 0.0428069 | 0.042 |
| 226565_at | TMEM99 | transmembrane protein 99 | ---0.642 | 0.0279546 | 0.0277 |
| 227004_at | CDKL5 | cyclin-dependent kinase-like 5 | 0.6 | 0.0428069 | 0.0372 |
| 227300_at | TMEM119 | transmembrane protein 119 | ---0.698 | 0.0145393 | 0.0142 |
| 227779_at | ECSCR | endothelial cell surface expressed chemotaxis and apoptosis regulator | ---0.59 | 0.0488449 | 0.0479 |
| 228181_at | SLC30A1 | solute carrier family 30 (zinc transporter), member 1 | 0.593 | 0.0457531 | 0.0453 |

| | | | | | |
|---|---|---|---|---|---|
| 228327_x_at | MEIS3 | Meis homeobox 3 | ---0.586 | 0.0488449 | 0.0498 |
| 229247_at | FBLN7 | fibulin 7 | ---0.632 | 0.0323947 | 0.0293 |
| 229301_at | --- | --- | 0.642 | 0.0279546 | 0.0309 |
| 229339_at | MYOCD | myocardin | 0.705 | 0.0132861 | 0.0124 |
| 230222_at | HIVEP1 | human immunodeficiency virus type I enhancer binding protein 1 | 0.607 | 0.040002 | 0.0397 |
| 230378_at | SCGB3A1 | secretoglobin, family 3A, member 1 | ---0.67 | 0.0204419 | 0.0221 |
| 230905_at | --- | --- | ---0.61 | 0.040002 | 0.0409 |
| 231060_at | --- | --- | ---0.596 | 0.0457531 | 0.0458 |
| 232087_at | CXorf23 | chromosome X open reading frame 23 | 0.677 | 0.0188251 | 0.0198 |
| 232149_s_at | NSMAF | neutral sphingomyelinase activation associated factor | 0.702 | 0.0145393 | 0.0157 |
| 232156_at | AP5M1 | adaptor-related protein complex 5, mu 1 subunit | 0.689 | 0.0158777 | 0.0171 |
| 233306_at | --- | --- | ---0.667 | 0.0221592 | 0.0214 |
| 233436_at | MTBP | MDM2 binding protein | ---0.628 | 0.0323947 | 0.0316 |
| 233562_at | LINC00839 | long intergenic non-protein coding RNA 839 | ---0.596 | 0.0457531 | 0.0457 |
| 233850_s_at | EBF4 | early B-cell factor 4 | ---0.863 | 0.0005971 | 4.00E---04 |
| 234126_at | --- | --- | 0.628 | 0.0323947 | 0.0317 |
| 234357_at | --- | --- | 0.814 | 0.0023689 | 0.0024 |
| 235184_at | AEBP2 | AE binding protein 2 | 0.656 | 0.0239811 | 0.0243 |
| 235334_at | ST6GALNAC3 | ST6 (alpha-N-acetyl-neuraminyl-2,3-beta-galactosyl-1,3)-N-acetylgalactosaminide alpha-2,6-sialyltransferase 3 | 0.674 | 0.0204419 | 0.0191 |
| 236129_at | GALNT5 | polypeptide N-acetylgalactosaminyltransferase 5 | ---0.621 | 0.0348001 | 0.0318 |
| 236763_at | --- | --- | 0.607 | 0.040002 | 0.0419 |
| 236775_s_at | --- | --- | ---0.622 | 0.0348001 | 0.0361 |
| 237191_x_at | --- | --- | ---0.646 | 0.0279546 | 0.0298 |
| 237712_at | LOC100507562 | uncharacterized LOC100507562 | ---0.628 | 0.0323947 | 0.0321 |
| 237735_at | CCDC37-AS1 | CCDC37 antisense RNA 1 (head to head) | 0.731 | 0.0090521 | 0.0102 |
| 237807_at | SPATA12 | spermatogenesis associated 12 | ---0.634 | 0.0301143 | 0.0274 |
| 238243_at | --- | --- | 0.716 | 0.0121144 | 0.0132 |
| 238844_s_at | NPHP1 | nephronophthisis 1 (juvenile) | ---0.656 | 0.0239811 | 0.0253 |
| 239106_at | CA5BP1 | carbonic anhydrase VB pseudogene 1 | 0.625 | 0.0348001 | 0.0342 |
| 239417_x_at | C6orf52 | chromosome 6 open reading frame 52 | ---0.758 | 0.0065965 | 0.007 |
| 239913_at | SLC10A4 | solute carrier family 10, member 4 | 0.779 | 0.0046599 | 0.004 |
| 239944_at | --- | --- | 0.663 | 0.0221592 | 0.0216 |
| 240103_at | --- | --- | 0.646 | 0.0279546 | 0.0288 |
| 241440_at | LOC440982 /// ZIC1 | uncharacterized LOC440982 /// Zic family member 1 | ---0.618 | 0.0373345 | 0.0357 |
| 242378_at | MYADML2 | myeloid-associated differentiation marker-like 2 | ---0.67 | 0.0204419 | 0.021 |
| 242905_at | PNO1 | partner of NOB1 homolog | 0.747 | 0.0073532 | 0.0068 |
| 243010_at | MSI2 | musashi RNA binding protein 2 | ---0.772 | 0.0052529 | 0.0049 |
| 243543_at | --- | --- | ---0.621 | 0.0348001 | 0.0343 |
| 243570_at | SPCS2 | signal peptidase complex subunit 2 | 0.751 | 0.0073532 | 0.0077 |
| 243843_at | N4BP2L1 | NEDD4 binding protein 2-like 1 | 0.782 | 0.0041155 | 0.0034 |
| 243925_at | --- | --- | 0.695 | 0.0158777 | 0.0162 |
| 244113_at | KCNJ9 | potassium channel, inwardly rectifying subfamily J, member 9 | ---0.793 | 0.0036168 | 0.0024 |
| 244297_at | FAM95C | family with sequence similarity 95, member C | ---0.698 | 0.0145393 | 0.0133 |
| 244449_at | LOC102723380 /// LOC102724370 | uncharacterized LOC102723380 /// uncharacterized LOC102724370 | ---0.898 | 5.90E---06 | 3.00E---04 |
| 244740_at | --- | --- | ---0.782 | 0.0041155 | 0.0036 |

Table S4: **Identification of Blood Transcription Modules (BTM) significantly associated with BP response in hypertensive patients.** The comparative analysis for enrichment of BTM in responders and non-responders was performed using the GSEA approach. Red and green color depict the modules with up- and down-regulation patterns, respectively, in hypertension responders compared to non-responders. Modules with significant p value (<.05) and False Discovery rate (<25%) are shown in bold font.

**Table S4:** Identification of **B**lood **T**ranscription **M**odules (BTM) significantly associated with BP Response in hypertensive patients.

| NAME | NES | P-Val |
|---|---|---|
| T CELL SURFACE SIGNATURE (S0) | -1.69 | 0.00 |
| PLASMA CELL SURFACE SIGNATURE (S3) | -1.68 | 0.00 |
| ENRICHED FOR CELL MIGRATION (M122) | -1.71 | 0.00 |
| T CELL DIFFERENTIATION (TH2) (M19) | -1.73 | 0.01 |
| ENRICHED FOR PROMOTER MOTIF NATCACGTGAY (PUTATIVE SREBF1 TARGETS) (M178) | -1.67 | 0.01 |
| ENRICHED IN B CELLS (IV) (M47.3) | -1.58 | 0.01 |
| ENRICHED IN MEMBRANE PROTEINS (M124) | -1.57 | 0.02 |
| PLATELET ACTIVATION & BLOOD COAGULATION (M199) | -1.56 | 0.03 |
| TRANSCRIPTION ELONGATION, RNA POLYMERASE II (M234) | -1.55 | 0.03 |
| ACTIVATED (LPS) DENDRITIC CELL SURFACE SIGNATURE (S11) | -1.44 | 0.03 |
| ADHESION AND MIGRATION, CHEMOTAXIS (M91) | -1.53 | 0.04 |
| TRANSCRIPTIONAL TARGETS OF GLUCOCORTICOID RECEPTOR (M74) | -1.51 | 0.04 |
| CELL ACTIVATION (IL15, IL23, TNF) (M24) | -1.49 | 0.04 |
| EXTRACELLULAR REGION CLUSTER (GO) (M189) | -1.40 | 0.07 |
| CELL ADHESION (LYMPHOCYTE HOMING) (M21) | -1.39 | 0.08 |
| ENRICHED IN T CELLS (I) (M7.0) | -1.34 | 0.08 |
| LEUKOCYTE MIGRATION (M88.0) | -1.33 | 0.08 |
| MYELOID, DENDRITIC CELL ACTIVATION VIA NFKB (I) (M43.0) | -1.42 | 0.09 |
| **INOSITOL PHOSPHATE METABOLISM (M129)** | **1.92** | **0.00** |
| REGULATION OF TRANSCRIPTION, TRANSCRIPTION FACTORS (M213) | 1.72 | 0.00 |
| CELL CYCLE (I) (M4.1) | 1.41 | 0.03 |
| E2F TRANSCRIPTION FACTOR NETWORK (M8) | 1.57 | 0.03 |
| MITOTIC CELL CYCLE IN STIMULATED CD4 T CELLS (M4.5) | 1.47 | 0.03 |
| HOX CLUSTER I (M17.0) | 1.60 | 0.03 |
| CELL CYCLE, MITOTIC PHASE (M230) | 1.52 | 0.04 |
| HOX CLUSTER III (M17.2) | 1.56 | 0.05 |
| INTEGRIN MEDIATED LEUKOCYTE MIGRATION (M39) | 1.61 | 0.05 |
| CELL DIVISION IN STIMULATED CD4 T CELLS (M4.6) | 1.49 | 0.06 |
| LIPID METABOLISM, ENDOPLASMIC RETICULUM (M92) | 1.56 | 0.06 |
| MITOTIC CELL CYCLE IN STIMULATED CD4 T CELLS (M4.9) | 1.40 | 0.08 |

Table S5: **List of genes that are identified to be associated with BP response in hypertensive patients.** These genes were identified from differential expression and GSEA analysis. Up- and down-regulated genes are shown in red and green color, respectively.

**Table S5:** List of genes that are identified to be associated with BP response in hypertensive patients.

| Upregulated Genes from GSEA & Differential Exp Aanalysis | | Down-regulated Genes from GSEA & Differential Exp Aanalysis | |
|---|---|---|---|
| Gene | Description | Gene | Description |
| AEBP2 | AE binding protein 2 | AFAP1-AS1 | AFAP1 antisense RNA 1 |
| ANKRD18A | ankyrin repeat domain 18A | ALDH2 | aldehyde dehydrogenase 2 family (mitochondrial) |
| ARNTL | aryl hydrocarbon receptor nuclear translocator-like | ALDH3A2 | aldehyde dehydrogenase 3 family, member A2 |
| ATP2C2 | ATPase, Ca++ transporting, type 2C, member 2 | ALDH7A1 | aldehyde dehydrogenase 7 family, member A1 |
| CCDC37-AS1 | CCDC37 antisense RNA 1 (head to head) | ALDH9A1 | aldehyde dehydrogenase 9 family, member A1 |
| CDH1 | cadherin 1, type 1, E-cadherin (epithelial) | C15orf54 | chromosome 15 open reading frame 54 |
| CDH11 | cadherin 11, type 2, OB-cadherin (osteoblast) | C6orf52 | chromosome 6 open reading frame 52 |
| CDH12 | cadherin 12, type 2 (N-cadherin 2) | CA5BP1 | carbonic anhydrase VB pseudogene 1 |
| CDH18 | cadherin 18, type 2 | CACNG7 | calcium channel, voltage-dependent, gamma subunit 7 |
| CDH2 | cadherin 2, type 1, N-cadherin (neuronal) | CD40 | CD40 molecule, TNF receptor superfamily member 5 |
| CDH9 | cadherin 9, type 2 (T1-cadherin) | CD40LG | CD40 ligand |
| CHST3 | carbohydrate (chondroitin 6) sulfotransferase 3 | CDC42BPA | CDC42 binding protein kinase alpha (DMPK-like) |
| CLDN1 | claudin 1 | CEL | carboxyl ester lipase |
| CLOCK | clock circadian regulator | CSRP2 | cysteine and glycine-rich protein 2 |
| CREBBP | CREB binding protein | DNAJC30 | DnaJ (Hsp40) homolog, subfamily C, member 30 |
| CTNNB1 | catenin (cadherin-associated protein), beta 1, 88kDa | DYNLRB1 | dynein, light chain, roadblock-type 1 |
| CTNND1 | catenin (cadherin-associated protein), delta 1 | EBF4 | early B-cell factor 4 |
| CXorf23 | chromosome X open reading frame 23 | ECSCR | endothelial cell surface expressed chemotaxis and apoptosis regulator |
| DAZL | deleted in azoospermia-like | ENAH | enabled homolog (Drosophila) |
| DLEU2 | deleted in lymphocytic leukemia 2 (non-protein coding) | FAM86A | family with sequence similarity 86, member A |
| EP300 | E1A binding protein p300 | FBLN7 | fibulin 7 |
| FYN | FYN oncogene related to SRC, FGR, YES | GALNT5 | polypeptide N-acetylgalactosaminyltransferase 5 |
| GAB1 | GRB2-associated binding protein 1 | GRIA4 | glutamate receptor, ionotropic, AMPA 4 |
| GRB2 | growth factor receptor-bound protein 2 | GSTT1 | glutathione S-transferase theta 1 |
| HOMER2 | homer homolog 2 (Drosophila) | HCST | hematopoietic cell signal transducer |
| IQGAP1 | IQ motif containing GTPase activating protein 1 | HELB | helicase (DNA) B |
| JAK1 | Janus kinase 1 | HLA-DMA | major histocompatibility complex, class II, DM alpha |
| JUN | jun proto-oncogene | HLA-DMB | major histocompatibility complex, class II, DM beta |
| KL | klotho | HLA-DOA | major histocompatibility complex, class II, DO alpha |
| KPNA1 | karyopherin alpha 1 (importin alpha 5) | HLA-DPA1 | major histocompatibility complex, class II, DP alpha 1 |
| LETM2 | leucine zipper-EF-hand containing transmembrane protein 2 | HLA-DPB1 | major histocompatibility complex, class II, DP beta 1 |
| LOC1005074 | uncharacterized LOC100507419 | ICAM2 | intercellular adhesion molecule 2 |
| LOC1009964 | uncharacterized LOC100996457 | IFNA1/IFNA1 | interferon, alpha 1 |
| LOC1019293 | uncharacterized LOC101929325 | IFNB1 | interferon, beta 1, fibroblast |
| MAP2K6 | mitogen-activated protein kinase kinase 6 | IL12A | interleukin 12A |
| MAPK14 | mitogen-activated protein kinase 14 | IL13 | interleukin 13 |
| MAPK8 | mitogen-activated protein kinase 8 | KCNJ9 | potassium inwardly-rectifying channel, subfamily J, member 9 |
| MED26 | mediator complex subunit 26 | KRTCAP2 | keratinocyte associated protein 2 |
| MSI2 | musashi RNA-binding protein 2 | LCMT1 | leucine carboxyl methyltransferase 1 |
| MTBP | MDM2 binding protein | LINC00662 | long intergenic non-protein coding RNA 662 |
| MTM1 | myotubularin 1 | LINC00663 | long intergenic non-protein coding RNA 663 |

| | | | |
|---|---|---|---|
| MTMR1 | myotubularin related protein 1 | LINC00839 | long intergenic non-protein coding RNA 839 |
| MYOCD | myocardin | LOC1005070 | uncharacterized LOC100507054 |
| N4BP2L1 | NEDD4 binding protein 2-like 1 | LOC1005075 | uncharacterized LOC100507562 |
| NCOA1 | nuclear receptor coactivator 1 | LOC1019291 | uncharacterized LOC101929133 |
| NCOA3 | nuclear receptor coactivator 3 | LSM4 | LSM4 homolog, U6 small nuclear RNA associated (S. cerevisiae) |
| NCOR1 | nuclear receptor corepressor 1 | MAGI1 | membrane associated guanylate kinase, WW and PDZ domain containing 1 |
| NKTR | natural killer cell triggering receptor | MEIS3 | Meis homeobox 3 |
| NPAS2 | neuronal PAS domain protein 2 | MINOS1-NBL | neuroblastoma 1, DAN family BMP antagonist |
| NR2C2 | nuclear receptor subfamily 2, group C, member 2 | MKL2 | MKL/myocardin-like 2 |
| NSMAF | neutral sphingomyelinase (N-SMase) activation associated factor | MRAP | melanocortin 2 receptor accessory protein |
| NUP153 | nucleoporin 153kDa | MRPL34 | mitochondrial ribosomal protein L34 |
| NUP188 | nucleoporin 188kDa | MYADML2 | myeloid-associated differentiation marker-like 2 |
| NUP43 | nucleoporin 43kDa | MYF6 | myogenic factor 6 (herculin) |
| NUP50 | nucleoporin 50kDa | NAT8B | N-acetyltransferase 8B (GCN5-related, putative, gene/pseudogene) |
| NUPL1 | nucleoporin like 1 | NFX1 | nuclear transcription factor, X-box binding 1 |
| NXPE3 | neurexophilin and PC-esterase domain family, member 3 | NHP2L1 | NHP2 non-histone chromosome protein 2-like 1 (S. cerevisiae) |
| PIK3C2A | phosphatidylinositol-4-phosphate 3-kinase, catalytic subunit type 2 alpha | NPHP1 | nephronophthisis 1 (juvenile) |
| PIK3C2B | phosphatidylinositol-4-phosphate 3-kinase, catalytic subunit type 2 beta | OCA2 | oculocutaneous albinism II |
| PIK3CB | phosphatidylinositol-4,5-bisphosphate 3-kinase, catalytic subunit beta | PDZD3 | PDZ domain containing 3 |
| PIK3CD | phosphatidylinositol-4,5-bisphosphate 3-kinase, catalytic subunit delta | PITPNM2 | phosphatidylinositol transfer protein, membrane-associated 2 |
| PIK3R1 | phosphoinositide-3-kinase, regulatory subunit 1 (alpha) | PLEKHG2 | pleckstrin homology domain containing, family G (with RhoGef domain) member 2 |
| PIP5K1C | phosphatidylinositol-4-phosphate 5-kinase, type I, gamma | PSG4 | pregnancy specific beta-1-glycoprotein 4 |
| PNO1 | partner of NOB1 homolog (S. cerevisiae) | RAB17 | RAB17, member RAS oncogene family |
| POM121/PO | POM121 transmembrane nucleoporin | RBFA | ribosome binding factor A (putative) |
| PSMD6 | proteasome (prosome, macropain) 26S subunit, non-ATPase, 6 | SCGB3A1 | secretoglobin, family 3A, member 1 |
| PTK2B | protein tyrosine kinase 2 beta | SLC12A7 | solute carrier family 12 (potassium/chloride transporter), member 7 |
| PTPN11 | protein tyrosine phosphatase, non-receptor type 11 | SLC30A1 | solute carrier family 30 (zinc transporter), member 1 |
| PVRL2 | poliovirus receptor-related 2 (herpesvirus entry mediator B) | SLC5A2 | solute carrier family 5 (sodium/glucose cotransporter), member 2 |
| PVRL3 | poliovirus receptor-related 3 | SPATA12 | spermatogenesis associated 12 |
| RANBP2 | RAN binding protein 2 | SRRM5 | serine/arginine repetitive matrix 5 |
| RAP1A | RAP1A, member of RAS oncogene family | TIMD4 | T-cell immunoglobulin and mucin domain containing 4 |
| RAP1B | RAP1B, member of RAS oncogene family | TIMM10B | translocase of inner mitochondrial membrane 10 homolog B (yeast) |
| RAPGEF1 | Rap guanine nucleotide exchange factor (GEF) 1 | TMEM99 | transmembrane protein 99 |
| SLC10A4 | solute carrier family 10, member 4 | TNF | tumor necrosis factor |
| SLC9B1 | solute carrier family 9, subfamily B (NHA1, cation proton antiporter 1), member 1 | TOMM34 | translocase of outer mitochondrial membrane 34 |
| SPCS2 | signal peptidase complex subunit 2 homolog (S. cerevisiae) | TRIM47 | tripartite motif containing 47 |
| ST6GALNAC3 | ST6 (alpha-N-acetyl-neuraminyl-2,3-beta-galactosyl-1,3)-N-acetylgalactosaminide | TUG1 | taurine up-regulated 1 (non-protein coding) |
| STAT3 | signal transducer and activator of transcription 3 (acute-phase response factor) | UGGT2 | UDP-glucose glycoprotein glucosyltransferase 2 |
| STAT5A | signal transducer and activator of transcription 5A | ZDHHC4 | zinc finger, DHHC-type containing 4 |
| SYNJ2 | synaptojanin 2 | ZFY | zinc finger protein, Y-linked |
| TBL1X | transducin (beta)-like 1X-linked | ZIC1 | Zic family member 1 |
| TLL1 | tolloid-like 1 | ZNF667-AS1 | ZNF667 antisense RNA 1 (head to head) |
| TLN1 | talin 1 | ZPR1 | ZPR1 zinc finger |

| | | | |
|---|---|---|---|
| TMCO3 | transmembrane and coiled-coil domains 3 | | |
| TMEM119 | transmembrane protein 119 | | |
| TPR | translocated promoter region, nuclear basket protein | | |
| TSHR | thyroid stimulating hormone receptor | | |
